# An expanded RT-PCR melting temperature coding assay to rapidly identify all known SARS-CoV-2 variants and sub-variants of concern

**DOI:** 10.1038/s41598-023-48647-8

**Published:** 2023-12-11

**Authors:** Padmapriya P. Banada, Raquel Green, Deanna Streck, Rohini Kurvathi, Robert Reiss, Sukalyani Banik, Naranjargal Daivaa, Ibsen Montalvan, Robert Jones, Salvatore A. E. Marras, Soumitesh Chakravorty, David Alland

**Affiliations:** 1 Rutgers New Jersey Medical School, Public Health Research Institute, Newark, NJ USA; 2 Institute of Genomic Medicine, Rutgers New Jersey Medical School, Newark, NJ USA; 3 University Hospital, Newark, NJ USA; 4 Division of Infectious Diseases, Department of Medicine, Rutgers New Jersey Medical School, Newark, NJ USA; 5 Craic Computing LLC, Snohomish, WA USA; 6 Cepheid, Sunnyvale, CA USA

**Keywords:** Biological techniques, Microbiology, Diseases, Medical research

## Abstract

The continued emergence of vaccine-resistant SARS-CoV-2 variants of concern (VOC) requires specific identification of each VOC as it arises. Here, we report an expanded version of our previously described sloppy molecular beacon (SMB) melting temperature (Tm) signature-based assay for VOCs, now modified to include detection of Delta (B.1.617.2) and Omicron (B.1.1.529) sub-variants. The SMB-VOC assay targets the signature codons 501, 484 and 452 in the SARS-CoV-2 spike protein which we show can specifically detect and differentiate all known VOCs including the Omicron subvariants (BA.1, BA.2, BA.2.12.1, BA.4/BA.5). The limit of detection (LOD) of the assay was 20, 22 and 36 genomic equivalents (GE) per reaction with the Delta, Omicron BA.1 and BA.2 respectively. Clinical validation of the 3-codon assay in the LC480 instrument showed the assay detected 94% (81/86) of the specimens as WT or VOCs and 6% (5/86) of the tests producing indeterminate results compared to sequencing. Sanger sequencing also failed for four samples. None of the specimens were incorrectly identified as WT or as a different VOC by our assay. Thus, excluding specimens with indeterminant results, the assay was 100% sensitive and 100% specific compared to Sanger sequencing for variant identification. This new assay concept can be easily expanded to add newer variants and can serve as a robust diagnostic tool for selecting appropriate monoclonal antibody therapy and rapid VOC surveillance.

## Introduction

The global emergence of SARS-CoV-2 variants of concern (VOC), B.1.1.7 (20I, Alpha), B.1.351 (20H, Beta), P.1 (20J, Gamma), B.1.617.2 (21A, Delta) and subvariants of Omicron B.1.1.529∕ 21M (21 K/BA.1; 21L/BA.2; 22C/BA.2.12.1; 22A/BA.4; 22B/BA.5; 22D/BA.2.75; 22E/BQ.1; 22F/XBB), have been responsible for a series of surges in reported COVID-19 cases^1–7^. The Omicron variant was first identified as a VOC by the WHO in November 2021^8^, and is now comprised of several sub lineages^3, 9^. By Aug 25, 2022, Omicron BA.5 dominated the United States accounting for 84.2% of the COVID-19 cases, followed by Omicron BA.4 at 13.3%, Omicron BA.2.12.1 at 1.2%, Omicron BA.2 at 0.8%, and Omicron BA.2.75 at 0.4%^10^. In the beginning of 2023, XBB.1.5 (Kraken) became the prominent VOC with 61.3% cases as per CDC’s nowcast and weighted estimates^11^, and new variants continue to emerge. Several studies have indicated that major SARS-CoV-2 variants may be more transmissible and possibly more virulent than other SARS-CoV-2 strains^12–15^. SARS-CoV-2 variants also may confer resistance to therapeutics and result in decreased vaccine efficacy^15–20^ due to the presence of key mutations in the spike protein^21–26^.

Although the United States has recently developed an increased capacity to track Omicron variants by whole genome sequencing, only 2–10% of COVID-19 cases are currently being sequenced and the results of these efforts are often delayed by up to 10 days^11^, threatening their utility in preventing further spread or providing real-time therapeutic guidance. Certain RT-PCR COVID-19 tests can rapidly identify Omicron variants when a positive assay fails to detect the S-gene target sequences (SGTF)^1^ or ORF1ab or N-gene^27^. These target failure assays have questionable performance and are limited in that they may only identify Omicron variants, and still require sequence confirmation. On the other hand, assays that specifically target mutations at and near codons 484, 452, and 501 of the SARS-CoV-2 spike gene may be a more sensitive and specific way to identify and differentiate VOC and other Omicron sub-lineages^28, 29^. We have previously demonstrated an RT-PCR based method that uses sloppy molecular beacons (SMBs) combined with melting temperature (Tm) code analysis to detect mutations in codons 501 and 484 of the SARS-CoV-2 spike protein^30^. We showed that this assay could specifically identify WT SARS-CoV-2 and the Alpha, Beta and Gamma variants. Delta and the Omicron subvariants have developed additional variant defining mutations and cannot be identified by our original assay; however, each of these VOCs along with Omicron subvariants can be specifically detected by identifying a relatively small number of mutations in the SARS-CoV-2 spike protein^13, 21, 31, 32^. Our SMB-VOC assay is particularly well suited for identifying multiple different mutations in short genomic regions with high accuracy^33, 34^, enabling us to design an expanded assay to identify and distinguish Delta, Omicron and its subvariants (BA.1, BA.2/2.75, BA.2.12.1, and BA.4/5) along with the older VOCs (Alpha, Beta, and Gamma). Here, we demonstrate that this approach is flexible and can be used for detecting VOCs using both SARS-CoV-2 RNA and clinical specimens. We further confirmed assay performance in four different RT-PCR instruments commonly available in diagnostic laboratories. Adaptation of our assay will enable real-time detection of SARS-CoV-2 variant spread, without the need for whole genome sequencing on all specimens.

## Results

### Overall design

The overall SMB-VOC assay consists of several component assays, each targeting a different spike protein codon with a pair of two related sloppy molecular beacons (SMBs, Table 1S), as explained previously^30^. For each codon, one of the paired SMBs is complementary to the codon’s wild type (WT) sequence and the other is complementary to a relevant mutant (MT) sequence. A post-PCR melt analysis of each paired SMB is then used to identify the specific mutation at or near each codon, using the pattern of Tm values generated by the WT and MT SMB. The combined assay includes 3 paired SMB assays that target WT or MT alleles at and near the 3 codons 452, 484 and 501 (Table 1). Each VOC is then specifically identified according to their unique pattern of WT and MT alleles across all the component assays.

**Table 1:** List of mutations detected by the SMB-VOC assay from all known variants of concern (VOC).

SARS-CoV-2 variants of concern	Mutations detected by:		
	SMB-501 assay	SMB-484 assay	SMB-452 assay
Alpha	N501Y	E484 (WT)	L452 (WT)
Beta	N501Y	E484K	L452 (WT)
Delta	N501 (WT)	E484 (WT)	L452R
Omicron: BA.1	G496S, Q498R, N501Y, Y505H	E484A	L452 (WT)
Omicron: BA.2/BA.2.75	Q498R, N501Y, Y505H	E484A	L452 (WT)
Omicron: BA.2.12.1	Q498R, N501Y, Y505H	E484A	L452Q
Omicron: BA.4/5/BQ1.1	Q498R, N501Y, Y505H	E484A, F486V	L452R
XBB.1.5	Q498R, N501Y, Y505H	E484A, F486P	L452 (WT)

### Developing Tm code definitions

We tested the complete assay against SARS-CoV-2 reference strains for each VOC. The resulting Tm values for each assay component, produced by each of the paired SMBs assays, are listed in Tables 2 and 2S. The Tm values varied slightly (± 2.0) between specimens and different instrument platforms (Table 2S). The paired Tm coding approach provides for a robust sequence identification even in the presence of these Tm fluctuations as shown in Table 2. For example, in the LC480 instrument, both USA WA1/2020 (WT strain) and Delta strains have the same WT 501N allele which results in a mean Tm of 59.7 ± 0.1 °C for the 501-WT probe and 58.9 ± 0.07 °C with the 501-MT probe, but Alpha and Beta have a mutant 501Y allele, which results in a mean Tm of 55.7 ± 0.04 °C for the 501-WT probe and 62.6 ± 0.08 °C with the 501-MT probe. Omicron BA.1 has 3 mutations (G496S, Q498R, Y505H) in the 501-probe binding region, in addition to the highly mutant 501Y allele which results in a mean Tm of 49 °C ± 0.12 °C for the 501-WT probe and 56.6 ± 0.15 °C for the 501-MT probe. The other Omicron subvariants (BA.2/BA.2.12.1/BA.4/5) lack the 496S mutation and carry Q498R, N501Y, and Y505H mutations on the 501-probe binding region, thus the Tm generated by the 501-WT probe is 50.6 ± 0.10 °C and the Tm generated by the 501-MT probe is 58.5 ± 0.06 °C. Similarly, Tm codes were established for the other two assays (SMB-484 and SMB-452) targeting mutations around these codons, for both WT and variant alleles as also shown in Table 2. The six components Tm codes generated by all three assays from 18 different reference strains (BEI resources, Table 2) were recorded and used as reference Tm signatures for each VOC.

**Table 2:** Reference Tm signature profile from the SMB-VOC assay probes and validation of the SMB-VOC assay using patient specimens in Roche LC480.

Reference strains/Patient samples	Source	PCR Ct	IC Ct	SMB-501 Assay		SMB-484AKw Assay		SMB-452 Assay		Final id by SMB VOC assay	Confirmation by Whole genome sequencing/Sanger sequencing*
				WT (Cy3)	MT (Cy5)	WT (Cy3)	MT (Cy5)	WT (Cy3)	MT (Cy5)		
USA-WA1/2020 (WT)	BEI NR52285			59.7	58.9	65.5	60.4	61.9	59.9	Wild type (WT, Ancestral)	GenBank: MT246667.1
B.1.1.7 (Alpha)	BEI NR54000			55.7	62.6	65.5	60.4	62.5	60.4	B.1.1.7 (Alpha)	GISAID:EPI_ISL_683466
B.1.351 (Beta)	BEI NR55282			55.3	62.5	62.0	64.4	62.5	60.3	B.1.351 (Beta)	GISAID:EPI_ISL_890360
B.1.617.2 (Delta)	BEI NR55611			59.6	58.7	65.3	59.9	58.3	63.3	B.1.617.2 (Delta)	BEI^#^
B.1.1.529/BA.1 (Omicron)	BEI NR56461			49.0	56.7	63.1	60.3	62.4	59.2	B.1.1.529 (Omicron BA.1)	GISAID: EPI_ISL_7160424
B.1.1.529/BA.2 (Omicron)	BEI NR56520			50.6	58.5	63.1	60.3	62.5	60.3	B.1.1.529 (Omicron BA.2)	GISAID: EPI_ISL_8643930
B.1.1.529/BA.2.12.1 (Omicron)	BEI NR56781			50.7	58.5	62.9	60.1	60.1	61.0	B.1.1.529 (Omicron BA.2.12.1)	GISAID:EPI_ISL_11685455
B.1.1.529/BA.2 (Omicron)	BEI NR56520			50.6	58.6	62.9	60.1	62.6	60.4	B.1.1.529 (Omicron BA.2)	GISAID: EPI_ISL_8643930
B.1.1.529/BA.4 (Omicron)	BEI NR56803			51.0	58.4	58.0	55.1	58.9	63.5	B.1.1.529 (Omicron BA.4/5)	GISAID:EPI_ISL_12416220
B.1.1.529/BA.5 (Omicron)	BEI NR58616			51.0	58.6	57.8	54.9	58.8	63.8	B.1.1.529 (Omicron BA.4/5)	BEI^#^
B.1.1.529/BA.4.6 (Omicron)	BEI NR58715			51.0	58.6	57.7	54.6	58.8	63.6	B.1.1.529 (Omicron BA.4/5)	GISAID:EPI_ISL_14406648
B.1.1.529/BA.4.6 (Omicron)	BEI NR58717			50.5	58.8	58.1	55.4	58.8	63.8	B.1.1.529 (Omicron BA.4/5)	BEI^#^
B.1.1.529/BF.5 (Omicron)	BEI NR58716			51.0	58.5	57.9	55.1	58.8	63.8	B.1.1.529 (Omicron BA.4/5)	GISAID:EPI_ISL_14077403
B.1.1.529/BF.7 (Omicron)	BEI NR58974			50.5	58.6	57.8	56.1	58.9	63.9	B.1.1.529 (Omicron BA.4/5)	GISAID:EPI_ISL_15116199
B.1.1.529/BQ.1 (Omicron)	BEI NR58975			50.5	58.6	57.9	56.2	58.7	63.9	B.1.1.529 (Omicron BA.4/5)	GISAID:EPI_ISL_15313563
B.1.1.529/BQ.1.1 (Omicron)	BEI NR58976			51.1	58.8	58.2	55.5	58.7	63.8	B.1.1.529 (Omicron BA.4/5)	GISAID:EPI_ISL_15277944
B.1.1.529/XBB.1.5 (Omicron)	BEI NR59104			50.62	58.57	57.87	55.61	62.63	60.76	B.1.1.529 (Omicron XBB.1.5)	GISAID:EPI_ISL_16026423
B.1.1.529/XBB1.9 (Omicron)	BEI NR 59441			50.5	58.6	57.9	55.6	62.6	61.1	B.1.1.529 (Omicron XBB.1.5)	GISAID:EPI_ISL_17417339
VSAP1	NP	27.7	27.7	59.6	58.7	65.6	60.4	62.7	60.4	Wild type (Ancestral)	WT
VSAP2	NP	30.1	29.3	55.1	62.2	65.6	60.4	62.4	60.1	B.1.1.7 (Alpha)	B.1.1.7 (Alpha)
VSAP3	NP	30.3	29.2	60.4	59.1	65.3	59.8	59.2	64.2	B.1.617.2 (Delta)	B.1.617.2 (Delta)
VSAP4	NP	32.0	25.6	55.1	62.5	65.9	60.4	62.6	59.9	B.1.1.7 (Alpha)	B.1.1.7 (Alpha)
VSAP5	Saliva	25.7	23.7	60.4	58.5	65.8	60.5	58.5	63.5	B.1.617.2 (Delta)	B.1.617.2 (Delta)
VSAP54	NP	27.3	30.0	48.9	56.5	62.7	59.8	62.3	58.9	B.1.1.529 (Omicron BA.1)	B.1.1.529 (Omicron BA.1)
VSAP59	NP	24.2	29.9	48.9	56.5	62.8	59.9	62.3	59.0	B.1.1.529 (Omicron BA.1)	B.1.1.529 (Omicron BA.1)
VSAP75^a^	NP	NK	34.9	50.2	58.7	58.5	55.5	58.9	63.8	B.1.1.529 (Omicron BA.4/5)	B.1.1.529 (Omicron BA.5.2)
VSAP80^a^	NP	26.2	26.5	50.7	58.7	62.9	60.0	62.9	60.3	B.1.1.529 (Omicron BA.2)	B.1.1.529 (Omicron BA.2)
VSAP83	Saliva	29.6	27.0	No Tm	58.4	63.0	60.2	62.6	60.2	Pres. B.1.1.529 (Omicron BA.2)	B.1.1.529 (Omicron BA.2)
VSAP84^a^	NP	25.2	28.2	50.6	58.6	58.2	55.1	58.9	63.8	B.1.1.529 (Omicron BA.4/5)	B.1.1.529 (Omicron BA.4.2)
VSAP85^a^	NP	24.9	30.7	50.8	58.8	63.0	60.3	60.1	61.3	B.1.1.529 (Omicron BA.2.12.1)	B.1.1.529 (Omicron BA.2.12.1)
VSAP86	NP	25.8	30.3	50.7	58.8	63.0	60.4	60.1	61.3	B.1.1.529 (Omicron BA.2.12.1)	B.1.1.529 (Omicron BA.2.12.1)
VSAP89	NP	20.8	26.5	50.6	58.6	63.1	60.5	62.9	60.4	B.1.1.529 (Omicron BA.2)	B.1.1.529 (Omicron BA.2)
VSAP90^a^	NP	NK	27.9	50.4	58.7	58.4	55.4	58.9	63.8	B.1.1.529 (Omicron BA.4/5)	B.1.1.529 (Omicron BA.4.2)
N-VSAP1	NP	Neg	25.0	No Tm						SARS-CoV-2 Not Detected	Not done
N-VSAP2	NP	Neg	24.4	No Tm						SARS-CoV-2 Not Detected	Not done

Reference strains (N = 18), representative SARS-CoV-2 positive (N = 15) and negative (N = 2) patient specimens are shown here as an example of the Tm profiling and final identification of the variant strains using the SMB-VOC assay and the Excel analyze tool (Complete list of 90 positive and 9 negative specimens is shown in supplementary Table 3S).

IC, internal control assay targeting human RNaseP; NP, nasopharyngeal swab specimen; NS, Nasal Swab specimen; NK, not known; Neg, negative; VAR.IND, variant indeterminate.

*Sanger sequencing of the S-gene to confirm mutations in the probe binding regions of targeted codons.

^a^Confirmed by whole genome sequencing. #Sequences not deposited: please refer to certificate of analysis for the respective strain sequence information on BEIR webpage: https://www.beiresources.org/Catalog/animalviruses/NR-55611.aspx; https://www.beiresources.org/Catalog/animalviruses/NR-58616.aspx; https://www.beiresources.org/Catalog/animalviruses/NR-58717.aspx.

### Limit of detection

**Figure 1:**
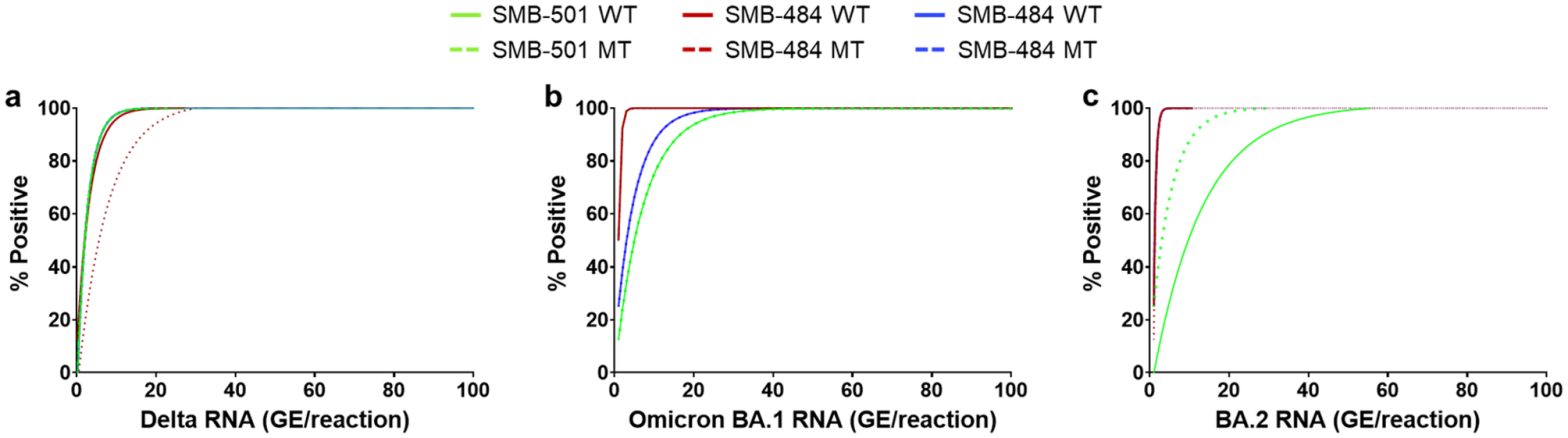
Analytical limit of detection (LOD) of the Delta (**a**), the Omicron BA.1 (**b**) and Omicron BA.2 (**c**) variants. The LOD is determined based on the non-linear regression fit curve at 95% positive rate (Graph Pad Prism 8.4.3).

The limit of detection (LOD) of the overall assay was determined by first measuring the individual LODs of each assay component (Fig. 1A) and then using these results to calculate the LOD for identifying the specific Tm signature of each VOC. We had previously defined the LOD for individual assay components that tested codons 501 and 484 for mutations that distinguish Alpha, Beta, and Gamma VOCs^30^. Delta B.1.617.2 is WT in both the SMB-501 and SMB-484 assays (Table 2). The Tm values for the 452-WT assay component (Cy3, λ533-580) and 452-MT assay component (Cy5, λ618-660) in the presence of the Delta variant target were 58.3 ± 0.12 °C and 63.2 ± 0.13 °C, respectively, defining a mutant detection signature for SMB-452 assay. The LOD of the new SMB-452 assay was established with serial dilutions of the Delta reference strain B.1.617.2 (NR-55611) RNA in negative nasal swab matrix at concentrations ranging from 200 through 0.2 genome equivalents (GE)/reaction in a Roche LC480 and was determined to be 10 GE/reaction for both the SMB-501 and SMB-484 assays and 20 GE/reaction for the SMB-452 assay. Based on the fit curves, the overall LOD combining all 3 mutation assays was found to be 20 GE/reaction (N1 Ct ~ 36) for Delta variant (Fig. 1A). Similarly, an analytical LOD was established for the SMB-VOC assay with Omicron BA.1 and BA.2 RNA at concentrations of 10^3^ through 1 GE/reaction in the presence of COVID-19 negative nasal swab matrix (Fig. 1B and C). For BA.1, the LOD for the SMB-484 assay component was 3 GE/reaction (N1 Ct > 38) and the LOD for the SMB-452 and SMB-501 assay components was determined to be 15 GE (N1 Ct ~ 36.5) and 22 GE/reaction (N1 Ct ~ 36), respectively. Similarly for the BA.2 subvariant, the LODs for the SMB-484 and SMB-452 assay components were both 3 GE/reaction (N1 Ct > 38) and the LOD for the SMB-501WT and SMB-501 MT assay components were 36 (N1 Ct ~ 35) and of 15 GE/reaction (N1 Ct ~ 36.5), respectively. All together, these individual assay component LODs resulted in a combined assay LOD of 22 GE and 36 GE/reaction for Omicron BA.1 (Fig. 1B) and Omicron BA.2 (Fig. 1C), respectively, which corresponds to an N1 Ct value of ~ 35 using the COVID-19 assay developed by the US CDC^35^. Thus, any sample identified as SARS- CoV-2 with a Ct value above 35 is unlikely to produce a VOC identification by our assay.

### Testing clinical specimens

**Figure 2:**
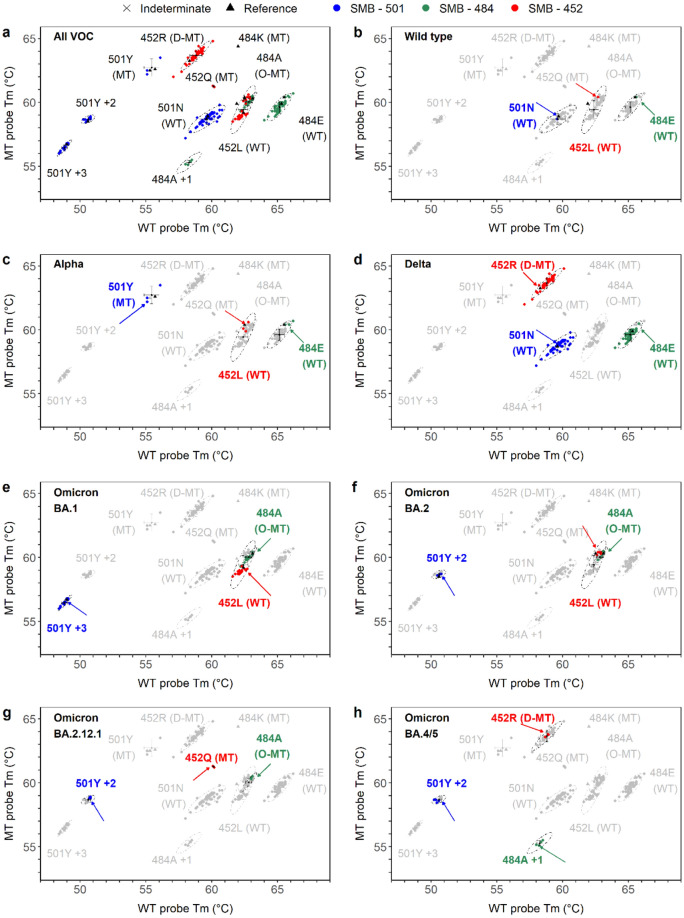
Variant of concern (VOC) detection using the SARS-COV-2 SMB-VOC assay. Correlation plots showing grouping and classification of patient specimens (N = 84) tested in Roche LC480 instrument using SMB-501 (Blue), SMB-484 (Green) and SMB-452 (Red) assays (**a**). Identification Tm signature specific for the wild type (WT, B); Alpha (**c**); Delta (**d**); Omicron BA.1 (**e**); Omicron BA.2 (**f**); Omicron BA.2.12.1 (**g**) and Omicron BA.4/5 (**h**) are highlighted and indicated by arrows. 501Y+2 and 501Y+3 indicates additional mutations on the SMB-501 binding region. 484A+1 indicates additional mutations on the SMB-484 binding region. D-MT indicates delta defining mutation; O-MT indicates omicron specific mutation for SMB-484 assay component.

As shown in Table 3S, a total of 90 confirmed COVID-19 positive patient specimens and 9 confirmed CoV-2 negative specimens were tested in a Roche LightCycler 480 to evaluate the clinical performance of the SMB-VOC assay. The sample source, collection timeline and the initial Ct value at collection was also recorded (Table 3S). We tested specimens collected over 17 months from April 2021 to September 2022, with Ct values that ranged from 12 to 37.6 (Mean Ct of 24 ± 5). All VOCs were identified as described in methods using the Excel analyze tool to transform Tm values into variant identification (supplementary file Appendix 1). The Tm values obtained from all 3 SMB assay components and identification for each patient sample tested are listed in Tables 2 and 3S. The clustering of various mutations and the establishment of a Tm signature to identify specific WT or VOCs using our assay (SMB-VOC assay), is demonstrated in Fig. 2. Sanger sequencing of all specimens (Tables 2 and 4S) was used to confirm the target codon sequence (either WT or mutant) in each specimen. Compared to Sanger sequencing, the overall assay performed in an LC480 instrument correctly identified 51/56 (91%) of the Delta variants as Delta with the remaining 5/56 Delta variants as indeterminates. The assay also correctly identified all 16/16 (100%) Omicron BA.1 variants as Omicron BA.1, all 6/6 (100%) Omicron BA.2 variants as BA.2, all 3/3 (100%) Omicron BA.2.12.1 variants as Omicron BA.2.12.1, and all 3/3 (100%) Omicron BA.4/5 variants as Omicron BA.4/5. We have set the SMB-VOC assay results call with high stringency, so any specimen failing to yield a Tm in > 2 probes or producing a Tm that falls outside of a defined Tm window will result in an indeterminate call. The five specimens (VSAP36, VSAP38, VSAP39, VSAP43, VSAP79) that produced indeterminate results (as described above) by the SMB-VOC assay (Table 3S) were identified as Delta by Sanger/whole genome sequencing (Table 4S). Sanger sequencing also failed in 4 specimens that the SMB-VOC assay detected (Table 4S). None of the specimens were incorrectly identified as WT or incorrectly identified as a different VOC using the Excel Analysis tool (Supplementary file Appendix 1) for VOC identification. Thus, excluding specimens with indeterminant results, the SMB-VOC assay was 100% sensitive and 100% specific compared to sequencing. Including the five indeterminate specimens the SMB-VOC assay sensitivity was reduced to 94.3% (81/86), without changing the specificity (Table 3).

**Table 3:** Sensitivity and specificity of SMB-VOC assay compared to sequencing.

		Sequencing									Combined % Sensitivity
	CoV-2 Variants	WT	Alpha	Delta	Omicron BA.1	Omicron BA.2	Omicron BA.2.12.1	Omicron BA.4/5	ND	Neg	
SMB-VOC assay	WT	1	0	0	0	0	0	0	0	0	
	Alpha	0	3	0	0	0	0	0	0	0	
	Delta	0	0	47	0	0	0	0	0	0	
	Omicron BA.1	0	0	0	16	0	0	0	0	0	
	Omicron BA.2	0	0	0	0	6	0	0	0	0	
	Omicron BA.2.12.1	0	0	0	0	0	3	0	0	0	
	Omicron BA.4/5	0	0	0	0	0	0	3	1	0	
	Indeterminates	0	0	5	0	0	0	0	0	0	
	ND	0	0	0	0	0	0	0	1	0	
	Neg	0	0	0	0	0	0	0	0	9	
% Sensitivity		100	100	91	100	100	100	100	100		94.3
Combined %Specificity		100									

Sensitivity = TP/(TP + FN) and Specificity = TN/ (FP + TN), where TP, True positives; TN, True negatives; FN, False negatives. WT- SARS-CoV-2 USA-WA1/2020. ND-Not done; Neg-Negatives.

One of the patient specimens (VSAP79), showed 2 melt peaks in the SMB-484 assay with repeated testing and was also classified as “variant indeterminate”. The region surrounding codon 484 of this unusual sample was Sanger sequenced, showing a WT 484 codon. However, both strands returned a Y (C/T) nucleotide at the 483-codon position (GTT-GYT), where the WT sequence would have a GTT (V) and the mutant is a GCT (A). With the 484-WT probe, a mutant at this position or a mixture of strains can cause a shift in the Tm, thus yielding an additional peak. The V483A mutation has been reported in long term SARS-CoV-2 infections^36^. This sample; however, was WT with SMB-501 and mutant (452R) with SMB-452 assay components. To the best of our knowledge, none of the known circulating strains of SARS-CoV-2 is reported to contain both 483A and 452R mutations. Thus, we supposed that this sample might be mixed with delta strain RNA and a sequence variant not included in our Tm code reference library. Sample cross contamination could also not be ruled out. Upon whole genome sequencing, the strain was identified as Delta subvariant AY.25.1.

### Assay adaptability in different RT-PCR instruments

To further understand the adaptability of our assay in diagnostic and hospital laboratories, we validated the method in three additional commonly used RT-PCR instruments with melt capability. The performance of these new test instruments was compared to the LC480 as the gold standard. A total of 34 out of the 90 COVID-19 positive patient specimens were tested with all three defined assays (SMB-501, SMB-484, and SMB-452) on different RT-PCR instruments (Supplementary Table 2S). The BioRadCFX96 identified 97% (33/34) of the specimens correctly and one sample tested negative (Kappa, k  = 0.9). The ABI7500 identified 32/34 (94%) of the specimens correctly (k  = 0.9), and one sample was negative and the other was indeterminate. However, the Qiagen rotor-geneQ (RGQ) instrument performed relatively poorly, where it identified 67.6% (23/34) of the specimens correctly (k  = 0.39) and 11/34 (32.4%) of the specimens were indeterminates. Thus, the SMB-VOC assay is highly adaptable and reproducible in most RT-PCR instruments with melt capability.

## Discussion

Although whole genome sequencing is a powerful tool to identify new viral lineages, a simple high throughput screening test that accurately identifies VOC provides many advantages. The current study demonstrates that a modification of our simple and easily adoptable assay which was previously developed to identify Alpha, Beta and Gamma strains^30^, can also detect Delta and the rapidly emerging Omicron subvariants. In our previous publication^30^, we hypothesized that mutations at the codons 501 and 484 would be common in other emergent SARS-CoV-2 variants, and mutations at these codons are repeated in Omicron. Although Delta was predominantly wild type at codons 501 and 484, mutations at codon 452 similar to the CAL.20C variant observed first in California^41^, are considered a key Delta-defining mutation. This codon has also been a hotspot for emerging VOCs, such as Omicron BA.2.12.1 and Omicron BA.4/5. The Kraken subvariant (XBB.1.5) could also be distinguishable from other Omicron subvariants since it is WT on the 452 codon but has an additional mutation on 484 codon. The mutations on these codons have been shown to be responsible for the increased infectivity, transmission, escape humoral immunity and reduced susceptibility to monoclonal antibody treatments^13, 42, 43^ and resistance to antivirals^44, 45^.

Our assay offers unique advantages over other melting temperature based assays^37–40^. It adopts an unique Tm signature with a combination of 6 different probes targeting the 3 key spike protein codons (452, 484 and 501). The assay is sensitive, specific and can be performed in a multi-well plate format for high throughput testing in most qRT-PCR instruments once reference Tm values are established, unlike almost all commercial assays^39, 46, 47^ and other published methods^37, 39, 40, 48, 49^. Certain instruments such as Qiagen Rotagene-Q as shown in this study may require further optimization. The excel analysis tool offers an advantage of easy SARS-CoV-2 VOC identification which we have not found in other studies. Additionally, the database of Tm values used for VOC identification can be updated with reference Tms as new variants emerge, thus increasing the strength of the output result.

The assay proved to be highly specific, however yielded six indeterminates, including the sample VSAP17 (initial PCR Ct = 37.6), which could be due to sample degradation or SARS- CoV-2 viral loads below the probe LoD (Ct > 35). On the other hand, our SMB-VOC assay also detected correct VOC type for three samples that failed sequencing. The rapidly changing strain variants in the ongoing pandemic complicated efforts to statistically power and perform an evaluation study which includes all relevant VOCs for every subvariant of Omicron, during the study period (late November 2021 through September 2022). However, to address this limitation, we have supplemented our clinical study with defined reference variants obtained from BEI resources. Future clinical studies performed at multiple sites over longer periods would be able to further enlarge our database of different VOCs and their corresponding Tm codes.

In conclusion, our SMB-VOC assay can help detect and differentiate SARS-CoV-2 variants at the key mutation sites, which can offer rapid, highthroughput and cost effective alternative to sequencing. Future studies will focus on integrating our result calling algorithm with relevant testing systems to further simplify rapid VOC identification on commonly used instruments. Assays such as ours that detect mutations in these key codons will aid in VOC surveillance and may also help guide targeted therapy.

## Methods

### Ethical considerations

The usage of de-identified banked clinical specimens from RT-PCR confirmed COVID-19 positive and negative patients in this study was approved by the Rutgers University institutional Review Board under protocol numbers 20170001218 and 2020001541. All methods were performed in accordance with the relevant guidelines and regulations.

### Viral cultures and RNA

Reference SARS-CoV-2 RNA or viral cultures listed in Table 2 were obtained from BEI Resources, NIAID (Manassas, VA). RNA was isolated from the variant strains in a BSL3 laboratory using RNAdvance viral RNA extraction kit (Beckman Coulter, Indianapolis, IN). The extracted RNA was quantified using Roche LC480 or digital PCR using the primers specific for N1 gene^35^.

### Assay design, primers and probes

SMBs and primers verified to detect mutations in codon N501 and E484 were used as reported earlier^30^. An additional assay to detect the L452R (T22917G) mutation present in B.1.617.2 (Delta) was designed in a similar manner as described previously. Briefly, a total of 412,389 high quality SARS-CoV-2 genome sequences deposited in GISAID as of Feb 19, 2021, were analyzed using BLAST and aligned with MAFFT^50^. Primers and probes were designed on the basis of sequence conservation using Primer3^51^ to amplify a 122 bp region flanking the position 22917 (452 codon) in the SARS-CoV-2 reference strain (GenBank accession number MN908947). SMB probe design was performed using the web server DNA mfold (http://www.unafold.org/mfold/applications/dna-folding-form.php) and DINAmelt (http://www.unafold.org/hybrid2.php) to predict the probe folding structures and probe-target hybrid Tm values respectively. Similar genome analysis was performed for the Omicron subvariant strains comparing them to other variant strains at the primer/probe binding regions. A total of 4,048 high quality sequences were analyzed for position 484 mutations and 3,964 high quality sequences for position 501 mutations. All analyzed genomes were wildtype at position 452. In-silico two state melting hybridizations were performed to understand the Tm variations using the DINAmelt application. The final list of primers and probes used in this study are listed in Table 1S. Due to the presence of mutations in the primer binding region of the Omicron variant and subvariants, wobble nucleotides were introduced in the originally published 484 primer pair^30^. Primers were obtained from Millipore Sigma (St. Louis, MO) and Integrated DNA Technologies (IDT) (Coralville, IA), and SMBs were synthesized by LGC Biosearch technologies (Petaluma, CA). An internal control (IC) assay developed by CDC^52, 53^, targeting the human RNaseP gene was simultaneously performed for each extracted RNA specimen as a separate reaction in a separate well, using the *Taq*Man real-time PCR assay probe tagged with FAM at the 5’ end and Dabcyl quencher at the 3′ end.

### SMB-assay formulation

TaqPath 1-Step RT-qPCR Master Mix, CG (ThermoFisher Scientific, Waltham, MA) was supplemented with the assay primers and probes at final concentrations as mentioned in Table 1 for an asymmetric one-step RT-PCR. A 1 µl of the template RNA was added per 20 µl reaction.

### Analytical sensitivity

The pre-quantitated genomic RNA from the SARS-CoV-2 B.1.617.2 (Delta) and B.1.1.529 (Omicron BA.1 and BA.2) were diluted in Tris–EDTA (TE) buffer. For the background matrix, total nucleic acids were extracted from a SARS-CoV-2 negative nasopharyngeal (NP) specimen (confirmed negative by the Xpert Xpress SARS-CoV-2 test). Reference RNA from Delta/Omicron BA.1/Omicron BA.2 were spiked into the negative matrix. Delta RNA was spiked at final concentrations of 200, 100, 20, 2 and 0.2 GE/reaction, and Omicron RNA (BA.1/BA.2) was spiked at final concentrations ranging from 10^3^ to 1 GE/reaction. Each dilution was tested in replicates of 8. A 1 µl aliquot of this mix was added to 19 µl of the one-step RT-PCR mix containing the primers and probes and was evaluated in the SMB-501/SMB-484/SMB-452 assays. The LOD was defined at 95% positive rate on the non-linear regression fit curve analysis (Fig. 1). A standard curve was generated using the US CDC N1 assay^35^, in Roche LC480 to establish corresponding Ct values.

### Excel analysis tool for identification of SARS-CoV-2 variants

For identification and classification of the VOCs, we modified a Microsoft Excel based program originally published by Chakravorty et al.^54^. Briefly, the program finds the closest match between the Tm signature from the patient specimens to that of the reference VOCs. A distance index (D-value) is calculated based on the difference in values between the reference and the unknown. A D-value of < 5 was considered in these studies as a perfect match and ≥ 5 was classified as “indeterminate”. The program uses the Ct value of the internal control (IC) to assess failed versus successful runs. An “invalid” call is made if the IC fails to generate a Ct along with the SMB probes and a “SARS-CoV-2 Not Detected” call is made if the N1 gene fails to generate a Ct. For VOC classification, the Tm signature values that are generated from each of the six SMB probes (two SMB for each of the three codons) are entered and the tool generates an output result of either ‘Variant Indeterminate’/‘Wild type (Ancestral)’/‘B.1.1.7 (Alpha)’/‘B. 1.351 (Beta)’/‘B.1.617.2 (Delta)’/‘B.1.1.529 (Omicron BA.1)’/‘B.1.1.529 (Omicron BA.2)’/‘B.1.1.529 (Omicron BA.2.12.1)’/ or ‘B.1.1.529 (Omicron BA.4/5)’. A ‘Variant Indeterminate’ output is obtained if the Tm values are outside of the reference window (as described above) or if a Tm value of zero is entered for > 2 SMB probes due to the failure of these probes to generate a Tm. However, if only 1 or 2 of the SMB probes fail to generate a Tm, the tool matches the remaining Tm values to the closest reference and reports the identified VOC as ‘presumptive’. This program file is attached as supplementary file Appendix 1.

### Assay protocol

All assays were run as separate reactions as a 4-well test including the internal control in 384-well plates in a Roche LightCycler 480 (Roche, Indianapolis, IN). The one-step RT-PCR amplification was performed with same PCR conditions described previously^30^, and mentioned in Table 1. The total assay time was 1 h 17 min. Tm values obtained from the instrument for each SMB-assay from both WT and MT probes, were exported and identified using the Excel analyze tool (supplementary file Appendix 1).

### Clinical specimen evaluation and RT-PCR instrument feasibility

A total of 90 banked specimens containing deidentified nasopharyngeal (NP) swabs, nasal swabs, and saliva obtained from patients undergoing routine COVID-19 clinical testing at the CLIA and CAP certified laboratories at the Public Health Research Institute (PHRI) and University Hospital, Newark, NJ, were selected for this study. RT-PCR cycle threshold (Ct) values at collection, ranged from a minimum Ct of 12.4 to a maximum Ct of 37.6 and were collected from the months of April 2021 through September 2022. All specimens were tested in a Roche LightCycler 480 (LC480, Roche, Indianapolis, IN). Additionally, the first thirty-four of these specimens were used for testing in various RT-PCR instruments with all 3 assays to establish a proof of concept on the applicability of the assay in other RT-PCR instruments. The RT-PCR instruments used were a, a Bio-Rad CFX96 (Bio-Rad, Hercules, California), Applied Biosystems7500 (Thermo Fisher Scientific), and a Rotor Gene Q (Qiagen, Germantown, MD) located in PHRI laboratories, NJMS genomic laboratory and the UH molecular diagnostics laboratory. These instruments were selected based on the availability for testing with our assay. The distribution of the number of specimens collected over the months and the range of Ct values are shown in supplementary Fig. 1S. RNA was extracted from all the specimens using a QIAamp viral RNA isolation kit (Qiagen) or a QIASymphony DSP viral RNA extraction kit in a QIAsymphony automated instrument (Qiagen) according to the manufacturers recommendations, and a 5 µl volume of this extracted RNA was added to the one-step RT-PCR mix containing the primers and probes. Each specimen was run with all 3 assays (SMB-501, SMB-484, and SMB-452) in separate wells. All instruments were programmed with a protocol similar to that used in the LC480 as mentioned in the Table 1S. The internal control targeting RNaseP was run for all specimens. A reference Tm code was established for each SMB assay on all platforms using the WT genomic RNA, Alpha, Beta, Delta, and Omicron BA.1 RNA. Specimens that tested positive for SARS-CoV-2 wildtype or a VOC were confirmed by Sanger sequencing at the Department of Genomic Medicine, Rutgers Biomedical and Health Sciences, Newark using the primer pair: F-5′aggctgcgttatagcttgga3′ and R-5′aaacagttgctggtgcatgt3′ which amplifies a 284 bp segment of the S-gene inclusive of the amino acid positions at 452, 484, and 501. Sequencing data were analyzed using Ugene (ver 37)^55–57^ or MegAlign Pro software (DNAStar, ver16). Seven representative clinical specimens (VSAP75, VSAP76, VSAP79, VSAP80, VSAP84, VSAP85, and VSAP90) were submitted for whole genome sequencing to the Department of Genomic Medicine, Rutgers Biomedical and Health Sciences, Newark to confirm the identification of Omicron subvariants, and a Delta subvariant sample. The sample library was prepared using the Qiagen QIAseq Direct SARS-CoV-2 kit (Qiagen, Cat #333891, Germantown, MD). Random-primed cDNA synthesis was performed on the viral RNA, followed by high-fidelity multiplex PCR. The 250 bp enriched amplicon pools were amplified and indexed with unique dual indices. The sequencing was run on the Illumina Miniseq nextGeneration sequencer using a 300-cycle kit and analyzed using the SARS-CoV-2 workflow in the QIAGEN CLC Genomics Workbench program. The FASTA files were used to create a phylogenic tree in Nextclade CLI 2.5.0, Nextclade Web 2.5.0.

### Statistical analysis

Standard statistical analyses (average, standard deviation) and graphing were performed using Microsoft excel (ver 2102), GraphPad Prism 8.4.3 for Windows, R version 4.1.1 and ggplot2 package.

### Supplementary Information

Supplementary Information 1.

Supplementary Information 2.

Supplementary Information 3.

Supplementary Information 4.

## Data Availability

All the data generated for this study is presented in the manuscript and as supplementary material. The datasets generated and/or analyzed during the current study are available in the in NCBI sequence read archive (SRA) repository under the NCBI Bioproject PRJNA946757. Accession numbers of deposited sequences are provided as supplementary material. Any additional data used in the current study will be made available upon reasonable request to the corresponding author.

## References

[1] CDC. *SARS-CoV-2 B.1.1.529 (Omicron) Variant—United States, December 1–8, 2021* 1731–1734 (2021). 10.15585/mmwr.mm7050e1 PMC8675659 34914670

[2] CDC. *Omicron Variant: What You Need to Know*. https://stacks.cdc.gov/view/cdc/112335 (2022).

[3] Hodcroft, E. *CoVariants: SARS-CoV-2 Mutations and Variants of Interest. *https://covariants.org/ (2022).

[4] Ling Y (2022). The Omicron BA.2.2.1 subvariant drove the wave of SARS-CoV-2 outbreak in Shanghai during spring 2022. Cell Discov..

[5] Mohapatra RK (2022). The recently emerged BA.4 and BA.5 lineages of Omicron and their global health concerns amid the ongoing wave of COVID-19 pandemic—correspondence. Int. J. Surg..

[6] Ochoa-Hein E (2022). Significant rise in SARS-CoV-2 reinfection rate in vaccinated hospital workers during the omicron wave: A prospective cohort study. Rev. Invest. Clin..

[7] Prevention, C. C. F. D. C. A. *Omicron variant: What you need to know*. https://stacks.cdc.gov/view/cdc/112335 (2021).

[8] WHO. *Tracking SARS-CoV-2 variants*. https://www.who.int/en/activities/tracking-SARS-CoV-2-variants (2021).

[9] O'Toole, Á., Scher, E. & Rambaut, A. *SARS-CoV-2 lineages*. https://cov-lineages.org/lineage_list.html (2022).

[10] CDC. *COVID Data Tracker*. https://covid.cdc.gov/covid-data-tracker/#variant-proportions (2023).

[11] CDC. *Variants and Genomic Surveillance for SARS-CoV-2*. https://www.cdc.gov/coronavirus/2019-ncov/variants/genomic-surveillance.html (2021).

[12] Lacobucci G (2021). Covid-19: New UK variant may be linked to increased death rate, early data indicate. BMJ.

[13] Santos JC, Passos GA (2021). The high infectivity of SARS-CoV-2 B.1.1.7 is associated with increased interaction force between Spike-ACE2 caused by the viral N501Y mutation. BioRxiv.

[14] Brown CM, Johnson V, Johnson H (2021). Outbreak of SARS-CoV-2 infections, including COVID-19 vaccine breakthrough infections, associated with large public gatherings—Barnstable County, Massachusetts, July 2021. MMWR Morb. Mortal Wkly. Rep..

[15] Singanayagam A (2022). Community transmission and viral load kinetics of the SARS-CoV-2 delta (B.1.617.2) variant in vaccinated and unvaccinated individuals in the UK: A prospective, longitudinal, cohort study. Lancet Infect. Dis..

[16] Callaway E, Mallapaty S (2021). Novavax offers first evidence that COVID vaccines protect people against variants. Nature.

[17] Greaney AJ (2021). Comprehensive mapping of mutations in the SARS-CoV-2 receptor-binding domain that affect recognition by polyclonal human plasma antibodies. Cell Host Microbe.

[18] Vitiello A, Ferrara F, Auti AM, Di Domenico M, Boccellino M (2022). Advances in the Omicron variant development. J. Intern. Med..

[19] Wang Q (2022). Antibody evasion by SARS-CoV-2 Omicron subvariants BA.2.12.1, BA.4 and BA.5. Nature.

[20] Wu K (2021). mRNA-1273 vaccine induces neutralizing antibodies against spike mutants from global SARS-CoV-2 variants. BioRxiv.

[21] Lauring AS, Hodcroft EB (2021). Genetic variants of SARS-CoV-2—what do they mean?. JAMA.

[22] Rubin R (2021). COVID-19 vaccines vs variants—determining how much immunity is enough. JAMA.

[23] Tchesnokova V (2021). Acquisition of the L452R mutation in the ACE2-binding interface of spike protein triggers recent massive expansion of SARS-CoV-2 variants. J. Clin. Microbiol..

[24] Tian D, Sun Y, Zhou J, Ye Q (2021). The global epidemic of the SARS-CoV-2 delta variant, key spike mutations and immune escape. Front. Immunol..

[25] Weisblum Y (2020). Escape from neutralizing antibodies by SARS-CoV-2 spike protein variants. Elife.

[26] Xia S, Wang L, Zhu Y, Lu L, Jiang S (2022). Origin, virological features, immune evasion and intervention of SARS-CoV-2 Omicron sublineages. Sig. Transduct. Target Ther..

[27] Harankhedkar S (2022). N Gene Target Failure (NGTF) for detection of Omicron: A way out for the ‘stealth’ too?. MedRxiv.

[28] Mills MG (2022). Rapid and accurate identification of SARS-CoV-2 Omicron variants using droplet digital PCR (RT-ddPCR). J. Clin. Virol..

[29] Dikdan RJ (2022). Multiplex PCR assays for identifying all major severe acute respiratory syndrome coronavirus 2 variants. J. Mol. Diagn..

[30] Banada P (2021). A simple reverse transcriptase PCR melting-temperature assay to rapidly screen for widely circulating SARS-CoV-2 variants. J. Clin. Microbiol..

[31] Golubchik T (2021). Early analysis of a potential link between viral load and the N501Y mutation in the SARS-COV-2 spike protein. MedRxiv.

[32] Leung K, Shum MH, Leung GM, Lam TT, Wu JT (2021). Early transmissibility assessment of the N501Y mutant strains of SARS-CoV-2 in the United Kingdom, October to November 2020. Eurosurveillance.

[33] Cao Y (2021). Xpert MTB/XDR: A 10-color reflex assay suitable for point-of-care settings to detect isoniazid, fluoroquinolone, and second-line-injectable-drug resistance directly from mycobacterium tuberculosis-positive sputum. J. Clin. Microbiol..

[34] Chakravorty S (2017). The new Xpert MTB/RIF Ultra: Improving detection of mycobacterium tuberculosis and resistance to rifampin in an assay suitable for point-of-care testing. mBio.

[35] Lu X (2020). US CDC real-time reverse transcription PCR panel for detection of severe acute respiratory syndrome coronavirus 2. Emerg. Infect. Dis..

[36] Ashwaq O, Manickavasagam P, Haque SM (2021). V483A: An emerging mutation hotspot of SARS-CoV-2. Future Virol..

[37] Sun L (2023). Rapid detection of predominant SARS-CoV-2 variants using multiplex high-resolution melting analysis. Microbiol. Spectr..

[38] Erster O (2021). Rapid and high-throughput reverse transcriptase quantitative PCR (RT-qPCR) assay for identification and differentiation between SARS-CoV-2 variants B.1.1.7 and B1.351. Microbiol. Spectr..

[39] Berno G (2022). SARS-CoV-2 variants identification: Overview of molecular existing methods. Pathogens.

[40] Kong X (2023). Discrimination of SARS-CoV-2 omicron variant and its lineages by rapid detection of immune-escape mutations in spike protein RBD using asymmetric PCR-based melting curve analysis. Virol. J..

[41] Zhang W (2021). Emergence of a novel SARS-CoV-2 variant in Southern California. JAMA.

[42] Imai M (2022). Efficacy of antiviral agents against omicron subvariants BQ.1.1 and XBB. N. Engl. J. Med..

[43] Tao K (2021). The biological and clinical significance of emerging SARS-CoV-2 variants. Nat. Rev. Genet..

[44] Sjaarda CP (2023). Prevalence of low-frequency, antiviral resistance variants in SARS-CoV-2 isolates in Ontario, Canada, 2020–2023. JAMA Netw. Open.

[45] Iketani S (2023). Multiple pathways for SARS-CoV-2 resistance to nirmatrelvir. Nature.

[46] Neopane P, Nypaver J, Shrestha R, Beqaj SS (2021). SARS-CoV-2 variants detection using TaqMan SARS-CoV-2 mutation panel molecular genotyping assays. Infect. Drug Resist..

[47] Vogels CBF (2021). Multiplex qPCR discriminates variants of concern to enhance global surveillance of SARS-CoV-2. PLoS Biol..

[48] Dakhave M, Gadekar S, Malekar A, Wankhede G (2023). 'CoviSwift(TM'): A point-of-care RT-PCR device for SARS-CoV-2 and its variant detection. J. Virol. Methods.

[49] Kumar M (2021). FnCas9-based CRISPR diagnostic for rapid and accurate detection of major SARS-CoV-2 variants on a paper strip. Elife.

[50] Katoh K, Misawa K, Kuma K, Miyata T (2002). MAFFT: A novel method for rapid multiple sequence alignment based on fast Fourier transform. Nucleic Acids Res..

[51] Untergasser A (2012). Primer3–new capabilities and interfaces. Nucleic Acids Res..

[52] Emery SL (2004). Real-time reverse transcription-polymerase chain reaction assay for SARS-associated coronavirus. Emerg. Infect. Dis..

[53] Wyllie AL (2020). Saliva or nasopharyngeal swab specimens for detection of SARS-CoV-2. N. Engl. J. Med..

[54] Chakravorty S (2010). Rapid universal identification of bacterial pathogens from clinical cultures by using a novel sloppy molecular beacon melting temperature signature technique. J. Clin. Microbiol..

[55] Golosova O (2014). Unipro UGENE NGS pipelines and components for variant calling, RNA-seq and ChIP-seq data analyses. PeerJ.

[56] Okonechnikov K, Golosova O, Fursov M (2012). Unipro UGENE: A unified bioinformatics toolkit. Bioinformatics.

[57] Rose R, Golosova O, Sukhomlinov D, Tiunov A, Prosperi M (2019). Flexible design of multiple metagenomics classification pipelines with UGENE. Bioinformatics.

