# Supplementary material for: An expanded RT-PCR melting temperature coding assay to rapidly identify all known SARS-CoV-2 variants and sub-variants of concern
Source: Sci Rep. 2023 Dec 11;13:21927. doi: 10.1038/s41598-023-48647-8 (PMC10713575; doi:10.1038/s41598-023-48647-8)
Supplement: Supplementary file 2 — Supplementary Information 2. [file 41598_2023_48647_MOESM2_ESM.pdf]

We gratefully acknowledge the following Authors from the Originating laboratories responsible for obtaining the specimens, as well as the Submitting laboratories where the genome data were generated and shared via GISAID, on which this research is based.

All Submitters of data may be contacted directly via www.gisaid.org

Authors are sorted alphabetically.

Accession ID	Originating Laboratory	Submitting Laboratory	Authors
EPI_ISL_7543837, EPI_ISL_7543885, EPI_ISL_7544121, EPI_ISL_7544319, EPI_ISL_7544331, EPI_ISL_7544369, EPI_ISL_7544456, EPI_ISL_7544613, EPI_ISL_7544642, EPI_ISL_7544715			
see above	2 Military Hospital wc MAA	NHLS/UCT	Arash Iranzadeh; Bruna Galvao; Carolyn Williamson; Deelan Doolabh; Diana Hardie; Gert Marais; Innocent Mudau; Luicer Olubayo; Lynn Tyers; Marvin Hsiao; Nokuzola Mbhele; Rageema Joseph; Stephen Korsman
EPI_ISL_7621467, EPI_ISL_7621483, EPI_ISL_7621936	4Cyte Pathology	NSW Health Pathology - Institute of Clinical Pathology and Medical Research; Westmead Hospital; University of Sydney	Arnott A.; Draper J.; Gall M.; Martinez E.; Rockett R.; Sintchenko V.; on behalf of ICPMR
EPI_ISL_7495248, EPI_ISL_7495249, EPI_ISL_7495250	A. Krumbholz, Labor Dr. Krause und Kollegen MVZ GmbH, Kiel	Charité Universitätsmedizin Berlin, Institut für Virologie	Barbara Mühlemann; Christian Drosten; Julia Schneider; Julia Tesch; Jörn Beheim-Schwarzbach; Talitha Veith; Terry Jones; Tobias Bleicker; Victor M Corman
EPI_ISL_7405721	A.S.L. CITTA DI TORINO - OSPEDALE AMEDEO DI SAVOIA	Fondazione del Piemonte per l'Oncologia IRCCS	Antonino Sottile; Giorgio Giardina; Paola Marino; Silvia Brossa
EPI_ISL_7306737	A.S.L. TO4	Fondazione del Piemonte per l'Oncologia IRCCS	Antonino Sottile; Giorgio Giardina; Paola Marino; Silvia Brossa
EPI_ISL_7016910, EPI_ISL_7569944, EPI_ISL_7569960, EPI_ISL_7569961, EPI_ISL_7569968, EPI_ISL_7569970, EPI_ISL_7569978, EPI_ISL_7569980, EPI_ISL_7569985, EPI_ISL_7569988			
see above	ACT Pathology	Schwessinger Lab	Ashley Jones; Austin Bird; Bayantes Dagvadorj; Benjamin Schwessinger; Carl McCombe; Carolina Correa Ospina; Catalina Barragán Quintero; Craig Kennedy; Elise Kellett; Emma Crean; Evie Hodgson; Gabrielle Smith; Karina Kennedy; Rachel Leonard; Rene Riedelbauch; Robyn Hall; Salome Wilson; Scott Ferguson
EPI_ISL_6891760, EPI_ISL_7129657, EPI_ISL_7129884, EPI_ISL_7130085, EPI_ISL_7130189, EPI_ISL_7130344, EPI_ISL_7130486			
see above	AGES-Institute for medical Microbiology and Hygiene Vienna	AGES-Institute for medical Microbiology and Hygiene Vienna	Alexander Indra; Elisabeth Polster; Florian Heger; Julia Klikovits; Kathrin Lippert; Marion Blaschitz; Patrick Hyden; Peter Hufnagl; Stefanie Dobrovolny; Vera Wallner
EPI_ISL_7381191	AHRI	CERI, Centre for Epidemic Response and Innvoation, Stellenbosch University and KRISP, KZN Research Innovation and Sequencing Platform, UKZN.	Arisha Maharaj; Bernstein Mallory; Cele Sandile; Giandhari J; Karim Farina; Khan Khadija; Moir M; Naidoo Y; Pillay S; Ramphal U; Ramphal Y; San JE; Sigal Alex; Tegally H; Tshiabuila D; Wilkinson E; de Oliveira T; van Wyk S
EPI_ISL_7358094	AHRI-Sigal	CERI, Centre for Epidemic Response and Innvoation, Stellenbosch University and KRISP, KZN Research Innovation and Sequencing Platform, UKZN.	Bernstein Mallory; Cele Sandile; Giandhari J; Karim Farina; Khan Khadija; Moir M; Naidoo Y; Nokukhanya Mdlalose; Pillay S; Ramphal U; Ramphal Y; San JE; Sigal Alex; Tegally H; Tshiabuila D; Wilkinson E; de Oliveira T
EPI_ISL_7644368	AOPD	Istituto Zooprofilattico Sperimentale delle Venezie	Adelaide Milani; Alessia Schivo; Alice Fusaro; Ambra Pastori; Angela Salomoni; Annalisa Salviato; Antonia Ricci; Calogero Terregino; Edoardo Giussani; Elisa Palumbo; Erika Giorgia Quaranta; Isabella Monne
EPI_ISL_7637189, EPI_ISL_7637190	AULSS 3 Venezia	UOSD Genetica e Citogenetica - Azienda ULSS 3 Serenissima; Istituto Zooprofilattico Sperimentale delle Venezie	Adelaide Milani; Alessia Schivo; Alice Fusaro; Ambra Pastori; Angela Salomoni; Annalisa Salviato; Antonia Ricci; Calogero Terregino; Edoardo Giussani; Elisa Palumbo; Elisa Squarcina; Erika Giorgia Quaranta; Isabella Monne; Laura Bevilacqua; Laura Squarzon; Luca Sorino; Mosé Favarato; Noemi Laganà
EPI_ISL_7638072	AULSS 8 Berica	Istituto Zooprofilattico Sperimentale delle Venezie	Adelaide Milani; Alessia Schivo; Alice Fusaro; Ambra Pastori; Angela Salomoni; Annalisa Salviato; Antonia Ricci; Calogero Terregino; Edoardo Giussani; Elisa Palumbo; Erika Giorgia Quaranta; Isabella Monne
EPI_ISL_7636072	AZDelta	AZ Delta Medical Laboratories in Roeselare, Belgium	Dieter De Smet; Frederik Van Hoecke; Geert Martens; on behalf of AZ Delta COVID-19 Genomics core (member of Genomic surveillance of SARS-CoV-2 in Belgium network)
EPI_ISL_7660967, EPI_ISL_7660969	Accelerated Clinical Laboratories	City of Milwaukee Health Department Laboratory	Amy Bauer; Manjeet Khubbar; Samantha Scott; Sanjib Bhattacharyya
EPI_ISL_7154405	Aegis Sciences Corporation	Centers for Disease Control and Prevention Division of Viral Diseases, Pathogen Discovery	Alec Vest; Benjamin Rambo-Martin; Christopher Gulvick; Clinton Paden; Cyndi Clark; Dakota Howard; Dhwani Batra; Dillon Nall; Duncan MacCannell; Erisa Sula; Ethan Sanders; Holly Houdeshell; Jason Caravas; Kristine Lacek; Matthew Hardison; Matthew Schmerer; Ola Kvalvaag; Patrick Campbell; Peter Cook; Rob Case; Scott Sammons; Shatavia Morrison; Shaun Westlund; Tymeckia Kendall; Victoria Caban Figueroa; Vikramsinha Ghorpade; Yvette Unoarumhi
EPI_ISL_7452769, EPI_ISL_7452775	Aesculabor Hamburg, Institut der Labormedizin	Heinrich Pette Institute, Leibniz Institute for Experimental Virology	Adam Grundhoff; Alexis Robitaille; Johannes Knobloch; Martin Aepfelbacher; Nicole Fischer; Thomas Günther
EPI_ISL_7470217, EPI_ISL_7470278, EPI_ISL_7470360, EPI_ISL_7470368, EPI_ISL_7470391, EPI_ISL_7470446	Akershus University Hospital, Department for Microbiology and Infectious Disease Control	Norwegian Institute of Public Health, Department of Virology	Atiya R Ali; Debech Nadia; Engebretsen Serina Beate; Garcia Llorente Ignacio; Hilde Elshaug; Hilde Vollan; Jon Bråte; Kamilla Heddeland Instefjord; Karoline Bragstad; Kathrine Stene-Johansen; Line Victoria Moen; Marie Paulsen Madsen; Olav Hungnes; Pedersen Benedikte Nevjen; Rasmus Riis Kopperud
EPI_ISL_7620089	Alaska State Virology Laboratory	Alaska State Virology Laboratory	Elva House; Jack Chen; Jacob Zidek; Jeremy Roe; Lisa Smith; Ph.D.
EPI_ISL_7146436, EPI_ISL_7506705	Allergy, Immunology and Cell Biology Unit (AICBU)	Allergy, Immunology and Cell Biology Unit (AICBU)	Ayesha Wijesinghe; Chandima Jeewandara; Deshni Jayathilaka; Dinuka Ariyaratne; Diyanath Ranasinghe; Dumni Guasinghe; Farha Bary; Gathsaurie Neelika Malavige; Tibutius Thanesh
EPI_ISL_7544441	Alma CDC wc AHC	NHLS/UCT	Arash Iranzadeh; Bruna Galvao; Carolyn Williamson; Deelan Doolabh; Diana Hardie; Gert Marais; Innocent Mudau; Luicer Olubayo; Lynn Tyers; Marvin Hsiao; Nokuzola Mbhele; Rageema Joseph; Stephen Korsman
EPI_ISL_7264139, EPI_ISL_7265455, EPI_ISL_7265456	Alpha Labs	National Microbiology Laboratory (NML)	Anna Majer; Anneliese Landgraff; CanCOGeN's metadata curation team; Darian Hole; Dynacare Brampton COVID-19 Diagnostic team; Elsie Grudeski; Gary Van Domselaar; Gordon Jolly; Grace Seo; Jennifer Tanner; Madison Chapel; Morag Graham; Natalie Knox; Nathalie Bastien; Philip Mabon; Public Health Agency of Canada CanCOGeN team; Rhiannon Huzarewich; Russell Mandes; Shari Tyson; Timothy Booth; Yan Li
EPI_ISL_6914011, EPI_ISL_6914012, EPI_ISL_6914013, EPI_ISL_6914014, EPI_ISL_6914015, EPI_ISL_6914016, EPI_ISL_6914017, EPI_ISL_6914018, EPI_ISL_6914019, EPI_ISL_6914020, EPI_ISL_6914021, EPI_ISL_6914022, EPI_ISL_6914023, EPI_ISL_6914024, EPI_ISL_6914025, EPI_ISL_6914026, EPI_ISL_6914027, EPI_ISL_6914028, EPI_ISL_6914029, EPI_ISL_6914030, EPI_ISL_6914031, EPI_ISL_6914032, EPI_ISL_6914033, EPI_ISL_6914034, EPI_ISL_6914035, EPI_ISL_6914036			
see above	Ampath Laboratories	National Institute for Communicable Diseases of the National Health Laboratory Service	Amoako DG; Bhiman JN; Everatt J; Ismail A; Mahlangu B; Mnguni A; Mohale T; Ntuli N; Scheepers C; Wolter N
EPI_ISL_7605587, EPI_ISL_7605639, EPI_ISL_7605640, EPI_ISL_7605743, EPI_ISL_7605745	Anglo American	National Institute for Communicable Diseases of the National Health Laboratory Service	Amoako DG; Bhiman JN; Everatt J; Ismail A; Mahlangu B; Mnguni A; Mohale T; Ntuli N; Scheepers C; Wolter N
EPI_ISL_7011321, EPI_ISL_7568602	Area of Virology, Serology and Virology Division (SAViD), New South Wales Health Pathology Randwick	Virology Research Laboratory; Area of Virology, Serology and Virology Division (SAViD), New South Wales Health Pathology Randwick	Au, J.; Bull, R.; Deveson, I.; Foster, C.; Rawlinson, W.; Ruiz Silva, M.; Van Hal, S.
EPI_ISL_7503376, EPI_ISL_7503377, EPI_ISL_7503378, EPI_ISL_7548950, EPI_ISL_7548951, EPI_ISL_7548952	Arizona State University	Arizona State University	Efrem S. Lim; Joshua LaBaer; LaRinda A. Holland; Matthew F. Smith; Nathaniel Johnson; Regan A. Sullins; Steven C. Holland; Vel Murugan
EPI_ISL_6963510	Auriga Research Pvt.Ltd / Strand Life Sciences	National Centre for Biological Sciences, TIFR - Rockefeller Foundation	Aarati Karaba; Anson Kunjumon George; Aparnaa Ramanathan; Apurva Sarin; Chandrasekhar Vadlamudi; Chitra Pattabiraman; Darshan Sreenivas; Dasaradhi Palakodeti; Dimple Notani; Divya Priya A; Madhusudhan J; Manisha Bharadwaj; Manoj Kumar Jha; Mudasir Nazaar; Pradeep B P; Priyanka Ananta Mulay; Ramesh Hariharan; Rohan Pais; Satyajit Mayor; Saumitra Mardikar; Srivathsan Adimoolam; Uma Ramakrishnan; Vamsi Veeramachaneni; Vasanthapuram Ravi; Vijay Chandru; Vishal G Rao; Yasodha Kannan
EPI_ISL_7621950, EPI_ISL_7621951, EPI_ISL_7621953, EPI_ISL_7621957	Australian Clinical Labs (formerly Healthscope Pathology)	NSW Health Pathology - Institute of Clinical Pathology and Medical Research; Westmead Hospital; University of Sydney	Arnott A.; Draper J.; Gall M.; Martinez E.; Rockett R.; Sintchenko V.; on behalf of ICPMR
EPI_ISL_7195246,	Austrian Agency for Health and Food	Bergthaler laboratory, CeMM Research	Andreas Bergthaler; Anna Schedl; Bekir Erguner; Benedikt Agerer; Christoph Bock; Fabian Amman; Jan Laine; Lukas Endler; Martin Senekowitsch; Matthew Thornton; Michael Schuster; Michelle Chan; Petr Triska; Thomas Penz

EPI_ISL_7195247, EPI_ISL_7195248	Safety (AGES)	Center for Molecular Medicine of the Austrian Academy of Sciences	
EPI_ISL_7502111, EPI_ISL_7616000	Ayass Bioscience LLC	Ayass Bioscience LLC	Kevin Zhu; Lina Abi Mosleh; Mohamad Ammar Ayass; Natalya Griko; Nazanin Taheri
EPI_ISL_7400565	Azienda Ospedaliera Pugliese Ciaccio di Catanzaro SOC Microbiologia e Virologia	Azienda Ospedaliera Pugliese Ciaccio di Catanzaro SOC Microbiologia e Virologia	Rossana Tallerico Cinzia Peronace Federica Pasceri Marco De Fazio Ilenia Talotta Giuseppina Panduri Pasquale Minchella
EPI_ISL_7338921	Azienda Sanitaria dell´Alto Adige - Laboratorio Aziendale di Microbiologia e Virologia	Azienda Sanitaria dell'Alto Adige	Irene Bianconi
EPI_ISL_7226262	BNH Hospital	National Institute of Health, Department of Medical Sciences, Ministry of Public Health, Thailand	Archawin Rojanawiwat; Ballang Uppapong; Beth Skaggs; Donlaya Maunplueng; Kazuhisa Okada; Nuttida Thongpramul; Pakorn Piromtong; Pilailuk Akkapaiboon Okada; Piroon Jenjaroenpun; Pongpun Sawatwong; Prapat Suriyaphol; Sirikanda Wimol; Siripaporn Phuygun; Sittiporn Parnmen; Supakit Sirilak; Suratchana Mitrat; Thanutsapa Thanadachakul; Thidathip Wongsurawat
EPI_ISL_7612892	BUMC Transplant Immunology Lab	BUMC Transplant Immunology Lab	Amanda Willis; Chiensao Wang; Jenifer Williams; Lynne Klingman; Medhat Askar; Pete Dysert
EPI_ISL_7565149, EPI_ISL_7565152	Bari	University of Bari Biomedical Sciences and Human Oncology	Maria Chironna
EPI_ISL_7149647, EPI_ISL_7197950, EPI_ISL_7346925, EPI_ISL_7347257, EPI_ISL_7347522, EPI_ISL_7355779, EPI_ISL_7391885, EPI_ISL_7391908, EPI_ISL_7391931, EPI_ISL_7391984, EPI_ISL_7392005, EPI_ISL_7392027, EPI_ISL_7392079, EPI_ISL_7392087, EPI_ISL_7392093, EPI_ISL_7392139, EPI_ISL_7485998, EPI_ISL_7486106, EPI_ISL_7486193, EPI_ISL_7486452, EPI_ISL_7486697, EPI_ISL_7486742, EPI_ISL_7486808, EPI_ISL_7486814, EPI_ISL_7486850, EPI_ISL_7486923, EPI_ISL_7487524, EPI_ISL_7487623, EPI_ISL_7487665, EPI_ISL_7487692, EPI_ISL_7512352, EPI_ISL_7512373, EPI_ISL_7512378, EPI_ISL_7512381, EPI_ISL_7512410, EPI_ISL_7512438, EPI_ISL_7512444, EPI_ISL_7512448, EPI_ISL_7520500, EPI_ISL_7538419, EPI_ISL_7538437, EPI_ISL_7538529, EPI_ISL_7538539, EPI_ISL_7538562, EPI_ISL_7538591, EPI_ISL_7538628, EPI_ISL_7538662, EPI_ISL_7538687, EPI_ISL_7538812, EPI_ISL_7538904, EPI_ISL_7538983, EPI_ISL_7538987, EPI_ISL_7539099, EPI_ISL_7539114, EPI_ISL_7539140, EPI_ISL_7539157, EPI_ISL_7539216, EPI_ISL_7539258, EPI_ISL_7539330, EPI_ISL_7539539, EPI_ISL_7539677, EPI_ISL_7539761			
see above	Berkshire and Surrey Pathology Services Lighthouse Laboratory	Wellcome Sanger Institute for the COVID-19 Genomics UK (COG-UK) Consortium	Berkshire and Surrey Pathology Services Lighthouse Laboratory and Alex Alderton; Cordelia Langford; David K. Jackson; Dominic Kwiatkowski; Ewan Harrison; Ian Johnston; Jeffrey Barrett; John Sillitoe on behalf of the Wellcome Sanger Institute COVID-19 Surveillance Team; Roberto Amato; Sonia Goncalves
EPI_ISL_7464464, EPI_ISL_7464465, EPI_ISL_7662094, EPI_ISL_7662095	Biolab Diagnostic Laboratories	Biolab Diagnostic Laboratories	Ahmad Tibi; Amid Abdelnour; Badia Saddedin; Eiad Atwa; Issa Abu-Dayyeh; Lama Hussein; Shaima Ali
EPI_ISL_7042354	Biolytix AG	Clinical Bacteriology, University Hospital Basel	Adrian Egli; Alfredo Mari; Fanny Wegner; Hans Hirsch; Helena MB Seth-Smith; Julia Bielicki; Karoline Leuzinger; Manuel Battegay; Tim Roloff
EPI_ISL_7417514	Bioscientia Labor Wermsdorf	Robert Koch Institute	
EPI_ISL_7544135, EPI_ISL_7544479, EPI_ISL_7544573	Bothasig CDC wc BLD	NHLS/UCT	Arash Iranzadeh; Bruna Galvao; Carolyn Williamson; Deelan Doolabh; Diana Hardie; Gert Marais; Innocent Mudau; Luicer Olubayo; Lynn Tyers; Marvin Hsiao; Nokuzola Mbhele; Rageema Joseph; Stephen Korsman
EPI_ISL_7548902, EPI_ISL_7548903, EPI_ISL_7548904, EPI_ISL_7548905, EPI_ISL_7548906, EPI_ISL_7548909, EPI_ISL_7548914, EPI_ISL_7548917, EPI_ISL_7548931, EPI_ISL_7552698, EPI_ISL_7552699, EPI_ISL_7566328, EPI_ISL_7566330, EPI_ISL_7566360			
see above	Botswana Harvard HIV Reference Laboratory	Botswana Harvard AIDS Institute Partnership, Plot 1836 North Ring Road, Princess Marina Hospital, Gaborone	Boitumelo Zuze; Botshelo Radibe; Dorcas Maruapula; Doreen Ditshwanelo; Joseph Makhema; Keoratile Ntshambiwa; Kgomotso Moruisi; Legodile Kooepile; Mosepele Mosepele; Mphaphi B. Mbulawa; Ontlametse T. Bareng; Pamela Smith-Lawrence; Roger Shapiro; Sefetogi Ramaologa; Shahin Lockman; Sikhulile Moyo; Simani Gaseitsiwe; Thongbotho Mphoyakgosi; Wonderful T. Choga
EPI_ISL_6640916, EPI_ISL_6640917, EPI_ISL_6640919, EPI_ISL_6670244, EPI_ISL_6752026, EPI_ISL_6752027, EPI_ISL_6774081, EPI_ISL_6774083, EPI_ISL_6774084, EPI_ISL_6774085, EPI_ISL_6774087, EPI_ISL_6774088, EPI_ISL_6774089, EPI_ISL_6774090, EPI_ISL_6774091, EPI_ISL_6774093, EPI_ISL_7121195, EPI_ISL_7380512, EPI_ISL_7380515, EPI_ISL_7380524			
see above	Botswana Harvard HIV Reference Laboratory	Botswana Harvard HIV Reference Laboratory	Boitumelo Zuze; Botshelo Radibe; Dorcas Maruapula; Joseph Makhema; Keoratile Ntshambiwa; Kgomotso Moruisi; Legodile Kooepile; Mosepele Mosepele; Mphaphi B. Mbulawa; Ontlametse T. Bareng; Pamela Smith-Lawrence; Roger Shapiro; Sefetogi Ramaologa; Shahin Lockman; Sikhulile Moyo; Simani Gaseitsiwe; Thongbotho Mphoyakgosi; Wonderful T. Choga
EPI_ISL_7132804, EPI_ISL_7370157, EPI_ISL_7370181, EPI_ISL_7370259, EPI_ISL_7370472	British Columbia Centre For Disease Control	BCCDC Public Health Laboratory	655 W 12th Avenue; Ana Pacagnella; BC Canada V5T 2N3; Corrinne Ng; Dan Fornika; John Tyson; Kim Macdonald; Kimia Kamelian; Linda Hoang; Loretta Janz; Mel Krajden; Prystajecky Natalie; Robert Azana; Shannon Russell; Vancouver
EPI_ISL_7390696, EPI_ISL_7391208, EPI_ISL_7453171, EPI_ISL_7453991, EPI_ISL_7454277, EPI_ISL_7542815, EPI_ISL_7543162, EPI_ISL_7543543, EPI_ISL_7543579, EPI_ISL_7544427, EPI_ISL_7544520, EPI_ISL_7547858, EPI_ISL_7548157, EPI_ISL_7550313, EPI_ISL_7550474, EPI_ISL_7550515, EPI_ISL_7550529, EPI_ISL_7550748, EPI_ISL_7658881, EPI_ISL_7658932			
see above	Broad Institute Clinical Research Sequencing Platform	Infectious Disease Program, Broad Institute of Harvard and MIT	Adams, G.; B.L.; B.W.; Bauer, M.; Birren; Blumenstiel, B.; Brown, C.; Carter, A.; Chaluvadi, S.; D.J.; DeFelice, M.; DeRuff, K.; Dodge, S.; Gabriel, S.; Gallagher, G.; Gladden-Young, A.; Granger, B.; J.E.; K.J.; Lagerborg, K.; Larkin, K.; Lee, M.; Lemieux; Lennon, N.; Loreth, C.; Madoff, L.; McGovern, S.; Meldrim, J.; Normandin, E.; P.C.; Park; Pearlman, L.; Reilly, S.; Rudy, M.; Sabeti; Siddle; Smole, S.; Tomkins-Tinch, C.; Vicente, G.; and MacInnis
EPI_ISL_7263830	CAP Roger de Flor	Banc de Sang i Teixits	Carlos Hobeich; Francisco Vidal; Irene Corrales; Lorena Ramírez; Maria Glòria Soria; Natália Comes; Nina Borràs; Noemí Gonzalez; Silvia Sauleda
EPI_ISL_7477252	CDPH VBL	California Department of Public Health	Emily Smith on behalf of CDPH-COVIDNet
EPI_ISL_7427856	CENTOGENE Frankfurt Laboratory: Niederlassung Industriepark Höchst	Robert Koch Institute	
EPI_ISL_7547731	CENTOLAB	National Reference Laboratory, Nigeria Centre for Disease Control	Catherine Okoi; Chimaobi Chukwu; Dr Ifedayo Adetifa; Dr Ndodo Nnaemeka; Dr Omoare Adesuyi; Nwando Mba; Olajumoke Babatunde; Olusola Anuoluwapo Akanbi; Oyeronke Ayansola
EPI_ISL_6862897	CERBALLIANCE	UMR PIMIT	David A Wilkinson; Patrick Mavingui
EPI_ISL_6962948, EPI_ISL_6967758	CERBALLIANCE PARIS ET IDF EST	CERBA HealthCare	Bénédicte Roquebert; Johanna Roux; Judith Zerah; Laura Verdurme; Sabine Trombert; Stéphanie Haim-Boukobza
EPI_ISL_7650614, EPI_ISL_7650626	CHU BORDEAUX	CHU BORDEAUX	Agnès George-Walryck; Laurent Busson; Marie-Edith Lafon; Pantxika Bellecave; Valentine Lesourd-Aubert
EPI_ISL_7226961	CHU de Bordeaux	CNR Virus des Infections Respiratoires - France SUD	Antonin Bal; Bruno Lina; Bruno Simon; Denis Malvy; Gregory Destras; Gwendolyne Burfin; Hadrien Regue; Laurence Josset; Marie-Edith Lafon; Martine Valette; Pantxika Bellecave; Quentin Semanas
EPI_ISL_7268670, EPI_ISL_7269933	CLINA-LANCET LABORATORIES	National Reference Laboratory, Nigeria Centre for Disease Control	Catherine Okoi; Chimaobi Chukwu; Dr Ifedayo Adetifa; Dr Ndodo Nnaemeka; Dr Omoare Adesuyi; Nwando Mba; Olajumoke Babatunde; Olusola Anuoluwapo Akanbi; Oyeronke Ayansola
EPI_ISL_7313633	CT Department of Public Health	CT Department of Public Health	Claire Pearson; Tu N. Nguyen
EPI_ISL_7137326, EPI_ISL_7137327, EPI_ISL_7137328, EPI_ISL_7137330	California Department of Public Health	California Department of Public Health	CDPH IDLB COVIDNet
EPI_ISL_7217437, EPI_ISL_7217563	Cantacuzino National Military-Medical Institute, Viral Respiratory Infections Laboratory	Cantacuzino Institute Virology	Luiza Ustea; Mihaela Lazar; Mihaela Oprea; Nicoleta Paraschiv; Sorin Dinu
EPI_ISL_7660838	Center of Medical Microbiology, Virology, and Hospital Hygiene, University of Duesseldorf	Center of Medical Microbiology, Virology, and Hospital Hygiene, University of Duesseldorf	Alexander Dilthey; Andreas Walker; Daniel Strelow; Jessica Nicolai; Jörg Timm; Klaus Pfeffer; Lisanna Hülse; Malte Kohns Vasconcelos; Maximilian Damagnez; Nadine Lübke; Tobias Wienemann; Torsten Houwaart
EPI_ISL_7571582	Centre Hospitalier Universitaire Clermont-Ferrand	CHU Clermont-Ferrand, service de virologie	Bisseux Maxime; Combes Patricia; Henquell Cecile; Mirand Audrey
EPI_ISL_6980876	Cerballiance, Reunion	UMR PIMIT	David A Wilkinson; Patrick Mavingui
EPI_ISL_7019047	Charité Universitätsmedizin Berlin, Institute of Virology	Charité Universitätsmedizin Berlin, Institute of Virology	Barbara Mühlemann; Christian Drosten; Julia Schneider; Julia Tesch; Jörn Beheim-Schwarzbach; Talitha Veith; Terry Jones; Tobias Bleicker; Victor M Corman
EPI_ISL_7337463, EPI_ISL_7337464, EPI_ISL_7337465, EPI_ISL_7337466, EPI_ISL_7337468, EPI_ISL_7337469, EPI_ISL_7337470, EPI_ISL_7337471, EPI_ISL_7337472, EPI_ISL_7337473, EPI_ISL_7337474, EPI_ISL_7337475, EPI_ISL_7337476, EPI_ISL_7337477, EPI_ISL_7337478, EPI_ISL_7337479, EPI_ISL_7337480, EPI_ISL_7337481, EPI_ISL_7337482, EPI_ISL_7337483, EPI_ISL_7337484, EPI_ISL_7337485, EPI_ISL_7337486, EPI_ISL_7337487, EPI_ISL_7337488, EPI_ISL_7337489, EPI_ISL_7337490, EPI_ISL_7337495, EPI_ISL_7337496, EPI_ISL_7337497, EPI_ISL_7337498, EPI_ISL_7337499, EPI_ISL_7337500, EPI_ISL_7337501, EPI_ISL_7337503, EPI_ISL_7337504, EPI_ISL_7337505, EPI_ISL_7337506, EPI_ISL_7337507, EPI_ISL_7337508, EPI_ISL_7337509, EPI_ISL_7337510, EPI_ISL_7337511			
see above	Charlotte Maxeke Johannesburg Academic Hospital	National Institute for Communicable Diseases of the National Health Laboratory Service	Amoako DG; Bhiman JN; Everatt J; Ismail A; Mahlangu B; Mnguni A; Mohale T; Ntuli N; Scheepers C
EPI_ISL_7337512, EPI_ISL_7337513, EPI_ISL_7337515, EPI_ISL_7337516, EPI_ISL_7337517, EPI_ISL_7337518, EPI_ISL_7337520, EPI_ISL_7337521, EPI_ISL_7337524, EPI_ISL_7337525, EPI_ISL_7337527, EPI_ISL_7337528, EPI_ISL_7337531, EPI_ISL_7337532, EPI_ISL_7337533, EPI_ISL_7337534, EPI_ISL_7337535, EPI_ISL_7337536, EPI_ISL_7337537, EPI_ISL_7337538, EPI_ISL_7337539, EPI_ISL_7337540, EPI_ISL_7337541, EPI_ISL_7337542, EPI_ISL_7337543, EPI_ISL_7337544, EPI_ISL_7337545, EPI_ISL_7337546, EPI_ISL_7337547, EPI_ISL_7337548, EPI_ISL_7337549, EPI_ISL_7337550, EPI_ISL_7337551, EPI_ISL_7337552, EPI_ISL_7337553, EPI_ISL_7337558, EPI_ISL_7337559, EPI_ISL_7337560, EPI_ISL_7337561, EPI_ISL_7337562, EPI_ISL_7337563, EPI_ISL_7337564			
see above	Chris Hani Baragwanath Laboratory	National Institute for Communicable Diseases of the National Health Laboratory Service	Amoako DG; Bhiman JN; Everatt J; Ismail A; Mahlangu B; Mnguni A; Mohale T; Ntuli N; Scheepers C
EPI_ISL_7154390	Clina-Lancet	National Reference Laboratory, Nigeria Centre for Disease Control	Catherine Okoi; Chimaobi Chukwu; Dr Ifedayo Adetifa; Dr Ndodo Nnaemeka; Dr Omoare Adesuyi; Nwando Mba; Olajumoke Babatunde; Olusola Anuoluwapo Akanbi; Oyeronke Ayansola
EPI_ISL_7265967, EPI_ISL_7265976, EPI_ISL_7265979, EPI_ISL_7266027, EPI_ISL_7266045, EPI_ISL_7266056, EPI_ISL_7266083, EPI_ISL_7552013, EPI_ISL_7552178, EPI_ISL_7552184, EPI_ISL_7552302, EPI_ISL_7552311, EPI_ISL_7552313, EPI_ISL_7552316, EPI_ISL_7552499, EPI_ISL_7552502, EPI_ISL_7552514, EPI_ISL_7552588, EPI_ISL_7552623, EPI_ISL_7552635, EPI_ISL_7552636			

see above	Clinical Microbiology Laboratory, Tel Aviv Sourasky Medical Center	Clinical Microbiology Laboratory, Tel Aviv Sourasky Medical Center	Alon Ziv; Amos Adler; Katya Levytskyi; Lior Handler; Ora Halutz
EPI_ISL_7042161, EPI_ISL_7042168, EPI_ISL_7462220, EPI_ISL_7462233, EPI_ISL_7462310, EPI_ISL_7462311, EPI_ISL_7462312			
see above	Clinical Virology	Clinical Bacteriology, University Hospital Basel	Adrian Egli; Alfredo Mari; Fanny Wegner; Hans Hirsch; Helena MB Seth-Smith; Julia Bielicki; Karoline Leuzinger; Manuel Battegay; Tim Roloff
EPI_ISL_7373598	Clinical Virology, Children's Hospital Los Angeles	Clinical Virology, Children's Hospital Los Angeles	Alexander Judkins; Cheryl Pool; Javier Mestas; Jennifer Dien Bard; John Fissel; Maurice O'Gorman
EPI_ISL_7462438	Cliniques universitaires Saint-Luc	UCLouvain/IREC/MBLG-CTMA	Benoit Kabamba Mukadi; Bertrand Bearzatto; Jean-Luc Gala; Nicolas Pinte; Paul Blanpain; Simon Ophélie; Valentin Coste
EPI_ISL_6951145	Color Genomics	Chiu Laboratory, University of California, San Francisco	Alicia Sotomayor-Gonzalez; Alicia Zhou; Amy Garlin; Charles Chiu; Darpun Sachdev; Katherine Hernandez; Scott Topper; Susan Philip; Venice Servellita; Yueyuan Zhang
EPI_ISL_7010485	Colorado Department of Public Health and Environment	Colorado Department of Public Health and Environment	Alexandria Rossheim; Diana Ir; Emily A. Travanty; Laura Bankers; Mandy Waters; Michael A. Martin; Molly C. Hetherington-Rauth; Sarah Elizabeth Totten; Shannon R. Matzinger
EPI_ISL_7613413	Community Labs, San Antonio	STRL UT Health San Antonio, Greehey Children's Cancer Research Institute	Bethany Landry; Dawn Garcia; Guillermo Nunez; Hongxin Fan; Josefina Stoever; Korri Weldon; Kumari Vadlamudi; Marjorie Parker David; San Antonio Metropolitan Health District; Texas Department of State Health Services; Weijing He; Yidong Chen; Zhao Lai; Zhenqing Ye
EPI_ISL_7451063, EPI_ISL_7451073, EPI_ISL_7451079, EPI_ISL_7451089	Cruz Vermelha Portuguesa	Instituto Nacional de Saude (INSA)	Borges et al
EPI_ISL_7609869	Curative Labs	Curative Labs	Elias L. Salfati; Eugenia Khorosheva; George Way; J.Cesar Ignacio-Espinoza; Janet Chen; Mikhail Hanewich-Hollatz; Nabjot Sandhu; Sophia Quasem; Vladimir Slepnev; Zhiyi Xie
EPI_ISL_7655648	DC Public Health Lab/ Dept. of Forensic Sciences	DC Public Health Lab/ Dept. of Forensic Sciences	Brittany Hamilton; Connie Maza; Elizabeth Zelaya; Eric Vaughn; Janis Doss; Jocelyn Hauser; Monica Mann; Nathan Bruns; Sarah Scott; Scott Nguyen; Wadih Bchara
EPI_ISL_7605689, EPI_ISL_7605739, EPI_ISL_7605775	DE AAR LABORATORY	National Institute for Communicable Diseases of the National Health Laboratory Service	Amoako DG; Bhiman JN; Everatt J; Ismail A; Mahlangu B; Mnguni A; Mohale T; Ntuli N; Scheepers C; Wolter N
EPI_ISL_7062087, EPI_ISL_7063588, EPI_ISL_7272249, EPI_ISL_7514034, EPI_ISL_7514991, EPI_ISL_7515043, EPI_ISL_7515374, EPI_ISL_7516043, EPI_ISL_7517471, EPI_ISL_7517646, EPI_ISL_7518103, EPI_ISL_7518795, EPI_ISL_7520214, EPI_ISL_7520543, EPI_ISL_7520819, EPI_ISL_7523657, EPI_ISL_7525859, EPI_ISL_7526218, EPI_ISL_7528707, EPI_ISL_7529040, EPI_ISL_7529817, EPI_ISL_7530609, EPI_ISL_7532336, EPI_ISL_7532391, EPI_ISL_7533005, EPI_ISL_7534407, EPI_ISL_7534541, EPI_ISL_7596661, EPI_ISL_7596668, EPI_ISL_7596669, EPI_ISL_7596670, EPI_ISL_7596683, EPI_ISL_7596684, EPI_ISL_7596696, EPI_ISL_7596697, EPI_ISL_7596702, EPI_ISL_7599427, EPI_ISL_7599604, EPI_ISL_7599994, EPI_ISL_7600175, EPI_ISL_7600348, EPI_ISL_7600349, EPI_ISL_7600356, EPI_ISL_7600365, EPI_ISL_7600506, EPI_ISL_7600699, EPI_ISL_7600710, EPI_ISL_7600940, EPI_ISL_7600946, EPI_ISL_7601285, EPI_ISL_7601634, EPI_ISL_7601635, EPI_ISL_7601645, EPI_ISL_7601647, EPI_ISL_7601691, EPI_ISL_7602008, EPI_ISL_7602017, EPI_ISL_7602018, EPI_ISL_7602026, EPI_ISL_7602027, EPI_ISL_7602028, EPI_ISL_7602031, EPI_ISL_7602182, EPI_ISL_7602236, EPI_ISL_7602366, EPI_ISL_7602373, EPI_ISL_7602581, EPI_ISL_7602582, EPI_ISL_7602583, EPI_ISL_7602584, EPI_ISL_7602585, EPI_ISL_7602591, EPI_ISL_7602592, EPI_ISL_7602593, EPI_ISL_7602594, EPI_ISL_7602601, EPI_ISL_7602607, EPI_ISL_7602608, EPI_ISL_7602609, EPI_ISL_7602610, EPI_ISL_7602617, EPI_ISL_7602618, EPI_ISL_7602619, EPI_ISL_7602620, EPI_ISL_7602625, EPI_ISL_7602626, EPI_ISL_7602627, EPI_ISL_7602628, EPI_ISL_7602636, EPI_ISL_7602637, EPI_ISL_7602638, EPI_ISL_7602644, EPI_ISL_7602645, EPI_ISL_7602646, EPI_ISL_7602647, EPI_ISL_7602655, EPI_ISL_7602656, EPI_ISL_7602657, EPI_ISL_7602658, EPI_ISL_7602664, EPI_ISL_7602665, EPI_ISL_7602666, EPI_ISL_7602667, EPI_ISL_7602673, EPI_ISL_7602674, EPI_ISL_7602675, EPI_ISL_7602676, EPI_ISL_7602685, EPI_ISL_7602686, EPI_ISL_7638530, EPI_ISL_7638596, EPI_ISL_7638689, EPI_ISL_7638718, EPI_ISL_7639039, EPI_ISL_7639117, EPI_ISL_7639395, EPI_ISL_7639484, EPI_ISL_7640293, EPI_ISL_7640569, EPI_ISL_7640706, EPI_ISL_7640724, EPI_ISL_7640969, EPI_ISL_7641033, EPI_ISL_7642298, EPI_ISL_7642471, EPI_ISL_7642555, EPI_ISL_7642579, EPI_ISL_7642615, EPI_ISL_7642958, EPI_ISL_7642986, EPI_ISL_7643084, EPI_ISL_7643799, EPI_ISL_7644130, EPI_ISL_7644179, EPI_ISL_7644186, EPI_ISL_7644799, EPI_ISL_7644806, EPI_ISL_7644811, EPI_ISL_7644892, EPI_ISL_7645092, EPI_ISL_7645159, EPI_ISL_7645256, EPI_ISL_7645265, EPI_ISL_7645273, EPI_ISL_7648027, EPI_ISL_7648227, EPI_ISL_7648272, EPI_ISL_7648309, EPI_ISL_7648310, EPI_ISL_7648328, EPI_ISL_7648332, EPI_ISL_7648333, EPI_ISL_7648343, EPI_ISL_7648344, EPI_ISL_7648345, EPI_ISL_7648346, EPI_ISL_7648353, EPI_ISL_7648354, EPI_ISL_7648355, EPI_ISL_7648356, EPI_ISL_7648357, EPI_ISL_7648358, EPI_ISL_7648359, EPI_ISL_7648360, EPI_ISL_7648361, EPI_ISL_7648362, EPI_ISL_7648363, EPI_ISL_7648364, EPI_ISL_7648365, EPI_ISL_7648366, EPI_ISL_7648367, EPI_ISL_7648368, EPI_ISL_7648457			
see above	Department of Bacteria, Parasites and Fungi, Statens Serum Institut, Copenhagen, Denmark	Statens Serum Institut Bioinformatics and Microbial Genomics	Danish Covid-19 Genome Consortium
EPI_ISL_7042669	Department of Clinical Microbiology	GIGA Medical Genomics	Bouchra Boujemla; Claire Gourzonès; Cécile Meex; Keith Durkin; Laurent Gillet; Maria Artesi; Marie-Pierre Hayette; Nadine Cambisano; Nathalie Renotte; Olivier Ek; Sébastien Bontems; Vincent Bours
EPI_ISL_7512899, EPI_ISL_7520649, EPI_ISL_7531709, EPI_ISL_7533537, EPI_ISL_7533749	Department of Clinical Microbiology, Odense University Hospital, Odense, Denmark	Statens Serum Institut Bioinformatics and Microbial Genomics	Danish Covid-19 Genome Consortium
EPI_ISL_7192723, EPI_ISL_7192733, EPI_ISL_7192734, EPI_ISL_7485700	Department of Health Technology and Informatics, The Hong Kong Polytechnic University	Department of Health Technology and Informatics, The Hong Kong Polytechnic University	Alan Ka-Lun Wu; Alex Yat-Man Ho; Barry Kin-Chung Wong; Chloe Toi-Mei Chan; David Ho-Keung Shum; Denise Sze-Hang Wong; Gilman Kit-Hang Siu; Hiu-Yin Lao; Hoi-Ching Jim; Ivan Tak-Fai Wong; Jake Siu-Lun Leung; Kam-Tong Yip; Kenneth Siu-Sing Leung; Kingsley King-Gee Tam; Kitty Sau-Chun Fung; Kristine Luk; Lam-Kwong Lee; Miranda Chong-Yee Yau; Sandy Ka-Yee Chau; Shea Ping Yip; Tak-Lun Que; Timothy Ting-Leung Ng; Wing Cheong Yam; Wing-Hei Lo; Wing-Kin To; Yvette Wai-Man Lai
EPI_ISL_6841980, EPI_ISL_6841981, EPI_ISL_7138045, EPI_ISL_7357684, EPI_ISL_7385702	Department of Microbiology, The University of Hong Kong	Department of Microbiology, The University of Hong Kong	Kelvin K.W. To; Kwok-Yung Yuen
EPI_ISL_7571413	Department of Pathology and Laboratory Medicine, AKUH Laboratories, Karachi, Pakistan	Department of Virology	A. Kanji; A. Nasir; A. Samreen; A.R. Bukhari; Aamer Ikram; Abdul Ahad; J. Ashraf; Massab Umair; Muhammad Ammar; Muhammad Salman; Nazish Badar; Qasim Ali; R. Hasan; Syed Adnan Haider; U.B. Aamir; Z. Hasan; Zaira Rehman
EPI_ISL_7201444, EPI_ISL_7624156, EPI_ISL_7624213	Department of Virology and Immunology, University of Helsinki and Helsinki University Hospital, Huslab Finland	Department of Virology, Faculty of Medicine, University of Helsinki, Helsinki, Finland	Hanna Jarva; Hanna Liimatainen; Hanna Vauhkonen; Hussein Alburkat; Maija Lappalainen; Mert Erdin; Olli Vapalahti; Phuoc Truong; Ravi Kant; Sari Hannula; Satu Kurkela; Teemu Smura
EPI_ISL_6972689	Dept. of Laboratory Medicine	Dept. of Laboratory Medicine	Claudia Weber; Fabian Konig; Harald Esterbauer; Oswald Wagner; Robert Strassl; Sabina Plumer; Victoria Six
EPI_ISL_7464539, EPI_ISL_7464543	Dept. of Microbiology and Infection Control, Akershus University Hospital HF	Dept. of Microbiology and Infection Control, Akershus University Hospital HF	Alexander Hesselberg Løvestad; Hege Vangstein Aamot
EPI_ISL_6959926, EPI_ISL_6959935, EPI_ISL_6959993, EPI_ISL_7406117, EPI_ISL_7406118, EPI_ISL_7406119, EPI_ISL_7406124, EPI_ISL_7406125, EPI_ISL_7406126			
see above	Division of Emerging Infectious Diseases, Bureau of Infectious Diseases Diagnosis Control, Korea Disease Control and Prevention Agency	Division of Emerging Infectious Diseases, Bureau of Infectious Diseases Diagnosis Control, Korea Disease Control and Prevention Agency	Ae Kyung Park; Chae Young Lee; Eun-Jin Kim; Heui Man Kim; Hyuck Jin Lee; Il-Hwan Kim; Jeong-Ah Kim; Jeong-Min Kim
EPI_ISL_6842156, EPI_ISL_6842159, EPI_ISL_6842162, EPI_ISL_6842163, EPI_ISL_6842165, EPI_ISL_7452732, EPI_ISL_7452733, EPI_ISL_7452734, EPI_ISL_7452735, EPI_ISL_7452736, EPI_ISL_7452737, EPI_ISL_7452738, EPI_ISL_7452741, EPI_ISL_7452742, EPI_ISL_7452744, EPI_ISL_7452745, EPI_ISL_7452746, EPI_ISL_7452749, EPI_ISL_7452750, EPI_ISL_7452751, EPI_ISL_7452758, EPI_ISL_7452761, EPI_ISL_7452762, EPI_ISL_7452763, EPI_ISL_7452764, EPI_ISL_7452765, EPI_ISL_7452771, EPI_ISL_7452780, EPI_ISL_7452781, EPI_ISL_7452782, EPI_ISL_7452783, EPI_ISL_7452785, EPI_ISL_7452792, EPI_ISL_7452793, EPI_ISL_7452794, EPI_ISL_7452795, EPI_ISL_7452796, EPI_ISL_7452797, EPI_ISL_7452798, EPI_ISL_7452799, EPI_ISL_7452800, EPI_ISL_7456525, EPI_ISL_7456526, EPI_ISL_7544929			
see above	Division of Medical Virology, National Health Laboratory Service (NHLS), Tygerberg Hospital / Stellenbosch University	Division of Medical Virology, National Health Laboratory Service (NHLS), Tygerberg Hospital / Stellenbosch University	Gert van Zyl; Kamela Mahlakwane; Shannon Wilson; Susan Engelbrecht; Tania Stander; Tongai Maponga; Wolfgang Preiser
EPI_ISL_7062590, EPI_ISL_7506397, EPI_ISL_7506422, EPI_ISL_7506490	Dr. Risch Ostschweiz AG	Dr Risch Laboratory	Dominique Fabien Hilti; Faina Wehrli; Lorenz Risch; Martin Risch; Nadia Wohlwend; Sinem Kas; Thomas Bodmer
EPI_ISL_7651134	Dutch COVID-19 response team	Erasmus Medical Center	Anne van der Linden; Annemiek van der Eijk; Bas Oude Munnink; Corine GeurtsvanKessel; David Nieuwenhuijse; Emmanuelle Munger; Irina Chestakova; Marion Koopmans; Marjan Boter; Reina Sikkema; Richard Molenkamp; on behalf of the Dutch national COVID-19 response team.
EPI_ISL_7469070	Dutch COVID-19 response team	Medical Microbiology, Maastricht University Medical Centre	Brian van der Veer*; Carmen Reumkens; Christian Hoebe; Erik Beuken; Jozef Dingemans*; Lieke van Alphen; Paul Savelkoul
EPI_ISL_6841607, EPI_ISL_6841608, EPI_ISL_6841609, EPI_ISL_6841610, EPI_ISL_6841611, EPI_ISL_6841612, EPI_ISL_6841613, EPI_ISL_6841614, EPI_ISL_6841615, EPI_ISL_6841616, EPI_ISL_6841617, EPI_ISL_6841618, EPI_ISL_6841619, EPI_ISL_7470657, EPI_ISL_7471412, EPI_ISL_7471413, EPI_ISL_7471451, EPI_ISL_7471520, EPI_ISL_7471548, EPI_ISL_7471549, EPI_ISL_7471975			
see above	Dutch COVID-19 response team	National Institute for Public Health and the Environment (RIVM)	Adam Meijer; AnneMarie van den Brandt; Annelies Kroneman; Bas van der Veer; Chantal Reusken; Dennis Schmitz; Dirk Eggink; Florian Zwagemaker; Harry Vennema; Ivo van Walle; Jeroen Cremer; Jil Kocken; Karim Hajji; Kim Freriks; Linda van Someren; Lisa Wijsman; Lynn Aarts; Ryanne Jaarsma; Sanne Bos; Sharon van den Brink; Stijn van Rossum; on behalf of the national COVID-19 response team
EPI_ISL_6826713, EPI_ISL_6826714, EPI_ISL_6989667, EPI_ISL_7015154, EPI_ISL_7160041, EPI_ISL_7160042	Dynacare	National Microbiology Laboratory (NML)	Anna Majer; Anneliese Landgraff; CanCOGeN's metadata curation team; Darian Hole; Dynacare Brampton COVID-19 Diagnostic team; Elsie Grudeski; Gary Van Domselaar; Gordon Jolly; Grace Seo; Jennifer Tanner; Madison Chapel; Morag Graham; Natalie Knox; Nathalie Bastien; Philip Mabon; Public Health Agency of Canada CanCOGeN team; Rhiannon Huzarewich; Russell Mandes; Shari Tyson; Timothy Booth; Yan Li
EPI_ISL_7657584, EPI_ISL_7657586, EPI_ISL_7657588	EXCITE lab	Andersen lab at Scripps Research	Abigail Schnapper; Angela Scioscia; Cheryl Anderson; Chip Schooley; Greg Humphrey; Helena Tubb; Natasha Martin; Sawyer Farmer; Smruthi Karthikeyan; Tommy Valles + SEARCH
EPI_ISL_6949582, EPI_ISL_7123666, EPI_ISL_7123680, EPI_ISL_7123692, EPI_ISL_7330020, EPI_ISL_7330021, EPI_ISL_7330161, EPI_ISL_7464335, EPI_ISL_7464340, EPI_ISL_7464346, EPI_ISL_7660998, EPI_ISL_7660999			
see above	Edmonton Provincial Lab	Alberta Precision Labs (APL)	Buss; Croxen M; Deo A; Dieu P; E; Ferrato C; Gill K; Khan F; Koleva P; Li V; Lloyd C; Lynch T; Ma R; Murphy S; Pabbaraju K; Shokoples S; Thayer J; Tipples G; Whitehouse M; Wong A; Yu C; Zelyas N
EPI_ISL_7547732,	Everight diagnostics (Abuja)	National Reference Laboratory, Nigeria	Catherine Okoi; Chimaobi Chukwu; Dr Ifedayo Adetifa; Dr Ndodo Nnaemeka; Dr Omoare Adesuyi; Nwando Mba; Olajumoke Babatunde; Olusola Anuoluwapo Akanbi; Oyeronke Ayansola

EPI_ISL_7547733		Centre for Disease Control	
EPI_ISL_7313687	Florida Bureau of Public Health Laboratories	Florida Bureau of Public Health Laboratories	Jason Blanton; Namratha Tarigopula; Sarah Schmedes; Tiffany Splatt
EPI_ISL_7154399, EPI_ISL_7497709, EPI_ISL_7497711, EPI_ISL_7497739, EPI_ISL_7497740, EPI_ISL_7497741, EPI_ISL_7497742, EPI_ISL_7497767			
see above	Fulgent Genetics	Centers for Disease Control and Prevention Division of Viral Diseases, Pathogen Discovery	Becky Tsai; Benafsh Sapra; Benjamin Rambo-Martin; Christopher Gulvick; Clinton Paden; Dakota Howard; Dhwani Batra; Doreen Ng; Duncan MacCannell; Erisa Sula; Harry Gao; James Xie; Jason Caravas; John Gao; Joseph Fierro; Kristine Lacek; Matthew Schmerer; Mickey Li; Peter Cook; Scott Sammons; Shatavia Morrison; Tymeckia Kendall; Victoria Caban Figueroa; Yan Meng; Yvette Unoarumhi
EPI_ISL_6971572, EPI_ISL_6989155, EPI_ISL_7248772, EPI_ISL_7248778, EPI_ISL_7248797, EPI_ISL_7248805, EPI_ISL_7248814, EPI_ISL_7248821, EPI_ISL_7470261			
see above	Furst Medical Laboratory	Norwegian Institute of Public Health, Department of Virology	Atiya R Ali; Debech Nadia; Engebretsen Serina Beate; Garcia Llorente Ignacio; Hilde Elshaug; Hilde Vollan; Jon Bråte; Kamilla Heddeland Instefjord; Karoline Bragstad; Kathrine Stene-Johansen; Line Victoria Moen; Marie Paulsen Madsen; Olav Hungnes; Pedersen Benedikte Nevjen; Rasmus Riis Kopperud
EPI_ISL_7473158	GA Department of Public Health	GA Department of Public Health	Aliyah Fields; Jonathan Edwards; Sharmila Talekar; Stacy Reeves; Taylor Smith; Tonia Parrott
EPI_ISL_7173962	GENEPATH	NCL, Pune	Ajinkya Khilari; Anu Raghunathan; Bhagyashree Litikar; Dhanasekaran Shanmugam; Divya Niveditha; Jugal Kanekar; Shikha Takur
EPI_ISL_7313494, EPI_ISL_7478195, EPI_ISL_7478220, EPI_ISL_7478233, EPI_ISL_7657471, EPI_ISL_7657493	GH A.CHENEVIER-H.MONDOR	Department of Virology, Henri Mondor University Hospital, Assistance Publique Hôpitaux de Paris, Université Paris-Est Créteil, INSERM U955	Alexandre Soulier; Christophe Rodriguez; Elisabeth Trawinski; Guillaume Gricourt; Jean-Michel Pawlotsky; Melissa N'Debi; Slim Fourati; Vanessa Demontant
EPI_ISL_6892613, EPI_ISL_6892620, EPI_ISL_6892631, EPI_ISL_6892639, EPI_ISL_6892644, EPI_ISL_6892650, EPI_ISL_6892653, EPI_ISL_6892659, EPI_ISL_6892661, EPI_ISL_6892665, EPI_ISL_6892674, EPI_ISL_6892683, EPI_ISL_6892693			
see above	Germano de Sousa	Instituto Nacional de Saude (INSA)	Borges et al
EPI_ISL_7545349, EPI_ISL_7545360, EPI_ISL_7545585, EPI_ISL_7596392, EPI_ISL_7596401, EPI_ISL_7596417, EPI_ISL_7649865, EPI_ISL_7649923, EPI_ISL_7660709, EPI_ISL_7660731, EPI_ISL_7660740			
see above	Gibraltar Health Authority Lab	Gibraltar Health Authority Covid-19 Laboratory	Bruna Martins; Dr Daniel Cassaglia; Dr Martyn Bell; Dr Nicholas Cortes; Dr Zoe Vincent; Sofia Lavelle
EPI_ISL_7549110, EPI_ISL_7549111	Gravity Diagnostics, LLC	Gravity Diagnostics, LLC	Gravity Diagnostics
EPI_ISL_7544692	Great Brak River Clinic wc GBC	NHLS/UCT	Arash Iranzadeh; Bruna Galvao; Carolyn Williamson; Deelan Doolabh; Diana Hardie; Gert Marais; Innocent Mudau; Luicer Olubayo; Lynn Tyers; Marvin Hsiao; Nokuzola Mbhele; Rageema Joseph; Stephen Korsman
EPI_ISL_7543874, EPI_ISL_7544059, EPI_ISL_7544158, EPI_ISL_7544262, EPI_ISL_7544652	Groote Schuur Hospital wc GSH	NHLS/UCT	Arash Iranzadeh; Bruna Galvao; Carolyn Williamson; Deelan Doolabh; Diana Hardie; Gert Marais; Innocent Mudau; Luicer Olubayo; Lynn Tyers; Marvin Hsiao; Nokuzola Mbhele; Rageema Joseph; Stephen Korsman
EPI_ISL_6913917	Grupo CR Diagnosticos	Instituto Adolfo Lutz Strategic Laboratory	Claudio Tavares Sacchi; Karoline Rodrigues Campos
EPI_ISL_6900139, EPI_ISL_6900141, EPI_ISL_6900142, EPI_ISL_6900143	HELEN JOSEPH LABORATORY	National Institute for Communicable Diseases of the National Health Laboratory Service	Amoako DG; Bhiman JN; Everatt J; Ismail A; Mahlangu B; Mnguni A; Mohale T; Ntuli N; Scheepers C
EPI_ISL_6698790	HOME QUARANTINE TASKFORCE	Hong Kong Department of Health	Alan K.L. Tsang; Edman T.K. Lam; Ken H.L. Ng; Peter C.W. Yip; Rickjason C.W. Chan
EPI_ISL_7610402	HOPITAL SAINT ANDRE	CNR Virus des Infections Respiratoires - France SUD	Antonin Bal; Bruno Lina; Bruno Simon; Gregory Destras; Gwendolyne Burfin; Hadrien Regue; Laurence Josset; Martine Valette; Quentin Semanas
EPI_ISL_7649952	HOSPITAL MUNICIPAL DR JOSE DE CARVALHO FLORENCE	Instituto Butantan	Antonio Jorge Martins; Claudia Renata dos Santos Barros; David Schlesinger; Debora Botequio Moretti; Dimas Tadeu Covas; Elaine Cristina Marqueze; Elaine Vieira Santos; Evandra Strazza Rodrigues; Heidge Fukumasu; Jayme Augusto de Souza-Neto; Luiz Alcantara; Luiz Lehmann Coutinho; Maria Carolina Elias; Maurício Lacerda Nogueira; Rafael dos Santos Bezerra; Raul Machado Neto; Rejane Maria Tommasini Grotto; Ricardo Haddad; Sandra Coccuzzo Sampaio Vessoni; Simone Kashima; Svetoslav Nanev Slavov; Vincent Louis Viala
EPI_ISL_7045214, EPI_ISL_7571637, EPI_ISL_7571638	HOSPITAL UNIVERSITARI DE BELLVITGE	Microbiology Department	Aida Gonzalez-Diaz; Anna Carrera-Salinas Yolanda Hernandez; Carmen Ardanuy; Daniel Rodirguez; Jordi Camara; Jordi Niubó; Laura Calatayud; M Angeles Domínguez; Sara Marti; Veronica Saez; Yolanda Hernandez
EPI_ISL_7624161, EPI_ISL_7624188, EPI_ISL_7624204, EPI_ISL_7624230	HOSPITAL UNIVERSITARIO SON ESPASES	HOSPITAL UNIVERSITARIO SON ESPASES	Dr. Antonio Oliver; Dr. Carla López-Causapé; Dr. Gabriel Cabot; Hospital Universitario Son Espases; on behalf of Servicio de Microbiología
EPI_ISL_7565186, EPI_ISL_7565187	Harvard University	Infectious Disease Program, Broad Institute of Harvard and MIT	B.L.; D.J.; Dobbins, S.; K.J.; MacInnis; P.C.; Park; Petros, B.; Sabeti; Siddle; Slack, S.; Springer, M.; Welch, N.
EPI_ISL_7470201	Haukeland University Hospital, Dept. of Microbiology	Norwegian Institute of Public Health, Department of Virology	Atiya R Ali; Debech Nadia; Engebretsen Serina Beate; Garcia Llorente Ignacio; Hilde Elshaug; Hilde Vollan; Jon Bråte; Kamilla Heddeland Instefjord; Karoline Bragstad; Kathrine Stene-Johansen; Line Victoria Moen; Marie Paulsen Madsen; Olav Hungnes; Pedersen Benedikte Nevjen; Rasmus Riis Kopperud
EPI_ISL_7142185, EPI_ISL_7142260, EPI_ISL_7184579	Health Services Laboratories	Wellcome Sanger Institute for the COVID-19 Genomics UK (COG-UK) Consortium	Cordelia Langford; David K. Jackson; Dominic Kwiatkowski; Ewan Harrison; Health Services Laboratories and Alex Alderton; Ian Johnston; Jeffrey Barrett; John Sillitoe on behalf of the Wellcome Sanger Institute COVID-19 Surveillance Team; Roberto Amato; Sonia Goncalves
EPI_ISL_7544466	Helderberg Hospital wc HHH	NHLS/UCT	Arash Iranzadeh; Bruna Galvao; Carolyn Williamson; Deelan Doolabh; Diana Hardie; Gert Marais; Innocent Mudau; Luicer Olubayo; Lynn Tyers; Marvin Hsiao; Nokuzola Mbhele; Rageema Joseph; Stephen Korsman
EPI_ISL_7337440, EPI_ISL_7337441, EPI_ISL_7337442, EPI_ISL_7337443, EPI_ISL_7337444, EPI_ISL_7337445, EPI_ISL_7337446, EPI_ISL_7337447, EPI_ISL_7337448, EPI_ISL_7337449, EPI_ISL_7337450, EPI_ISL_7337451, EPI_ISL_7337452, EPI_ISL_7337453, EPI_ISL_7337454, EPI_ISL_7337455, EPI_ISL_7337458, EPI_ISL_7337460, EPI_ISL_7337461, EPI_ISL_7337462			
see above	Helen Joseph Laboratory	National Institute for Communicable Diseases of the National Health Laboratory Service	Amoako DG; Bhiman JN; Everatt J; Ismail A; Mahlangu B; Mnguni A; Mohale T; Ntuli N; Scheepers C
EPI_ISL_7497723	Helix	Centers for Disease Control and Prevention Division of Viral Diseases, Pathogen Discovery	Benjamin Rambo-Martin; Christopher Gulvick; Clinton Paden; Dakota Howard; Dhwani Batra; Duncan MacCannell; Erisa Sula; Helix CA; Jason Caravas; Kristine Lacek; Matthew Schmerer; Peter Cook; Scott Sammons; Shatavia Morrison; Tymeckia Kendall; Victoria Caban Figueroa; Yvette Unoarumhi
EPI_ISL_7265236, EPI_ISL_7265237, EPI_ISL_7462215, EPI_ISL_7620968, EPI_ISL_7620969, EPI_ISL_7620970, EPI_ISL_7620971, EPI_ISL_7620972, EPI_ISL_7620973, EPI_ISL_7620977, EPI_ISL_7621010, EPI_ISL_7621011, EPI_ISL_7621210, EPI_ISL_7621349, EPI_ISL_7621350, EPI_ISL_7621351, EPI_ISL_7621352, EPI_ISL_7621353, EPI_ISL_7621907, EPI_ISL_7621908, EPI_ISL_7621909, EPI_ISL_7621910, EPI_ISL_7621914, EPI_ISL_7621915, EPI_ISL_7621916, EPI_ISL_7621917			
see above	Histopath	NSW Health Pathology - Institute of Clinical Pathology and Medical Research; Westmead Hospital; University of Sydney	Arnott A.; Draper J.; Gall M.; Martinez E.; Rockett R.; Sintchenko V.; on behalf of ICPMR
EPI_ISL_6590782, EPI_ISL_6832108	Home Quarantine Taskforce	Hong Kong Department of Health	Alan K.L. Tsang; Edman T.K. Lam; Ken H.L. Ng; Peter C.W. Yip; Rickjason C.W. Chan
EPI_ISL_6716890, EPI_ISL_6716902	Hong Kong Department of Health	School of Public Health, The University of Hong Kong	Dominic N.C. Tsang; Haogao Gu; Leo L.M. Poon; Malik Peiris
EPI_ISL_7373061, EPI_ISL_7373165, EPI_ISL_7373230	Hospital General Universitario Albacete	Hospital General Universitario Albacete	Caridad Sainz de Baranda Camino; Lorena Robles-Fonseca
EPI_ISL_6851526, EPI_ISL_6902675, EPI_ISL_6971860, EPI_ISL_7042252, EPI_ISL_7604642, EPI_ISL_7604643, EPI_ISL_7604644, EPI_ISL_7604645, EPI_ISL_7604646, EPI_ISL_7604647, EPI_ISL_7604648, EPI_ISL_7604649, EPI_ISL_7604652, EPI_ISL_7604653, EPI_ISL_7604654, EPI_ISL_7604659, EPI_ISL_7604693, EPI_ISL_7604694, EPI_ISL_7604695, EPI_ISL_7604696, EPI_ISL_7604715			
see above	Hospital General Universitario Gregorio Marañón	Hospital General Universitario Gregorio Marañón	Cristina Rodriguez-Grande; Darío García de Viedma; Jorge Rodríguez-Grande; Julia Suárez; Laura Pérez-Lago; Marta Herranz Martin; Patricia Muñoz; Pedro Sola Campoy; Pilar Catalán; Sergio Buenestado Serrano; Victor Manuel de la Cueva
EPI_ISL_7571377, EPI_ISL_7571378, EPI_ISL_7571388, EPI_ISL_7571389	Hospital General Universitario de Ciudad Real	Hospital General Universitario de Ciudad Real	Cristina Colmenarejo; José Martínez-Alarcón; Lidia García-Agudo; Marta Torres-Narbona; Soledad Illescas Fernández-Bermejo
EPI_ISL_7204336	Hospital Universitari Dr. Josep Trueta	Institut d'Investigació Biomèdica de Girona | Hospital Universitari Dr. Josep Trueta	Bernat del Olmo; Mel·lina Pinsach; Meritxell Deulofeu; Nuria Esther Neto; Paula Costa
EPI_ISL_7598538	Hospital Universitari Joan XXIII de Tarragona	Hospital Universitari Vall d'Hebron - Vall d'Hebron Institut de Recerca	Alejandra González-Sánchez; Andrés Antón; Ariadna Rando; Carla Castillo; Cristina Andrés; Damir Garcia-Cehic; Josep Quer; Juliana Esperalba; Karen García; Maria Carmen Martin; Maria Gema Codina; Maria Piñana; Rodrigo Vásquez; Tomàs Pumarola
EPI_ISL_7050911, EPI_ISL_7050918, EPI_ISL_7406515, EPI_ISL_7598503,	Hospital Universitari Vall d'Hebron - Vall d'Hebron Institut de Recerca	Hospital Universitari Vall d'Hebron - Vall d'Hebron Institut de Recerca	Alejandra González-Sánchez; Andrés Antón; Ariadna Rando; Carla Castillo; Cristina Andrés; Damir Garcia-Cehic; Josep Quer; Juliana Esperalba; Karen García; Maria Carmen Martin; Maria Gema Codina; Maria Piñana; Rodrigo Vásquez; Tomàs Pumarola

EPI_ISL_7598554, EPI_ISL_7598569			
EPI_ISL_7277268	Hospital Universitario Son Espases	Hospital Universitario Son Espases	Dr. Antonio Oliver; Dr. Carla López-Causapé; Dr. Gabriel Cabot; Hospital Universitario Son Espases; on behalf of Servicio de Microbiología
EPI_ISL_7329633	Hospital Universitario de Guadalajara	Hospital General Universitario de Ciudad Real	Cristina Colmenarejo; José Martínez-Alarcón; Lidia García-Agudo; Marta Torres-Narbona; Soledad Illescas Fernández-Bermejo
EPI_ISL_7156454	Hospital Ángeles Lomas	Instituto de diagnóstico y Referencia Epidemiologicos (INDRE)	Abril Rodriguez-Maldonado; Ariadna Medina-Benitez; Armando Rojo; Claudia Wong-Arambula; Ernesto Ramirez-Gonzalez; Fernando Gonzalez-Dominguez; Gisela Barrera-Badillo; Irma Lopez-Martinez; Joaquin Quiroz-Mercado; Leonardo Medina Arias; Lucia Hernandez-Rivas; Maribel Gonzalez-Villa; Natividad Cruz-Ortiz; Pilar Escamilla Llano; Raymundo Rodríguez Sandoval; Tatiana Nunez-Garcia; Vanessa Rivero-Arredondo
EPI_ISL_7599319	Houston Health Dept.	Houston Health Dept.	Adolfo Lara; Pamela Brown; Ryker Penn; Yanlai Lai
EPI_ISL_7415721, EPI_ISL_7415723, EPI_ISL_7415731, EPI_ISL_7415752, EPI_ISL_7415767, EPI_ISL_7415770, EPI_ISL_7415774, EPI_ISL_7415830, EPI_ISL_7415887, EPI_ISL_7602881, EPI_ISL_7602890, EPI_ISL_7602896, EPI_ISL_7603271, EPI_ISL_7603282, EPI_ISL_7603681, EPI_ISL_7603695, EPI_ISL_7603810, EPI_ISL_7603844, EPI_ISL_7603977, EPI_ISL_7604046, EPI_ISL_7604065, EPI_ISL_7604211, EPI_ISL_7604216, EPI_ISL_7604217, EPI_ISL_7604227, EPI_ISL_7604250, EPI_ISL_7604285, EPI_ISL_7604367, EPI_ISL_7604480, EPI_ISL_7604505, EPI_ISL_7612854			
see above	Houston Methodist Hospital	Houston Methodist Hospital	Ilya J. Finkelstein; James J. Davis; Jessica Cambric; Jimmy Gollihar; Kristina Reppond; Layne Pruitt; Madison N. Shyer; Marcus Nguyen; Matthew Ojeda Saavedra; Paul A. Christensen; Prasanti Yerramilli; Randall J. Olsen; Robert Olson; Ryan Gadd; S. Wesley Long; Sishir Subedi; and James M. Musser
EPI_ISL_7210427, EPI_ISL_7635857	Hrvatski zavod za javno zdravstvo	Hrvatski zavod za javno zdravstvo	Anita Jurić; Dragan Jurić; Irena Tabain; Ivana Ferenčak; Josipa Kuzle; Ljiljana Žmak; Mihaela Obrovac
EPI_ISL_7154340, EPI_ISL_7156753, EPI_ISL_7308635, EPI_ISL_7308771, EPI_ISL_7308875, EPI_ISL_7381064, EPI_ISL_7552474, EPI_ISL_7552479, EPI_ISL_7552666, EPI_ISL_7552686, EPI_ISL_7552693, EPI_ISL_7602123, EPI_ISL_7602445, EPI_ISL_7602500, EPI_ISL_7602605, EPI_ISL_7602741, EPI_ISL_7602776, EPI_ISL_7602813			
see above	IHU Mediterranee Infection	IHU Mediterranee Infection	Philippe Colson et al.
EPI_ISL_7439547, EPI_ISL_7439558, EPI_ISL_7439622, EPI_ISL_7439646, EPI_ISL_7439650, EPI_ISL_7439657, EPI_ISL_7439722, EPI_ISL_7439730, EPI_ISL_7439754, EPI_ISL_7439781, EPI_ISL_7439806, EPI_ISL_7439827, EPI_ISL_7439832, EPI_ISL_7439902, EPI_ISL_7439935			
see above	IMD - MVZ Labor Martinsried	Robert Koch Institute	
EPI_ISL_7451030, EPI_ISL_7451040, EPI_ISL_7451054, EPI_ISL_7451096, EPI_ISL_7565411	INSA	Instituto Nacional de Saude (INSA)	Borges et al
EPI_ISL_7496734	Idaho Bureau of Laboratories	Idaho Bureau of Laboratories	"R. Beukelman; Aimee Ceniseros; Christian Loera; Christopher Ball"; Matthew Charles Burns; Robert L. Voermans
EPI_ISL_7544430	Ikhwezi CDC wc IKW	NHLS/UCT	Arash Iranzadeh; Bruna Galvao; Carolyn Williamson; Deelan Doolabh; Diana Hardie; Gert Marais; Innocent Mudau; Luicer Olubayo; Lynn Tyers; Marvin Hsiao; Nokuzola Mbhele; Rageema Joseph; Stephen Korsman
EPI_ISL_7660164, EPI_ISL_7660215, EPI_ISL_7660217	Illinois Department of Public Health	Illinois Department of Public Health - Chicago Lab	Ira Heimler; Joel Price; Vineet K. Dhiman
EPI_ISL_7381102	Indian Council of Medical Research-National Institute of Virology, Microbial Containment Complex	Indian Council of Medical Research-National Institute of Virology, Microbial Containment Complex	Pragya D. Yadav
EPI_ISL_7166400	Indira Gandhi Memorial Hospital	Indira Gandhi Memorial Hospital	D. Fathmath Nazla Rafeeq; Dr. Ibrahim Afzal; Mr. Ibrahim Nishan Ahmed; Ms. Aishath Shuhudha; Ms. Aminath Shazleena Abdul Rahman
EPI_ISL_7607424	Infectious Disease Diagnostics Laboratory at the Children's Hospital of Philadelphia	Planet Lab, Children's Hospital of Philadelphia	Ahmed M. Moustafa; Alex Arvanitis; Andries Feder; Azad Ahmed; Bhaswati Sen; Donald C. Hall; Joshua Chang Mell; Paul J. Planet; Rebecca M. Harris; Swetha Rajagopal; Will Dampier
EPI_ISL_7497749	Infinity Biologix	Centers for Disease Control and Prevention Division of Viral Diseases, Pathogen Discovery	Benjamin Rambo-Martin; Chirayu Goswami; Christian Bixby; Christopher Gulvick; Clinton Paden; Dakota Howard; Dhwani Batra; Duncan MacCannell; Erisa Sula; Jason Caravas; Jonathan Schultz; Kristine Lacek; Matthew Schmerer; Peter Cook; Robin Grimwood; Russ Hager; Scott Sammons; Shatavia Morrison; Tymeckia Kendall; Victoria Caban Figueroa; Yihe Wang; Yvette Unoarumhi
EPI_ISL_7405329	Inselspital Bern (Covid-Track)	Institute for Infectious Diseases, University of Bern	Alban Ramette; Christian Baumann; Cora Sägesser; Franziska Suter-Riniker; Loïc Borcard; Miguel A Terrazos Miani; Nicole Liechti; Pascal Bittel; Peter Keller; Sonja Gempeler; Stefan Neuenschwander; Stephen L Leib
EPI_ISL_7117396	Institut für Labormedizin Mikrobiologie und Hygiene	Robert Koch Institute	
EPI_ISL_6832737	Institute for Medical Virology Frankfurt	Institute for Medical Virology Frankfurt	Ciesek S.; Toptan T.
EPI_ISL_6959868, EPI_ISL_6959869, EPI_ISL_6959870, EPI_ISL_6959871, EPI_ISL_6959872, EPI_ISL_6959873, EPI_ISL_6959874, EPI_ISL_7479163, EPI_ISL_7479173, EPI_ISL_7479181, EPI_ISL_7479186, EPI_ISL_7479193, EPI_ISL_7479200, EPI_ISL_7479206			
see above	Institute for Medical Virology, Frankfurt	Institute for Medical Virology, Frankfurt	Ciesek S.; Toptan T.
EPI_ISL_7404462, EPI_ISL_7404463	Institute of Epidemiology, Disease Control and Research (IEDCR)	IEDCR-ideSHi Genomics Lab	Firdausi Qadri; Hassan Afrad; Manjur Hossain Khan; Omar Hamza; Tahmina Shirin
EPI_ISL_7507055	Institute of Medical Science, University of Tokyo	Center for Influenza and Respiratory Virus Research, National Institute of Infectious Diseases (NIID)	Emi Takashita; Hideka Miura; Seiichiro Fujisaki; Yoshihiro Kawaoka; Yuko Sakai-Tagawa
EPI_ISL_6825546, EPI_ISL_6902052, EPI_ISL_6902053	Institute of Virology, Department of Hygiene, Microbiology and Public Health at Innsbruck Medical University	Institute of Virology, Department of Hygiene, Microbiology and Public Health at Innsbruck Medical University	Andreas Aufschnaiter; Barbara Falkensammer; David Bante; Dorothee von Laer; Heribert Stoiber; Lukas Perro; Stephan Amstler; Wegene Borena
EPI_ISL_7550075	Instituto Adolfo Lutz - Regional de Rio Claro	Instituto Adolfo Lutz, Interdiciplinary Procedures Center, Strategic Laboratory	Claudio Tavares Sacchi; Karoline Rodrigues Campos
EPI_ISL_7632052	Invenimus AG	Institute of Medical Virology, University of Zurich	Alexandra Trkola; Annette Audigé; Catharine Aquino; Cyril Shah; Daniel Ehrsam; Gabriela Ziltener; Guido Bloemberg; Hubert Rehrauer; Isabel Stürmer; Joel Wirz; Jon Huder; Jürg Böni; Kevin Steiner; Maria Grünberg; Maryam Zaheri; Michael Huber; Riccarda Capaul; Stefan Schmutz; Verena Kufner; Weihong Qi
EPI_ISL_7571605, EPI_ISL_7571606, EPI_ISL_7571607, EPI_ISL_7571608, EPI_ISL_7571612, EPI_ISL_7571614	Iressef Genomics lab	IRESSEF	Abdou PADANE; Ambroise AHOUIDI; Aminata DIA; Aminata MBOUP; Astou Gaye GAYE; Barada CISSE; Birahim Piere NDIAYE; Cyrille Diedhiou; Diabou Diagne; Gora LO; Khadim GUEYE; Moustapha MBOW; Nafisatou LEYE; Ndeye Coumba Toure KANE; Papa Alassane DIAW; Samba Ndiour; Seni Ndiaye; Souleymane MBOUP; Yacine DIA
EPI_ISL_7160424	Johns Hopkins Hospital Department of Pathology	Johns Hopkins Hospital Department of Pathology	Amary Fall; C. Paul Morris; David Gaston; Heba H. Mostafa; Julie M. Norton; Matthew Schwartz; Michael Forman; Raghda Eldesouki
EPI_ISL_7605595, EPI_ISL_7605596, EPI_ISL_7605597, EPI_ISL_7605598, EPI_ISL_7605599, EPI_ISL_7605600, EPI_ISL_7605601, EPI_ISL_7605617, EPI_ISL_7605618, EPI_ISL_7605619, EPI_ISL_7605622, EPI_ISL_7605641, EPI_ISL_7605642, EPI_ISL_7605643, EPI_ISL_7605644, EPI_ISL_7605645, EPI_ISL_7605646, EPI_ISL_7605647, EPI_ISL_7605648, EPI_ISL_7605649, EPI_ISL_7605650, EPI_ISL_7605680, EPI_ISL_7605681, EPI_ISL_7605683, EPI_ISL_7605686, EPI_ISL_7605690, EPI_ISL_7605706, EPI_ISL_7605707, EPI_ISL_7605741			
see above	KIMBERLEY LABORATORY	National Institute for Communicable Diseases of the National Health Laboratory Service	Amoako DG; Bhiman JN; Everatt J; Ismail A; Mahlangu B; Mnguni A; Mohale T; Ntuli N; Scheepers C; Wolter N
EPI_ISL_6794907, EPI_ISL_6989250, EPI_ISL_7413964, EPI_ISL_7495278, EPI_ISL_7495279, EPI_ISL_7495280, EPI_ISL_7495281, EPI_ISL_7495282, EPI_ISL_7495283, EPI_ISL_7495284, EPI_ISL_7495285			
see above	KU Leuven, Rega Institute, Clinical and Epidemiological Virology	KU Leuven, Rega Institute, Clinical and Epidemiological Virology	Bert Vanmechelen; Casper Geenen; Emmanuel André; Guy Baele; Joan Marti-Carerras; Joren; Lize Cuypers; Piet Maes; Raymenants; Sarah Gorissen; Simon Dellicour; Tony Wawina-Bokalanga
EPI_ISL_7622272, EPI_ISL_7622286, EPI_ISL_7622287	Kaiser Permanente NW Reginal Lab	OHSU MM Lab	Amber Halse; Jeannine Lama; Xuan Qin; Yun Wu
EPI_ISL_7652567	Karolinska University Hospital Huddinge	Karolinska University Hospital	Annika Tiveljung Lindell; Henning Onsbring; Jan Albert; Karina Hentrich; Lynda Eneh; Maria Ropat; Martin Ekman; Natalija Gerasimcik; Robert Dyrdak; Sandra Broddesson; Shambhu Ganeshappa Aralaguppe; Tanja Normark; Tobias Allander; Valtteri Wirta; Zhibing Yun
EPI_ISL_7219990, EPI_ISL_7220437, EPI_ISL_7220444, EPI_ISL_7286284	Karolinska University Hospital Huddinge	Karolinska University Hospital Huddinge	Annika Tiveljung Lindell; Henning Onsbring; Jan Albert; Karina Hentrich; Lynda Eneh; Maria Ropat; Martin Ekman; Natalija Gerasimcik; Robert Dyrdak; Sandra Broddesson; Shambhu Ganeshappa Aralaguppe; Tanja Normark; Tobias Allander; Valtteri Wirta; Zhibing Yun
EPI_ISL_7652445	Karolinska University Hospital Solna	Karolinska University Hospital	Annika Tiveljung Lindell; Henning Onsbring; Jan Albert; Karina Hentrich; Lynda Eneh; Maria Ropat; Martin Ekman; Natalija Gerasimcik; Robert Dyrdak; Sandra Broddesson; Shambhu Ganeshappa Aralaguppe; Tanja Normark; Tobias Allander; Valtteri Wirta; Zhibing Yun
EPI_ISL_7381109, EPI_ISL_7381114, EPI_ISL_7457425, EPI_ISL_7457426	Karolinska University Hospital Solna	Karolinska University Hospital Huddinge	Annika Tiveljung Lindell; Henning Onsbring; Jan Albert; Karina Hentrich; Lynda Eneh; Maria Ropat; Martin Ekman; Natalija Gerasimcik; Robert Dyrdak; Sandra Broddesson; Shambhu Ganeshappa Aralaguppe; Tanja Normark; Tobias Allander; Valtteri Wirta; Zhibing Yun
EPI_ISL_7433816,	Klinikum Ernst von Bergmann	Robert Koch Institute	

EPI_ISL_7443687, EPI_ISL_7443713	gemeinnützige GmbH - stationärer Bereich		
EPI_ISL_7611152	LABORATOIRE BIOALLIANCE	CNR Virus des Infections Respiratoires - France SUD	Antonin Bal; Bruno Lina; Bruno Simon; Gregory Destras; Gwendolyne Burfin; Hadrien Regue; Laurence Josset; Martine Valette; Quentin Semanas
EPI_ISL_7224971	LACEN do Distrito Federal	Instituto Adolfo Lutz Strategic Laboratory	Claudio Tavares Sacchi; Karoline Rodrigues Campos; Marlon Benedito Nascimento Santos
EPI_ISL_7550076	LACEN do Distrito Federal	Instituto Adolfo Lutz, Interdiciplinary Procedures Center, Strategic Laboratory	Claudio Tavares Sacchi; Karoline Rodrigues Campos
EPI_ISL_7610177	LAM CERBALLIANCE	CNR Virus des Infections Respiratoires - France SUD	Antonin Bal; Bruno Lina; Bruno Simon; Gregory Destras; Gwendolyne Burfin; Hadrien Regue; Laurence Josset; Martine Valette; Quentin Semanas
EPI_ISL_6647956, EPI_ISL_6647957, EPI_ISL_6647958, EPI_ISL_6647959, EPI_ISL_6647960, EPI_ISL_6647961, EPI_ISL_6647962, EPI_ISL_6698792, EPI_ISL_6704863, EPI_ISL_6704864, EPI_ISL_6704865, EPI_ISL_6704866, EPI_ISL_6704867, EPI_ISL_6704868, EPI_ISL_6704869, EPI_ISL_6704870, EPI_ISL_6704871, EPI_ISL_6704872, EPI_ISL_6704873, EPI_ISL_6704874, EPI_ISL_6704875, EPI_ISL_6704876			
see above	LANCET LABORATORY	National Institute for Communicable Diseases of the National Health Laboratory Service	Amoako DG; Bhiman JN; Everatt J; Glass A; Ismail A; Mahlangu B; Mnguni A; Mohale T; Ntuli N; Scheepers C; Viana R; Wolter N
EPI_ISL_6901960, EPI_ISL_6901961, EPI_ISL_7473154	LATE - Laboratório de Técnicas Especiais - Hospital Israelita Albert Einstein	LATE - Laboratório de Técnicas Especiais - Hospital Israelita Albert Einstein	Alexandre Hideaki Takara; Ana Paula Moreira Salles; Anelisie da Silva Santos; Deyvid Amgarten; Erick Gustavo Dorlass; Fernanda de Mello Malta; João Renato Rebello Pinho; Luiz Vicente Rizzo; Marcio Anunciacao Menezes; Pedro Henrique Sebe Rodrigues; Raquel Riyuzo
EPI_ISL_7472277	LKO	Jessa	Severine Berden et al. on behalf of the Jessa_cmdLab
EPI_ISL_7456529	LSUHS Emerging Viral Threat Lab	LSUHS Emerging Viral Threat Laboratory	Adrian Almodovar; Alexander Mijalis; Andrew D. Yurochko; Christopher G. Kevil; Gregory L. Ware; Jennifer L. Carroll; Jeremy P. Kamil; John A. Vanchiere; Krista Queen; Maarten Van Diest; Rona S. Scott
EPI_ISL_7339434, EPI_ISL_7339435, EPI_ISL_7339436, EPI_ISL_7651330, EPI_ISL_7651331, EPI_ISL_7651332, EPI_ISL_7651333, EPI_ISL_7651334, EPI_ISL_7651335, EPI_ISL_7651336, EPI_ISL_7651337			
see above	Lab voor klinische biologie	Lab voor klinische biologie	Bruno Verhasselt; Hannelore Hamerlinck; Marija Janevska; May-Linh Truong
EPI_ISL_7589521	Labo Analyses Med	National Reference Center for Viruses of Respiratory Infections, Institut Pasteur, Paris	Angela Brisebarre; Camille Capel; Christophe Malabat; Corinne Maufrais; Etienne Simon-Lorière; Frédéric Lemoine; Julien Fumey; Louise Lefrançois; Marion Barbet; Maud Vanpeene; Méline Bizard; Philippe GIRARD; Slim El Khiari; Sylvie Behillil; Sylvie Van der Werf; Vincent Enouf
EPI_ISL_7437787	Labor Prof. Dr. G. Enders MVZ GbR	Robert Koch Institute	
EPI_ISL_7450035, EPI_ISL_7450728	Laboratoire BIORANCE	CHU Pontchaillou	DE TAYRAC Marie; DENOUAL Florent; ETCHEVERRY Amandine; FEBREAU Christine; GALIBERT Marie Dominique; GROLHIER Claire; JAGLINE Steven; PRONIER Charlotte; QUENET Benjamin; SASSI Mohamed; THIBAULT Vincent
EPI_ISL_6971091	Laboratoire analyse med	LABORIZON CENTRE BIOGROUP	HAGUENOER EVE; HOLSTEIN ANNE; JIMENEZ MELANIE; LEFLEUTER NICOLAS; POITIRON GREGOIRE
EPI_ISL_7309168	Laboratorio Central de Saude Publica do Rio Grande do Sul/Centro Estadual de Vigilancia em Saude	Centro de Desenvolvimento Cientifico e Tecnologico (CDCT)/Centro Estadual de Vigilancia em Saude	Alana Rossetti; Cláudia Maria Dornelles da Silva; Fernanda Marques Godinho; Larissa Vitoria da Silva; Ludmila Fiorenzano Baethgen; Miguel S Andrade.; Regina Bones Barcellos; Richard Steiner Salvato; Tatiana Schaffer Gregianini
EPI_ISL_7497691	Laboratory Corporation of America	Centers for Disease Control and Prevention Division of Viral Diseases, Pathogen Discovery	Amanda Douglas; Amanda Suchanek; Andrea Throop; Ayla Burns; Benjamin Rambo-Martin; Bobbi Croy; Brian Krueger; Brian Norvell; Christopher Gulvick; Christos Petropoulos; Clinton Paden; Craig Lukasik; Dakota Howard; Debbie Boles; Dhwani Batra; Duncan MacCannell; Eyad Almasri; Goran Stevovic; Howard Engler; Hrushikesh Deshmukh; Jake Humphrey; Jana Schroth; Jason Caravas; Joe Voshell; John Pruitt; Jonathan Meltzer; Jonathan Williams; Kimberly Wagner; Kristine Lacek; Lax Iyer; Lisa Pfefferle; Lyndon Tilson; Manoj Jain; Marcia Eisenberg; Mary Cristobal; Mary Williamson; Matthew Robinson; Matthew Schmerer; Michael Levandoski; Mike Sapeta; Mindy Nye; Minoo Agarwal; Mohan Kolli; Nuthawin Charoensri; Oren Cohen; Peter Cook; Prashant Gupta; Qian Zeng; Rama Ghatti; Scott Parker; Scott Ryan; Scott Sammons; Shatavia Morrison; Stanley Letovsky; Steven Ragan; Suresh Selvaraju; Susan Countryman; Susan Hicks; Suzanne Dale; Thomas Urban; Tim Kuphal; Tricia Zwiefelhofer; Tymeckia Kendall; Victoria Caban Figueroa; Vincent Drouillon; Yvette Unoarumhi
EPI_ISL_7567146	Laboratory of Clinical Microbiology, Virology and Bioemergencies, ASST Fatebenefratelli Sacco - Sacco Hospital	Laboratory of Clinical Microbiology, Virology and Bioemergencies, ASST Fatebenefratelli Sacco - Sacco Hospital	Alberto Rizzo; Fiorenza Bracchitta; Valeria Micheli
EPI_ISL_7220176	Laboratory of Clinical Virology Heraklion Crete	Laboratory of Clinical Virology Heraklion Crete	Alexandros Zafiropoulos; George Sourvinos
EPI_ISL_7651286, EPI_ISL_7651287, EPI_ISL_7651288, EPI_ISL_7651289	Laboratory of Molecular Biology and Cancer Immunology, Faculty of Sciences, Lebanese University	Microbial Pathogenomics Lab - LAU	Alissar Zaghlout; Bassam Badran; Fadi Abdel Sater; Georgi Merhi; Jad Koweyes; Nada Ghosn; Rawan Makki; Sima tokajian
EPI_ISL_7264143	Lahey Hospital	New England Biolabs	Abel, G.; B.W.; C.J.; Elfahal, M.; Flynn; Heim, K.; Karolides, M.; L. and Langhorst; Leger, P.; Michaels, L.; Pinet, K.; Skelton, T.; Sun
EPI_ISL_7543847, EPI_ISL_7543913, EPI_ISL_7543935, EPI_ISL_7543949, EPI_ISL_7543960, EPI_ISL_7544508, EPI_ISL_7544601, EPI_ISL_7544676, EPI_ISL_7544705			
see above	Lancet	NHLS/UCT	Arash Iranzadeh; Bruna Galvao; Carolyn Williamson; Deelan Doolabh; Diana Hardie; Gert Marais; Innocent Mudau; Luicer Olubayo; Lynn Tyers; Marvin Hsiao; Nokuzola Mbhele; Rageema Joseph; Stephen Korsman
EPI_ISL_6913991, EPI_ISL_6913992, EPI_ISL_6913993, EPI_ISL_6913994, EPI_ISL_6913995, EPI_ISL_6913996, EPI_ISL_6913997, EPI_ISL_6913998, EPI_ISL_6913999, EPI_ISL_6914000, EPI_ISL_6914001, EPI_ISL_6914002, EPI_ISL_6914003, EPI_ISL_6914004, EPI_ISL_6914005, EPI_ISL_6914006, EPI_ISL_6914007			
see above	Lancet Laboratories	National Institute for Communicable Diseases of the National Health Laboratory Service	Amoako DG; Bhiman JN; Everatt J; Ismail A; Mahlangu B; Mnguni A; Mohale T; Ntuli N; Scheepers C; Wolter N
EPI_ISL_7420297, EPI_ISL_7420298, EPI_ISL_7420371, EPI_ISL_7420381	Landesgesundheitsamt Baden-Württemberg	Robert Koch Institute	
EPI_ISL_7543863	Langa Clinic wc LAN	NHLS/UCT	Arash Iranzadeh; Bruna Galvao; Carolyn Williamson; Deelan Doolabh; Diana Hardie; Gert Marais; Innocent Mudau; Luicer Olubayo; Lynn Tyers; Marvin Hsiao; Nokuzola Mbhele; Rageema Joseph; Stephen Korsman
EPI_ISL_7620900, EPI_ISL_7621182, EPI_ISL_7621503, EPI_ISL_7621817	Laverty Pathology	NSW Health Pathology - Institute of Clinical Pathology and Medical Research; Westmead Hospital; University of Sydney	Arnott A.; Draper J.; Gall M.; Martinez E.; Rockett R.; Sintchenko V.; on behalf of ICPMR
EPI_ISL_7348417, EPI_ISL_7348427	Lifebrain Covid Labor GmbH	Lifebrain Covid Labor GmbH	Filip Sima
EPI_ISL_6821008, EPI_ISL_6916148, EPI_ISL_7023724, EPI_ISL_7144808, EPI_ISL_7144986, EPI_ISL_7148837, EPI_ISL_7187307, EPI_ISL_7200868, EPI_ISL_7290346, EPI_ISL_7293790, EPI_ISL_7293812, EPI_ISL_7293816, EPI_ISL_7293841, EPI_ISL_7293869, EPI_ISL_7293903, EPI_ISL_7294032, EPI_ISL_7294185, EPI_ISL_7296873, EPI_ISL_7301592, EPI_ISL_7301667, EPI_ISL_7305287, EPI_ISL_7344013, EPI_ISL_7344598, EPI_ISL_7344677, EPI_ISL_7346484, EPI_ISL_7351048, EPI_ISL_7351423, EPI_ISL_7351936, EPI_ISL_7353170, EPI_ISL_7382609, EPI_ISL_7383321, EPI_ISL_7385376, EPI_ISL_7389680, EPI_ISL_7392768, EPI_ISL_7397825, EPI_ISL_7484328, EPI_ISL_7488052, EPI_ISL_7490160, EPI_ISL_7491433, EPI_ISL_7491455, EPI_ISL_7491475, EPI_ISL_7492192, EPI_ISL_7492231, EPI_ISL_7511780, EPI_ISL_7514364, EPI_ISL_7514804, EPI_ISL_7515071, EPI_ISL_7515102, EPI_ISL_7515212, EPI_ISL_7515213, EPI_ISL_7515290, EPI_ISL_7515719, EPI_ISL_7516761, EPI_ISL_7535745, EPI_ISL_7535814, EPI_ISL_7535856, EPI_ISL_7535938, EPI_ISL_7536494, EPI_ISL_7536603, EPI_ISL_7537524, EPI_ISL_7538327, EPI_ISL_7539165, EPI_ISL_7539548, EPI_ISL_7539590, EPI_ISL_7539720, EPI_ISL_7541054, EPI_ISL_7575953, EPI_ISL_7577543, EPI_ISL_7578261, EPI_ISL_7578477, EPI_ISL_7578511, EPI_ISL_7579253, EPI_ISL_7624651, EPI_ISL_7624729, EPI_ISL_7624812, EPI_ISL_7624845, EPI_ISL_7624907, EPI_ISL_7624982, EPI_ISL_7625130, EPI_ISL_7625133, EPI_ISL_7625144, EPI_ISL_7625153, EPI_ISL_7625195, EPI_ISL_7625239, EPI_ISL_7625258, EPI_ISL_7625280, EPI_ISL_7625304, EPI_ISL_7625309, EPI_ISL_7625315, EPI_ISL_7625322, EPI_ISL_7625367, EPI_ISL_7625383, EPI_ISL_7625399, EPI_ISL_7625421, EPI_ISL_7625526, EPI_ISL_7625576, EPI_ISL_7625609, EPI_ISL_7625644, EPI_ISL_7625694, EPI_ISL_7625703, EPI_ISL_7627930, EPI_ISL_7627979, EPI_ISL_7627980, EPI_ISL_7628180, EPI_ISL_7628307, EPI_ISL_7628386, EPI_ISL_7628524, EPI_ISL_7628571, EPI_ISL_7628637, EPI_ISL_7628726, EPI_ISL_7628901, EPI_ISL_7628998, EPI_ISL_7629019, EPI_ISL_7629020, EPI_ISL_7629176, EPI_ISL_7629711, EPI_ISL_7629998, EPI_ISL_7630011, EPI_ISL_7630066, EPI_ISL_7630877, EPI_ISL_7630946, EPI_ISL_7630956, EPI_ISL_7630960, EPI_ISL_7630981, EPI_ISL_7630998, EPI_ISL_7631021, EPI_ISL_7631066, EPI_ISL_7631081, EPI_ISL_7631083, EPI_ISL_7631094, EPI_ISL_7631138, EPI_ISL_7631408, EPI_ISL_7631425, EPI_ISL_7631506, EPI_ISL_7632206, EPI_ISL_7632745, EPI_ISL_7632760, EPI_ISL_7634914, EPI_ISL_7635213, EPI_ISL_7635249, EPI_ISL_7635336, EPI_ISL_7635376, EPI_ISL_7635385, EPI_ISL_7635502, EPI_ISL_7635701, EPI_ISL_7635816, EPI_ISL_7653870, EPI_ISL_7653935, EPI_ISL_7654152, EPI_ISL_7654406, EPI_ISL_7654519, EPI_ISL_7654560, EPI_ISL_7654580			
see above	Lighthouse Lab in Alderley Park	Wellcome Sanger Institute for the COVID-19 Genomics UK (COG-UK) Consortium	Cordelia Langford; David K. Jackson; Dominic Kwiatkowski; Ewan Harrison; Ian Johnston; Jacquelyn Wynn; Jeffrey Barrett; John Sillitoe on behalf of the Wellcome Sanger Institute COVID-19 Surveillance Team; Mairead Hyland; Roberto Amato; Sonia Goncalves; The Lighthouse Lab in Alderley Park and Alex Alderton
EPI_ISL_6869226, EPI_ISL_6869363, EPI_ISL_6869407, EPI_ISL_6869556, EPI_ISL_6873612, EPI_ISL_6920174, EPI_ISL_6926451, EPI_ISL_6926743, EPI_ISL_6927893, EPI_ISL_7019951, EPI_ISL_7022011, EPI_ISL_7027157, EPI_ISL_7027809, EPI_ISL_7029582, EPI_ISL_7031141, EPI_ISL_7031412, EPI_ISL_7035922, EPI_ISL_7036426, EPI_ISL_7145006, EPI_ISL_7145516, EPI_ISL_7148141, EPI_ISL_7148719, EPI_ISL_7158402, EPI_ISL_7158414, EPI_ISL_7158423, EPI_ISL_7158488, EPI_ISL_7158498, EPI_ISL_7158548, EPI_ISL_7292889, EPI_ISL_7292960, EPI_ISL_7294550, EPI_ISL_7294585, EPI_ISL_7295180, EPI_ISL_7295212, EPI_ISL_7295689, EPI_ISL_7296032, EPI_ISL_7296139, EPI_ISL_7296254, EPI_ISL_7296303, EPI_ISL_7297042, EPI_ISL_7297075, EPI_ISL_7297120, EPI_ISL_7299420, EPI_ISL_7299535, EPI_ISL_7299536, EPI_ISL_7299709, EPI_ISL_7299880, EPI_ISL_7302122, EPI_ISL_7302177, EPI_ISL_7302222, EPI_ISL_7302501, EPI_ISL_7302554, EPI_ISL_7303186, EPI_ISL_7303590, EPI_ISL_7303781, EPI_ISL_7304114, EPI_ISL_7304964, EPI_ISL_7305445, EPI_ISL_7305675, EPI_ISL_7305684, EPI_ISL_7305734, EPI_ISL_7305755, EPI_ISL_7305800, EPI_ISL_7305876, EPI_ISL_7305903, EPI_ISL_7305934, EPI_ISL_7306117, EPI_ISL_7306238, EPI_ISL_7306338, EPI_ISL_7306343, EPI_ISL_7306399, EPI_ISL_7343337, EPI_ISL_7343938, EPI_ISL_7343985, EPI_ISL_7344167, EPI_ISL_7344174, EPI_ISL_7345819, EPI_ISL_7345863, EPI_ISL_7345932, EPI_ISL_7345966, EPI_ISL_7345975, EPI_ISL_7346136, EPI_ISL_7346249, EPI_ISL_7346862, EPI_ISL_7346896, EPI_ISL_7347059, EPI_ISL_7347482, EPI_ISL_7350891, EPI_ISL_7351023, EPI_ISL_7351093, EPI_ISL_7352907, EPI_ISL_7353351, EPI_ISL_7355546, EPI_ISL_7355957, EPI_ISL_7355992, EPI_ISL_7356061, EPI_ISL_7356171, EPI_ISL_7356256, EPI_ISL_7384310, EPI_ISL_7389585, EPI_ISL_7392688, EPI_ISL_7392706, EPI_ISL_7394189, EPI_ISL_7394919, EPI_ISL_7397103, EPI_ISL_7397308, EPI_ISL_7397319, EPI_ISL_7397480, EPI_ISL_7510398, EPI_ISL_7510491, EPI_ISL_7510550, EPI_ISL_7510572, EPI_ISL_7510800, EPI_ISL_7510886, EPI_ISL_7510922, EPI_ISL_7510928, EPI_ISL_7510978, EPI_ISL_7511143, EPI_ISL_7511154, EPI_ISL_7511233, EPI_ISL_7511305, EPI_ISL_7511455, EPI_ISL_7511587, EPI_ISL_7511662, EPI_ISL_7511677, EPI_ISL_7511702, EPI_ISL_7511820, EPI_ISL_7511832, EPI_ISL_7511896, EPI_ISL_7511986, EPI_ISL_7517098, EPI_ISL_7517101, EPI_ISL_7517216, EPI_ISL_7518683, EPI_ISL_7519628, EPI_ISL_7520531, EPI_ISL_7521048, EPI_ISL_7522619, EPI_ISL_7522673, EPI_ISL_7531008, EPI_ISL_7531228, EPI_ISL_7531322, EPI_ISL_7532295, EPI_ISL_7535753, EPI_ISL_7536021, EPI_ISL_7536166, EPI_ISL_7536246, EPI_ISL_7536342, EPI_ISL_7536453, EPI_ISL_7536705, EPI_ISL_7536927, EPI_ISL_7557186, EPI_ISL_7558117, EPI_ISL_7558170, EPI_ISL_7558350, EPI_ISL_7558383, EPI_ISL_7571990, EPI_ISL_7572056, EPI_ISL_7572178, EPI_ISL_7572425, EPI_ISL_7572906, EPI_ISL_7572919, EPI_ISL_7572943, EPI_ISL_7572961, EPI_ISL_7572962, EPI_ISL_7572986, EPI_ISL_7573003, EPI_ISL_7573055, EPI_ISL_7573101, EPI_ISL_7573102, EPI_ISL_7573151, EPI_ISL_7573158, EPI_ISL_7573256, EPI_ISL_7573278, EPI_ISL_7573306, EPI_ISL_7573335, EPI_ISL_7573358, EPI_ISL_7573377, EPI_ISL_7573415, EPI_ISL_7573450, EPI_ISL_7573467, EPI_ISL_7573468, EPI_ISL_7573477, EPI_ISL_7573884, EPI_ISL_7573917, EPI_ISL_7573931, EPI_ISL_7574004, EPI_ISL_7574007, EPI_ISL_7574049, EPI_ISL_7574070, EPI_ISL_7574201, EPI_ISL_7574281, EPI_ISL_7575331, EPI_ISL_7575392, EPI_ISL_7575420, EPI_ISL_7575480, EPI_ISL_7575510, EPI_ISL_7575511, EPI_ISL_7575543, EPI_ISL_7575550, EPI_ISL_7575586, EPI_ISL_7575588, EPI_ISL_7575633, EPI_ISL_7575684, EPI_ISL_7575753, EPI_ISL_7575773, EPI_ISL_7578677, EPI_ISL_7578871, EPI_ISL_7579130, EPI_ISL_7582829, EPI_ISL_7585552, EPI_ISL_7585596, EPI_ISL_7585650, EPI_ISL_7585720, EPI_ISL_7585731, EPI_ISL_7585732, EPI_ISL_7585734, EPI_ISL_7585741, EPI_ISL_7585789, EPI_ISL_7585847, EPI_ISL_7585936, EPI_ISL_7585968, EPI_ISL_7585991, EPI_ISL_7585998, EPI_ISL_7586089, EPI_ISL_7586090, EPI_ISL_7586102, EPI_ISL_7586111, EPI_ISL_7586288, EPI_ISL_7586290, EPI_ISL_7586324, EPI_ISL_7586362, EPI_ISL_7586434, EPI_ISL_7586500, EPI_ISL_7586596, EPI_ISL_7624515, EPI_ISL_7624601, EPI_ISL_7624610, EPI_ISL_7624706, EPI_ISL_7624769, EPI_ISL_7624865, EPI_ISL_7624949, EPI_ISL_7624976, EPI_ISL_7624991, EPI_ISL_7625874, EPI_ISL_7625890, EPI_ISL_7625957, EPI_ISL_7626058, EPI_ISL_7626063, EPI_ISL_7626092, EPI_ISL_7626095, EPI_ISL_7626105, EPI_ISL_7626118, EPI_ISL_7626122, EPI_ISL_7626123, EPI_ISL_7626132, EPI_ISL_7626147, EPI_ISL_7626176, EPI_ISL_7626207, EPI_ISL_7626229, EPI_ISL_7626304, EPI_ISL_7626319, EPI_ISL_7626327, EPI_ISL_7626440, EPI_ISL_7626502, EPI_ISL_7626530, EPI_ISL_7627296, EPI_ISL_7627311, EPI_ISL_7627327, EPI_ISL_7627334, EPI_ISL_7627354, EPI_ISL_7627355, EPI_ISL_7627363, EPI_ISL_7627416, EPI_ISL_7627492, EPI_ISL_7627503, EPI_ISL_7627529, EPI_ISL_7627668, EPI_ISL_7627706, EPI_ISL_7627760, EPI_ISL_7627771, EPI_ISL_7627780, EPI_ISL_7627787, EPI_ISL_7627790, EPI_ISL_7627815, EPI_ISL_7627838, EPI_ISL_7627841, EPI_ISL_7627868, EPI_ISL_7627879, EPI_ISL_7627885, EPI_ISL_7627891, EPI_ISL_7627937, EPI_ISL_7627940, EPI_ISL_7627973, EPI_ISL_7627975, EPI_ISL_7627995, EPI_ISL_7627997, EPI_ISL_7628000, EPI_ISL_7628008, EPI_ISL_7628050, EPI_ISL_7628053, EPI_ISL_7628102, EPI_ISL_7628111, EPI_ISL_7628142, EPI_ISL_7628157, EPI_ISL_7628199, EPI_ISL_7628213, EPI_ISL_7628232, EPI_ISL_7628255, EPI_ISL_7628287, EPI_ISL_7628343, EPI_ISL_7628415, EPI_ISL_7628424, EPI_ISL_7628425, EPI_ISL_7628466, EPI_ISL_7628522, EPI_ISL_7628530, EPI_ISL_7628549, EPI_ISL_7628550, EPI_ISL_7628576, EPI_ISL_7628577, EPI_ISL_7628591, EPI_ISL_7628633, EPI_ISL_7628674, EPI_ISL_7628709, EPI_ISL_7628780, EPI_ISL_7628795, EPI_ISL_7628817, EPI_ISL_7628857, EPI_ISL_7628910, EPI_ISL_7628932, EPI_ISL_7628934, EPI_ISL_7628944, EPI_ISL_7628971, EPI_ISL_7629002, EPI_ISL_7629073, EPI_ISL_7629085, EPI_ISL_7629087, EPI_ISL_7629116, EPI_ISL_7629126, EPI_ISL_7629149, EPI_ISL_7630217, EPI_ISL_7630247, EPI_ISL_7630295, EPI_ISL_7630305, EPI_ISL_7630312, EPI_ISL_7630319, EPI_ISL_7630320, EPI_ISL_7630324, EPI_ISL_7630338, EPI_ISL_7630381, EPI_ISL_7630413, EPI_ISL_7630432, EPI_ISL_7630438, EPI_ISL_7630464, EPI_ISL_7630528, EPI_ISL_7630557, EPI_ISL_7630599, EPI_ISL_7630626, EPI_ISL_7630652, EPI_ISL_7630661, EPI_ISL_7630693, EPI_ISL_7630702, EPI_ISL_7630727, EPI_ISL_7631177, EPI_ISL_7631374, EPI_ISL_7631420, EPI_ISL_7631438, EPI_ISL_7631459, EPI_ISL_7631599, EPI_ISL_7631618, EPI_ISL_7631728, EPI_ISL_7631904, EPI_ISL_7632067, EPI_ISL_7632092, EPI_ISL_7632160, EPI_ISL_7632168, EPI_ISL_7632318, EPI_ISL_7632335, EPI_ISL_7632471, EPI_ISL_7632553, EPI_ISL_7632572, EPI_ISL_7632603, EPI_ISL_7632604, EPI_ISL_7632613, EPI_ISL_7632655, EPI_ISL_7632690, EPI_ISL_7633109, EPI_ISL_7633127, EPI_ISL_7633138, EPI_ISL_7633227, EPI_ISL_7633252, EPI_ISL_7633276, EPI_ISL_7633321, EPI_ISL_7633528, EPI_ISL_7633653, EPI_ISL_7633655, EPI_ISL_7633676, EPI_ISL_7633702, EPI_ISL_7633722, EPI_ISL_7634899, EPI_ISL_7634958, EPI_ISL_7634967, EPI_ISL_7634976, EPI_ISL_7634991, EPI_ISL_7635014, EPI_ISL_7635032, EPI_ISL_7635055, EPI_ISL_7635110, EPI_ISL_7635183, EPI_ISL_7635196, EPI_ISL_7635283, EPI_ISL_7635319, EPI_ISL_7635341, EPI_ISL_7635403, EPI_ISL_7635473, EPI_ISL_7635581, EPI_ISL_7635641, EPI_ISL_7635681, EPI_ISL_7635744, EPI_ISL_7635799, EPI_ISL_7653898, EPI_ISL_7653931, EPI_ISL_7653937, EPI_ISL_7653983, EPI_ISL_7654016, EPI_ISL_7654025, EPI_ISL_7654045, EPI_ISL_7654064, EPI_ISL_7654164, EPI_ISL_7654268, EPI_ISL_7654423, EPI_ISL_7654483, EPI_ISL_7654769, EPI_ISL_7655039, EPI_ISL_7655944, EPI_ISL_7655964, EPI_ISL_7655968, EPI_ISL_7655981, EPI_ISL_7655993, EPI_ISL_7656062, EPI_ISL_7656064, EPI_ISL_7656083, EPI_ISL_7656095, EPI_ISL_7656102, EPI_ISL_7656117, EPI_ISL_7656121, EPI_ISL_7656137, EPI_ISL_7656165, EPI_ISL_7656184, EPI_ISL_7656209, EPI_ISL_7656213, EPI_ISL_7656233, EPI_ISL_7656245, EPI_ISL_7656251, EPI_ISL_7656287, EPI_ISL_7656300, EPI_ISL_7656317, EPI_ISL_7656322, EPI_ISL_7656327			

see above	Lighthouse Lab in Glasgow	Wellcome Sanger Institute for the COVID-19 Genomics UK (COG-UK) Consortium	Anna Dominiczak and Alex Alderton; Carol Clugston; Cordelia Langford; David Gray; David K. Jackson; Dominic Kwiatkowski; Ewan Harrison; Harper VanSteenhouse; Ian Johnston; Jeffrey Barrett; John Sillitoe on behalf of the Wellcome Sanger Institute COVID-19 Surveillance Team; Roberto Amato; Sonia Goncalves; Yumi Kasai
EPI_ISL_6818284, EPI_ISL_6833884, EPI_ISL_6834467, EPI_ISL_6915964, EPI_ISL_6919027, EPI_ISL_6920616, EPI_ISL_6923186, EPI_ISL_7022755, EPI_ISL_7023187, EPI_ISL_7023265, EPI_ISL_7023667, EPI_ISL_7023675, EPI_ISL_7023683, EPI_ISL_7023726, EPI_ISL_7023789, EPI_ISL_7023867, EPI_ISL_7024334, EPI_ISL_7024438, EPI_ISL_7024495, EPI_ISL_7024659, EPI_ISL_7024687, EPI_ISL_7024839, EPI_ISL_7024991, EPI_ISL_7025373, EPI_ISL_7026085, EPI_ISL_7026396, EPI_ISL_7026536, EPI_ISL_7026616, EPI_ISL_7026695, EPI_ISL_7026716, EPI_ISL_7026777, EPI_ISL_7026952, EPI_ISL_7028268, EPI_ISL_7028487, EPI_ISL_7029353, EPI_ISL_7033208, EPI_ISL_7037264, EPI_ISL_7143464, EPI_ISL_7146338, EPI_ISL_7146517, EPI_ISL_7146524, EPI_ISL_7146933, EPI_ISL_7147199, EPI_ISL_7157375, EPI_ISL_7157554, EPI_ISL_7181721, EPI_ISL_7182128, EPI_ISL_7182133, EPI_ISL_7182156, EPI_ISL_7185699, EPI_ISL_7185720, EPI_ISL_7185763, EPI_ISL_7185784, EPI_ISL_7185838, EPI_ISL_7185864, EPI_ISL_7185866, EPI_ISL_7187248, EPI_ISL_7187503, EPI_ISL_7188445, EPI_ISL_7188816, EPI_ISL_7188897, EPI_ISL_7188957, EPI_ISL_7189046, EPI_ISL_7189048, EPI_ISL_7189067, EPI_ISL_7189073, EPI_ISL_7196773, EPI_ISL_7197261, EPI_ISL_7197402, EPI_ISL_7197454, EPI_ISL_7198739, EPI_ISL_7198774, EPI_ISL_7198902, EPI_ISL_7199159, EPI_ISL_7202939, EPI_ISL_7202951, EPI_ISL_7202968, EPI_ISL_7202977, EPI_ISL_7202998, EPI_ISL_7203007, EPI_ISL_7203016, EPI_ISL_7203017, EPI_ISL_7203032, EPI_ISL_7203085, EPI_ISL_7203096, EPI_ISL_7203100, EPI_ISL_7203105, EPI_ISL_7203132, EPI_ISL_7203159, EPI_ISL_7203161, EPI_ISL_7203182, EPI_ISL_7203185, EPI_ISL_7203202, EPI_ISL_7203249, EPI_ISL_7203710, EPI_ISL_7203762, EPI_ISL_7203765, EPI_ISL_7203936, EPI_ISL_7204224, EPI_ISL_7293149, EPI_ISL_7293182, EPI_ISL_7293573, EPI_ISL_7293699, EPI_ISL_7293701, EPI_ISL_7293733, EPI_ISL_7293785, EPI_ISL_7296211, EPI_ISL_7297545, EPI_ISL_7297579, EPI_ISL_7300159, EPI_ISL_7300279, EPI_ISL_7300286, EPI_ISL_7300537, EPI_ISL_7300658, EPI_ISL_7300878, EPI_ISL_7300889, EPI_ISL_7300915, EPI_ISL_7300986, EPI_ISL_7301005, EPI_ISL_7301013, EPI_ISL_7301015, EPI_ISL_7301165, EPI_ISL_7301209, EPI_ISL_7301266, EPI_ISL_7301307, EPI_ISL_7301521, EPI_ISL_7301561, EPI_ISL_7301876, EPI_ISL_7304476, EPI_ISL_7304564, EPI_ISL_7304690, EPI_ISL_7304994, EPI_ISL_7305027, EPI_ISL_7305176, EPI_ISL_7340046, EPI_ISL_7340118, EPI_ISL_7340964, EPI_ISL_7341048, EPI_ISL_7344931, EPI_ISL_7345012, EPI_ISL_7345138, EPI_ISL_7345164, EPI_ISL_7346326, EPI_ISL_7346393, EPI_ISL_7346410, EPI_ISL_7346479, EPI_ISL_7346521, EPI_ISL_7346666, EPI_ISL_7346704, EPI_ISL_7346758, EPI_ISL_7346809, EPI_ISL_7346816, EPI_ISL_7346835, EPI_ISL_7347737, EPI_ISL_7347952, EPI_ISL_7347992, EPI_ISL_7349648, EPI_ISL_7349666, EPI_ISL_7349764, EPI_ISL_7349797, EPI_ISL_7349804, EPI_ISL_7350042, EPI_ISL_7350201, EPI_ISL_7350318, EPI_ISL_7350332, EPI_ISL_7350365, EPI_ISL_7350520, EPI_ISL_7350526, EPI_ISL_7350556, EPI_ISL_7350583, EPI_ISL_7353443, EPI_ISL_7353487, EPI_ISL_7353795, EPI_ISL_7353847, EPI_ISL_7353853, EPI_ISL_7353856, EPI_ISL_7353981, EPI_ISL_7354003, EPI_ISL_7354110, EPI_ISL_7354518, EPI_ISL_7354594, EPI_ISL_7354762, EPI_ISL_7354795, EPI_ISL_7354941, EPI_ISL_7354957, EPI_ISL_7354991, EPI_ISL_7355006, EPI_ISL_7355123, EPI_ISL_7381834, EPI_ISL_7381856, EPI_ISL_7381881, EPI_ISL_7382005, EPI_ISL_7382097, EPI_ISL_7382103, EPI_ISL_7382156, EPI_ISL_7382200, EPI_ISL_7382215, EPI_ISL_7382254, EPI_ISL_7382416, EPI_ISL_7383376, EPI_ISL_7383676, EPI_ISL_7383683, EPI_ISL_7383685, EPI_ISL_7383740, EPI_ISL_7383773, EPI_ISL_7383781, EPI_ISL_7383783, EPI_ISL_7383798, EPI_ISL_7383852, EPI_ISL_7383856, EPI_ISL_7383863, EPI_ISL_7383885, EPI_ISL_7383921, EPI_ISL_7383922, EPI_ISL_7383936, EPI_ISL_7383941, EPI_ISL_7383945, EPI_ISL_7383954, EPI_ISL_7383977, EPI_ISL_7383978, EPI_ISL_7383980, EPI_ISL_7384190, EPI_ISL_7384545, EPI_ISL_7384619, EPI_ISL_7385445, EPI_ISL_7385606, EPI_ISL_7386026, EPI_ISL_7386052, EPI_ISL_7386325, EPI_ISL_7386397, EPI_ISL_7390306, EPI_ISL_7390618, EPI_ISL_7391659, EPI_ISL_7391668, EPI_ISL_7391823, EPI_ISL_7393627, EPI_ISL_7393737, EPI_ISL_7393800, EPI_ISL_7393853, EPI_ISL_7393873, EPI_ISL_7395502, EPI_ISL_7395791, EPI_ISL_7395797, EPI_ISL_7395849, EPI_ISL_7395905, EPI_ISL_7396016, EPI_ISL_7396038, EPI_ISL_7396042, EPI_ISL_7396056, EPI_ISL_7396060, EPI_ISL_7396114, EPI_ISL_7396120, EPI_ISL_7396167, EPI_ISL_7396207, EPI_ISL_7396238, EPI_ISL_7396262, EPI_ISL_7396271, EPI_ISL_7396409, EPI_ISL_7396415, EPI_ISL_7396446, EPI_ISL_7396483, EPI_ISL_7396525, EPI_ISL_7396533, EPI_ISL_7396579, EPI_ISL_7396628, EPI_ISL_7396640, EPI_ISL_7396987, EPI_ISL_7398089, EPI_ISL_7398228, EPI_ISL_7398295, EPI_ISL_7398393, EPI_ISL_7478708, EPI_ISL_7478734, EPI_ISL_7478799, EPI_ISL_7478827, EPI_ISL_7479102, EPI_ISL_7479225, EPI_ISL_7479311, EPI_ISL_7479350, EPI_ISL_7479729, EPI_ISL_7480249, EPI_ISL_7480340, EPI_ISL_7480459, EPI_ISL_7480563, EPI_ISL_7480779, EPI_ISL_7480983, EPI_ISL_7481103, EPI_ISL_7481888, EPI_ISL_7483648, EPI_ISL_7483732, EPI_ISL_7483778, EPI_ISL_7483853, EPI_ISL_7484047, EPI_ISL_7484394, EPI_ISL_7484568, EPI_ISL_7484575, EPI_ISL_7484580, EPI_ISL_7484583, EPI_ISL_7484584, EPI_ISL_7484632, EPI_ISL_7484936, EPI_ISL_7485137, EPI_ISL_7485159, EPI_ISL_7485300, EPI_ISL_7485391, EPI_ISL_7485734, EPI_ISL_7485763, EPI_ISL_7485880, EPI_ISL_7485890, EPI_ISL_7485899, EPI_ISL_7485918, EPI_ISL_7486077, EPI_ISL_7486120, EPI_ISL_7486163, EPI_ISL_7486373, EPI_ISL_7486447, EPI_ISL_7486481, EPI_ISL_7486517, EPI_ISL_7486635, EPI_ISL_7486819, EPI_ISL_7486971, EPI_ISL_7487426, EPI_ISL_7487488, EPI_ISL_7487790, EPI_ISL_7487954, EPI_ISL_7488141, EPI_ISL_7488200, EPI_ISL_7488279, EPI_ISL_7489187, EPI_ISL_7489326, EPI_ISL_7489389, EPI_ISL_7489446, EPI_ISL_7489786, EPI_ISL_7489819, EPI_ISL_7489953, EPI_ISL_7489980, EPI_ISL_7490259, EPI_ISL_7490487, EPI_ISL_7490584, EPI_ISL_7490883, EPI_ISL_7490957, EPI_ISL_7509888, EPI_ISL_7509901, EPI_ISL_7509939, EPI_ISL_7510081, EPI_ISL_7510216, EPI_ISL_7510241, EPI_ISL_7511995, EPI_ISL_7512017, EPI_ISL_7512047, EPI_ISL_7512058, EPI_ISL_7512077, EPI_ISL_7512119, EPI_ISL_7512155, EPI_ISL_7512175, EPI_ISL_7512265, EPI_ISL_7512279, EPI_ISL_7512284, EPI_ISL_7512302, EPI_ISL_7512321, EPI_ISL_7512475, EPI_ISL_7512549, EPI_ISL_7512707, EPI_ISL_7512966, EPI_ISL_7513072, EPI_ISL_7513131, EPI_ISL_7513234, EPI_ISL_7514854, EPI_ISL_7515519, EPI_ISL_7515566, EPI_ISL_7515634, EPI_ISL_7515973, EPI_ISL_7516148, EPI_ISL_7517004, EPI_ISL_7519115, EPI_ISL_7520174, EPI_ISL_7520334, EPI_ISL_7520362, EPI_ISL_7520998, EPI_ISL_7522351, EPI_ISL_7522461, EPI_ISL_7523110, EPI_ISL_7523203, EPI_ISL_7523364, EPI_ISL_7523595, EPI_ISL_7524106, EPI_ISL_7524864, EPI_ISL_7525422, EPI_ISL_7526214, EPI_ISL_7526231, EPI_ISL_7526276, EPI_ISL_7526372, EPI_ISL_7526379, EPI_ISL_7526426, EPI_ISL_7526472, EPI_ISL_7526476, EPI_ISL_7526571, EPI_ISL_7526583, EPI_ISL_7526592, EPI_ISL_7526597, EPI_ISL_7526604, EPI_ISL_7526609, EPI_ISL_7526665, EPI_ISL_7526690, EPI_ISL_7526766, EPI_ISL_7526796, EPI_ISL_7526806, EPI_ISL_7526823, EPI_ISL_7526841, EPI_ISL_7526878, EPI_ISL_7526961, EPI_ISL_7526985, EPI_ISL_7526997, EPI_ISL_7527046, EPI_ISL_7527101, EPI_ISL_7527267, EPI_ISL_7527283, EPI_ISL_7527298, EPI_ISL_7527348, EPI_ISL_7527381, EPI_ISL_7527452, EPI_ISL_7528184, EPI_ISL_7528477, EPI_ISL_7528569, EPI_ISL_7528699, EPI_ISL_7528999, EPI_ISL_7529344, EPI_ISL_7529479, EPI_ISL_7529568, EPI_ISL_7529705, EPI_ISL_7529714, EPI_ISL_7529749, EPI_ISL_7529754, EPI_ISL_7530006, EPI_ISL_7530158, EPI_ISL_7530274, EPI_ISL_7530290, EPI_ISL_7530610, EPI_ISL_7532349, EPI_ISL_7532487, EPI_ISL_7532636, EPI_ISL_7532667, EPI_ISL_7533126, EPI_ISL_7533558, EPI_ISL_7533573, EPI_ISL_7535208, EPI_ISL_7535261, EPI_ISL_7535308, EPI_ISL_7535330, EPI_ISL_7535331, EPI_ISL_7535381, EPI_ISL_7535476, EPI_ISL_7535510, EPI_ISL_7535600, EPI_ISL_7535650, EPI_ISL_7536373, EPI_ISL_7536571, EPI_ISL_7536608, EPI_ISL_7536664, EPI_ISL_7536712, EPI_ISL_7536778, EPI_ISL_7536955, EPI_ISL_7537157, EPI_ISL_7537194, EPI_ISL_7537382, EPI_ISL_7537419, EPI_ISL_7537698, EPI_ISL_7537754, EPI_ISL_7537774, EPI_ISL_7537864, EPI_ISL_7538259, EPI_ISL_7538311, EPI_ISL_7538654, EPI_ISL_7538855, EPI_ISL_7539129, EPI_ISL_7539442, EPI_ISL_7541246, EPI_ISL_7541250, EPI_ISL_7541383, EPI_ISL_7541410, EPI_ISL_7541455, EPI_ISL_7541551, EPI_ISL_7541700, EPI_ISL_7541743, EPI_ISL_7541804, EPI_ISL_7541826, EPI_ISL_7541859, EPI_ISL_7541865, EPI_ISL_7541886, EPI_ISL_7541892, EPI_ISL_7541941, EPI_ISL_7541961, EPI_ISL_7541994, EPI_ISL_7553144, EPI_ISL_7553149, EPI_ISL_7553161, EPI_ISL_7553192, EPI_ISL_7553197, EPI_ISL_7553204, EPI_ISL_7553212, EPI_ISL_7553248, EPI_ISL_7553287, EPI_ISL_7553310, EPI_ISL_7553311, EPI_ISL_7553332, EPI_ISL_7553333, EPI_ISL_7553339, EPI_ISL_7553392, EPI_ISL_7553477, EPI_ISL_7553485, EPI_ISL_7553491, EPI_ISL_7553529, EPI_ISL_7553567, EPI_ISL_7553579, EPI_ISL_7553585, EPI_ISL_7553618, EPI_ISL_7553651, EPI_ISL_7553666, EPI_ISL_7553670, EPI_ISL_7553711, EPI_ISL_7553748, EPI_ISL_7553762, EPI_ISL_7553833, EPI_ISL_7553873, EPI_ISL_7553888, EPI_ISL_7553903, EPI_ISL_7553907, EPI_ISL_7553927, EPI_ISL_7553934, EPI_ISL_7553963, EPI_ISL_7553964, EPI_ISL_7553983, EPI_ISL_7553987, EPI_ISL_7554005, EPI_ISL_7554013, EPI_ISL_7554019, EPI_ISL_7554096, EPI_ISL_7554137, EPI_ISL_7554171, EPI_ISL_7554248, EPI_ISL_7554281, EPI_ISL_7554321, EPI_ISL_7554346, EPI_ISL_7554445, EPI_ISL_7554476, EPI_ISL_7554489, EPI_ISL_7554490, EPI_ISL_7554516, EPI_ISL_7554550, EPI_ISL_7554560, EPI_ISL_7554680, EPI_ISL_7554690, EPI_ISL_7554699, EPI_ISL_7554703, EPI_ISL_7554783, EPI_ISL_7554850, EPI_ISL_7554872, EPI_ISL_7554949, EPI_ISL_7554973, EPI_ISL_7554995, EPI_ISL_7555045, EPI_ISL_7555072, EPI_ISL_7555086, EPI_ISL_7555141, EPI_ISL_7555150, EPI_ISL_7555163, EPI_ISL_7555176, EPI_ISL_7555186, EPI_ISL_7555190, EPI_ISL_7555191, EPI_ISL_7555195, EPI_ISL_7555212, EPI_ISL_7555216, EPI_ISL_7555273, EPI_ISL_7555291, EPI_ISL_7555306, EPI_ISL_7555380, EPI_ISL_7555396, EPI_ISL_7555470, EPI_ISL_7555477, EPI_ISL_7555530, EPI_ISL_7555535, EPI_ISL_7555659, EPI_ISL_7555700, EPI_ISL_7555721, EPI_ISL_7555744, EPI_ISL_7555758, EPI_ISL_7555774, EPI_ISL_7555793, EPI_ISL_7555851, EPI_ISL_7555885, EPI_ISL_7555991, EPI_ISL_7556003, EPI_ISL_7556033, EPI_ISL_7556161, EPI_ISL_7556164, EPI_ISL_7556207, EPI_ISL_7556299, EPI_ISL_7556333, EPI_ISL_7556340, EPI_ISL_7556345, EPI_ISL_7556347, EPI_ISL_7556440, EPI_ISL_7556446, EPI_ISL_7556454, EPI_ISL_7556458, EPI_ISL_7556547, EPI_ISL_7556562, EPI_ISL_7556583, EPI_ISL_7556612, EPI_ISL_7556832, EPI_ISL_7556878, EPI_ISL_7556879, EPI_ISL_7556883, EPI_ISL_7556891, EPI_ISL_7556899, EPI_ISL_7556905, EPI_ISL_7556979, EPI_ISL_7557010, EPI_ISL_7557022, EPI_ISL_7557048, EPI_ISL_7557104, EPI_ISL_7557126, EPI_ISL_7557152, EPI_ISL_7557167, EPI_ISL_7557302, EPI_ISL_7557304, EPI_ISL_7557331, EPI_ISL_7557391, EPI_ISL_7557422, EPI_ISL_7557463, EPI_ISL_7557476, EPI_ISL_7557485, EPI_ISL_7557490, EPI_ISL_7557510, EPI_ISL_7557520, EPI_ISL_7557522, EPI_ISL_7557536, EPI_ISL_7557542, EPI_ISL_7557546, EPI_ISL_7557555, EPI_ISL_7557558, EPI_ISL_7557594, EPI_ISL_7557630, EPI_ISL_7557742, EPI_ISL_7557755, EPI_ISL_7557757, EPI_ISL_7557788, EPI_ISL_7557793, EPI_ISL_7557821, EPI_ISL_7557823, EPI_ISL_7557844, EPI_ISL_7557974, EPI_ISL_7558442, EPI_ISL_7558468, EPI_ISL_7558493, EPI_ISL_7558519, EPI_ISL_7558533, EPI_ISL_7558555, EPI_ISL_7558619, EPI_ISL_7558638, EPI_ISL_7558693, EPI_ISL_7558721, EPI_ISL_7558737, EPI_ISL_7558768, EPI_ISL_7558795, EPI_ISL_7558844, EPI_ISL_7558848, EPI_ISL_7558852, EPI_ISL_7558893, EPI_ISL_7558908, EPI_ISL_7558926, EPI_ISL_7558929, EPI_ISL_7558973, EPI_ISL_7558997, EPI_ISL_7559064, EPI_ISL_7559105, EPI_ISL_7559107, EPI_ISL_7559111, EPI_ISL_7559169, EPI_ISL_7559234, EPI_ISL_7559254, EPI_ISL_7559289, EPI_ISL_7559301, EPI_ISL_7559319, EPI_ISL_7559331, EPI_ISL_7559333, EPI_ISL_7559345, EPI_ISL_7559382, EPI_ISL_7559401, EPI_ISL_7559415, EPI_ISL_7559416, EPI_ISL_7559426, EPI_ISL_7559448, EPI_ISL_7559467, EPI_ISL_7559481, EPI_ISL_7559482, EPI_ISL_7559516, EPI_ISL_7559535, EPI_ISL_7559692, EPI_ISL_7559715, EPI_ISL_7559728, EPI_ISL_7559782, EPI_ISL_7559931, EPI_ISL_7559966, EPI_ISL_7559980, EPI_ISL_7559987, EPI_ISL_7560016, EPI_ISL_7560039, EPI_ISL_7560044, EPI_ISL_7560085, EPI_ISL_7560095, EPI_ISL_7560099, EPI_ISL_7560109, EPI_ISL_7560111, EPI_ISL_7560125, EPI_ISL_7560131, EPI_ISL_7560137, EPI_ISL_7560156, EPI_ISL_7560160, EPI_ISL_7560165, EPI_ISL_7560175, EPI_ISL_7560179, EPI_ISL_7560191, EPI_ISL_7560192, EPI_ISL_7560194, EPI_ISL_7560211, EPI_ISL_7560228, EPI_ISL_7560245, EPI_ISL_7560247, EPI_ISL_7560254, EPI_ISL_7560269, EPI_ISL_7560284, EPI_ISL_7560291, EPI_ISL_7560293, EPI_ISL_7560295, EPI_ISL_7560300, EPI_ISL_7560324, EPI_ISL_7560333, EPI_ISL_7560337, EPI_ISL_7560345, EPI_ISL_7560354, EPI_ISL_7560355, EPI_ISL_7560384, EPI_ISL_7560389, EPI_ISL_7560390, EPI_ISL_7560393, EPI_ISL_7560466, EPI_ISL_7560520, EPI_ISL_7560584, EPI_ISL_7560632, EPI_ISL_7560648, EPI_ISL_7560715, EPI_ISL_7560752, EPI_ISL_7560781, EPI_ISL_7560803, EPI_ISL_7560823, EPI_ISL_7560833, EPI_ISL_7560854, EPI_ISL_7560904, EPI_ISL_7560952, EPI_ISL_7561012, EPI_ISL_7561019, EPI_ISL_7561026, EPI_ISL_7561097, EPI_ISL_7561162, EPI_ISL_7561163, EPI_ISL_7561172, EPI_ISL_7571908, EPI_ISL_7571910, EPI_ISL_7571950, EPI_ISL_7571953, EPI_ISL_7571956, EPI_ISL_7571957, EPI_ISL_7571962, EPI_ISL_7571998, EPI_ISL_7572132, EPI_ISL_7572153, EPI_ISL_7572163, EPI_ISL_7572209, EPI_ISL_7572219, EPI_ISL_7572256, EPI_ISL_7572292, EPI_ISL_7572329, EPI_ISL_7572355, EPI_ISL_7572415, EPI_ISL_7572449, EPI_ISL_7572473, EPI_ISL_7572483, EPI_ISL_7572501, EPI_ISL_7572515, EPI_ISL_7572541, EPI_ISL_7572576, EPI_ISL_7572591, EPI_ISL_7572602, EPI_ISL_7572611, EPI_ISL_7572628, EPI_ISL_7572647, EPI_ISL_7572689, EPI_ISL_7572720, EPI_ISL_7572723, EPI_ISL_7572728, EPI_ISL_7572765, EPI_ISL_7572792, EPI_ISL_7572798, EPI_ISL_7572819, EPI_ISL_7572820, EPI_ISL_7572821, EPI_ISL_7572823, EPI_ISL_7572825, EPI_ISL_7572837, EPI_ISL_7572875, EPI_ISL_7574310, EPI_ISL_7574318, EPI_ISL_7574345, EPI_ISL_7574378, EPI_ISL_7574414, EPI_ISL_7574420, EPI_ISL_7574428, EPI_ISL_7574444, EPI_ISL_7574460, EPI_ISL_7574469, EPI_ISL_7574473, EPI_ISL_7574479, EPI_ISL_7574506, EPI_ISL_7574536, EPI_ISL_7574540, EPI_ISL_7574545, EPI_ISL_7574550, EPI_ISL_7574553, EPI_ISL_7574557, EPI_ISL_7574580, EPI_ISL_7574583, EPI_ISL_7574596, EPI_ISL_7574608, EPI_ISL_7574609, EPI_ISL_7574624, EPI_ISL_7574638, EPI_ISL_7574680, EPI_ISL_7574685, EPI_ISL_7574697, EPI_ISL_7574701, EPI_ISL_7574713, EPI_ISL_7574714, EPI_ISL_7574715, EPI_ISL_7574726, EPI_ISL_7574737, EPI_ISL_7574753, EPI_ISL_7574761, EPI_ISL_7574770, EPI_ISL_7574779, EPI_ISL_7574787, EPI_ISL_7574850, EPI_ISL_7574857, EPI_ISL_7574872, EPI_ISL_7574875, EPI_ISL_7574929, EPI_ISL_7574938, EPI_ISL_7574953, EPI_ISL_7574957, EPI_ISL_7574965, EPI_ISL_7574967, EPI_ISL_7574975, EPI_ISL_7575010, EPI_ISL_7575029, EPI_ISL_7575093, EPI_ISL_7575243, EPI_ISL_7575433, EPI_ISL_7575537, EPI_ISL_7575747, EPI_ISL_7575779, EPI_ISL_7576056, EPI_ISL_7576084, EPI_ISL_7576236, EPI_ISL_7576420, EPI_ISL_7576528, EPI_ISL_7576627, EPI_ISL_7576647, EPI_ISL_7576653, EPI_ISL_7576689, EPI_ISL_7576697, EPI_ISL_7576759, EPI_ISL_7576788, EPI_ISL_7576795, EPI_ISL_7576855, EPI_ISL_7576879, EPI_ISL_7576933, EPI_ISL_7576976, EPI_ISL_7577022, EPI_ISL_7577089, EPI_ISL_7577095, EPI_ISL_7577133, EPI_ISL_7577141, EPI_ISL_7577182, EPI_ISL_7577201, EPI_ISL_7577217, EPI_ISL_7577244, EPI_ISL_7577302, EPI_ISL_7577306, EPI_ISL_7577352, EPI_ISL_7577387, EPI_ISL_7577409, EPI_ISL_7577420, EPI_ISL_7577462, EPI_ISL_7577527, EPI_ISL_7577528, EPI_ISL_7577743, EPI_ISL_7577877, EPI_ISL_7577953, EPI_ISL_7577956, EPI_ISL_7577964, EPI_ISL_7577972, EPI_ISL_7577974, EPI_ISL_7577994, EPI_ISL_7578143, EPI_ISL_7578256, EPI_ISL_7578292, EPI_ISL_7578340, EPI_ISL_7578353, EPI_ISL_7578459, EPI_ISL_7578518, EPI_ISL_7578663, EPI_ISL_7578729, EPI_ISL_7579103, EPI_ISL_7579594, EPI_ISL_7579656, EPI_ISL_7579923, EPI_ISL_7579947, EPI_ISL_7579965, EPI_ISL_7579967, EPI_ISL_7579991, EPI_ISL_7579992, EPI_ISL_7580021, EPI_ISL_7580025, EPI_ISL_7580029, EPI_ISL_7580037, EPI_ISL_7580044, EPI_ISL_7580054, EPI_ISL_7580104, EPI_ISL_7580109, EPI_ISL_7580116, EPI_ISL_7580135, EPI_ISL_7580145, EPI_ISL_7580170, EPI_ISL_7580180, EPI_ISL_7580205, EPI_ISL_7580335, EPI_ISL_7580388, EPI_ISL_7580420, EPI_ISL_7580447, EPI_ISL_7580448, EPI_ISL_7580476, EPI_ISL_7580535, EPI_ISL_7580548, EPI_ISL_7580555, EPI_ISL_7580568, EPI_ISL_7580599, EPI_ISL_7580623, EPI_ISL_7580637, EPI_ISL_7580682, EPI_ISL_7580705, EPI_ISL_7580729, EPI_ISL_7580732, EPI_ISL_7580843, EPI_ISL_7580850, EPI_ISL_7580851, EPI_ISL_7580869, EPI_ISL_7580876, EPI_ISL_7580886, EPI_ISL_7580965, EPI_ISL_7580997, EPI_ISL_7581008, EPI_ISL_7581047, EPI_ISL_7581088, EPI_ISL_7581089, EPI_ISL_7581099, EPI_ISL_7581114, EPI_ISL_7581163, EPI_ISL_7581192, EPI_ISL_7581321, EPI_ISL_7581351, EPI_ISL_7581399, EPI_ISL_7581409, EPI_ISL_7581445, EPI_ISL_7581525, EPI_ISL_7581571, EPI_ISL_7581625, EPI_ISL_7581641, EPI_ISL_7581662, EPI_ISL_7581663, EPI_ISL_7581676, EPI_ISL_7581768, EPI_ISL_7581809, EPI_ISL_7582242, EPI_ISL_7582510, EPI_ISL_7582511, EPI_ISL_7582572, EPI_ISL_7582651, EPI_ISL_7582661, EPI_ISL_7582674, EPI_ISL_7582788, EPI_ISL_7582935, EPI_ISL_7583017, EPI_ISL_7583130, EPI_ISL_7583138, EPI_ISL_7583151, EPI_ISL_7583185, EPI_ISL_7583206, EPI_ISL_7583284, EPI_ISL_7583310, EPI_ISL_7583376, EPI_ISL_7583481, EPI_ISL_7583574, EPI_ISL_7583607, EPI_ISL_7583696, EPI_ISL_7583748, EPI_ISL_7583830, EPI_ISL_7583878, EPI_ISL_7583942, EPI_ISL_7584036, EPI_ISL_7584057, EPI_ISL_7584077, EPI_ISL_7584766, EPI_ISL_7584869, EPI_ISL_7584965, EPI_ISL_7585008, EPI_ISL_7585036, EPI_ISL_7585211, EPI_ISL_7585238, EPI_ISL_7586597, EPI_ISL_7586606, EPI_ISL_7586607, EPI_ISL_7586608, EPI_ISL_7586789, EPI_ISL_7586796, EPI_ISL_7586807, EPI_ISL_7586819, EPI_ISL_7586841, EPI_ISL_7586844, EPI_ISL_7586854, EPI_ISL_7586865, EPI_ISL_7586900, EPI_ISL_7586911, EPI_ISL_7586933, EPI_ISL_7586943, EPI_ISL_7587053, EPI_ISL_7587071, EPI_ISL_7587072, EPI_ISL_7587082, EPI_ISL_7587159, EPI_ISL_7587216, EPI_ISL_7587225, EPI_ISL_7587236, EPI_ISL_7587349, EPI_ISL_7587389, EPI_ISL_7587438, EPI_ISL_7587459, EPI_ISL_7587484, EPI_ISL_7587601, EPI_ISL_7587618, EPI_ISL_7587700, EPI_ISL_7587790, EPI_ISL_7587791, EPI_ISL_7587834, EPI_ISL_7587872, EPI_ISL_7587936, EPI_ISL_7587950, EPI_ISL_7588011, EPI_ISL_7624460, EPI_ISL_7624566, EPI_ISL_7624618, EPI_ISL_7624627, EPI_ISL_7624684, EPI_ISL_7624691, EPI_ISL_7624699, EPI_ISL_7624754, EPI_ISL_7624783, EPI_ISL_7624802, EPI_ISL_7624813, EPI_ISL_7624829, EPI_ISL_7624840, EPI_ISL_7624918, EPI_ISL_7624952, EPI_ISL_7625001, EPI_ISL_7625005, EPI_ISL_7625025, EPI_ISL_7625079, EPI_ISL_7625096, EPI_ISL_7625150, EPI_ISL_7625171, EPI_ISL_7625192, EPI_ISL_7625311, EPI_ISL_7625376, EPI_ISL_7625386, EPI_ISL_7625400, EPI_ISL_7625414, EPI_ISL_7625433, EPI_ISL_7625439, EPI_ISL_7625463, EPI_ISL_7625472, EPI_ISL_7625473, EPI_ISL_7625479, EPI_ISL_7625494, EPI_ISL_7625541, EPI_ISL_7625547, EPI_ISL_7625552, EPI_ISL_7625586, EPI_ISL_7625588, EPI_ISL_7625589, EPI_ISL_7625593, EPI_ISL_7625620, EPI_ISL_7625623, EPI_ISL_7625629, EPI_ISL_7625639, EPI_ISL_7625654, EPI_ISL_7625697, EPI_ISL_7625701, EPI_ISL_7625707, EPI_ISL_7625722, EPI_ISL_7625754, EPI_ISL_7625771, EPI_ISL_7626567, EPI_ISL_7626606, EPI_ISL_7626607, EPI_ISL_7626612, EPI_ISL_7626615, EPI_ISL_7626620, EPI_ISL_7626625, EPI_ISL_7626630, EPI_ISL_7626633, EPI_ISL_7626644, EPI_ISL_7626649, EPI_ISL_7626662, EPI_ISL_7626664, EPI_ISL_7626672, EPI_ISL_7626680, EPI_ISL_7626683, EPI_ISL_7626692, EPI_ISL_7626696, EPI_ISL_7626698, EPI_ISL_7626701, EPI_ISL_7626704, EPI_ISL_7626711, EPI_ISL_7626714, EPI_ISL_7626735, EPI_ISL_7626743, EPI_ISL_7626748, EPI_ISL_7626755, EPI_ISL_7626765, EPI_ISL_7626839, EPI_ISL_7626852, EPI_ISL_7626862, EPI_ISL_7626863, EPI_ISL_7626870, EPI_ISL_7626874, EPI_ISL_7626894, EPI_ISL_7626906, EPI_ISL_7626922, EPI_ISL_7626928, EPI_ISL_7626937, EPI_ISL_7626950, EPI_ISL_7626954, EPI_ISL_7626967, EPI_ISL_7626969, EPI_ISL_7626973, EPI_ISL_7626975, EPI_ISL_7626976, EPI_ISL_7626977, EPI_ISL_7626995, EPI_ISL_7626997, EPI_ISL_7627006, EPI_ISL_7627008, EPI_ISL_7627016, EPI_ISL_7627027, EPI_ISL_7627039, EPI_ISL_7627053, EPI_ISL_7627054, EPI_ISL_7627058, EPI_ISL_7627063, EPI_ISL_7627091, EPI_ISL_7627097, EPI_ISL_7627129, EPI_ISL_7627136, EPI_ISL_7627139, EPI_ISL_7627154, EPI_ISL_7627160, EPI_ISL_7627165, EPI_ISL_7627168, EPI_ISL_7627176, EPI_ISL_7627179, EPI_ISL_7627196, EPI_ISL_7627197, EPI_ISL_7627200, EPI_ISL_7627216, EPI_ISL_7627217, EPI_ISL_7627236, EPI_ISL_7629194, EPI_ISL_7629214, EPI_ISL_7629269, EPI_ISL_7629303, EPI_ISL_7629403, EPI_ISL_7629484, EPI_ISL_7629510, EPI_ISL_7629604, EPI_ISL_7629619, EPI_ISL_7629646, EPI_ISL_7629705, EPI_ISL_7629753, EPI_ISL_7629785, EPI_ISL_7629792, EPI_ISL_7629817, EPI_ISL_7629973, EPI_ISL_7629979, EPI_ISL_7630007, EPI_ISL_7630026, EPI_ISL_7630050, EPI_ISL_7630068, EPI_ISL_7630098, EPI_ISL_7630868, EPI_ISL_7630897, EPI_ISL_7630900, EPI_ISL_7630918, EPI_ISL_7630925, EPI_ISL_7630928, EPI_ISL_7631018, EPI_ISL_7631029, EPI_ISL_7631048, EPI_ISL_7631073, EPI_ISL_7631097, EPI_ISL_7631131, EPI_ISL_7631148, EPI_ISL_7631149, EPI_ISL_7631206, EPI_ISL_7631223, EPI_ISL_7631236, EPI_ISL_7631257, EPI_ISL_7631498, EPI_ISL_7631754, EPI_ISL_7632315, EPI_ISL_7632790, EPI_ISL_7632843, EPI_ISL_7632860, EPI_ISL_7632864, EPI_ISL_7632879, EPI_ISL_7632885, EPI_ISL_7632914, EPI_ISL_7632933, EPI_ISL_7632935, EPI_ISL_7632958, EPI_ISL_7632959, EPI_ISL_7632966, EPI_ISL_7632974, EPI_ISL_7632997, EPI_ISL_7633011, EPI_ISL_7633032, EPI_ISL_7633043, EPI_ISL_7633046, EPI_ISL_7633084, EPI_ISL_7633100, EPI_ISL_7633102, EPI_ISL_7633139, EPI_ISL_7633149, EPI_ISL_7633268, EPI_ISL_7633306, EPI_ISL_7633315, EPI_ISL_7633396, EPI_ISL_7633400, EPI_ISL_7633434, EPI_ISL_7633451, EPI_ISL_7633514, EPI_ISL_7633572, EPI_ISL_7633596, EPI_ISL_7633662, EPI_ISL_7633698, EPI_ISL_7633744, EPI_ISL_7633755, EPI_ISL_7633765, EPI_ISL_7633766, EPI_ISL_7633767, EPI_ISL_7633769, EPI_ISL_7633770, EPI_ISL_7633776, EPI_ISL_7633787, EPI_ISL_7633791, EPI_ISL_7633800, EPI_ISL_7633822, EPI_ISL_7633836, EPI_ISL_7633837, EPI_ISL_7633847, EPI_ISL_7633856, EPI_ISL_7633860, EPI_ISL_7633873, EPI_ISL_7633875, EPI_ISL_7633886, EPI_ISL_7633916, EPI_ISL_7633917, EPI_ISL_7633936, EPI_ISL_7633942, EPI_ISL_7633944, EPI_ISL_7633976, EPI_ISL_7633978, EPI_ISL_7633993, EPI_ISL_7634000, EPI_ISL_7634001, EPI_ISL_7634009, EPI_ISL_7634025, EPI_ISL_7634053, EPI_ISL_7634060, EPI_ISL_7634074, EPI_ISL_7634102, EPI_ISL_7634116, EPI_ISL_7634119, EPI_ISL_7634122, EPI_ISL_7634133, EPI_ISL_7634137, EPI_ISL_7634138, EPI_ISL_7634145, EPI_ISL_7634151, EPI_ISL_7634164, EPI_ISL_7634187, EPI_ISL_7634201, EPI_ISL_7634209, EPI_ISL_7634211, EPI_ISL_7634222, EPI_ISL_7634224, EPI_ISL_7634235, EPI_ISL_7634249, EPI_ISL_7634261, EPI_ISL_7634262, EPI_ISL_7634269, EPI_ISL_7634270, EPI_ISL_7634294, EPI_ISL_7634298, EPI_ISL_7634337, EPI_ISL_7634360, EPI_ISL_7634364, EPI_ISL_7634383, EPI_ISL_7634393, EPI_ISL_7634395, EPI_ISL_7634414, EPI_ISL_7634415, EPI_ISL_7634418, EPI_ISL_7634419, EPI_ISL_7634442, EPI_ISL_7634446, EPI_ISL_7634447, EPI_ISL_7634453, EPI_ISL_7634463, EPI_ISL_7634466, EPI_ISL_7634480, EPI_ISL_7634482, EPI_ISL_7634510, EPI_ISL_7634531, EPI_ISL_7634540, EPI_ISL_7634550, EPI_ISL_7634552, EPI_ISL_7634564, EPI_ISL_7634566, EPI_ISL_7634583, EPI_ISL_7634587, EPI_ISL_7634595, EPI_ISL_7634607, EPI_ISL_7634610, EPI_ISL_7634623, EPI_ISL_7634647, EPI_ISL_7634651, EPI_ISL_7634656, EPI_ISL_7634659, EPI_ISL_7634678, EPI_ISL_7634680, EPI_ISL_7634684, EPI_ISL_7634703, EPI_ISL_7634704, EPI_ISL_7634712, EPI_ISL_7634739, EPI_ISL_7634756, EPI_ISL_7634769, EPI_ISL_7634770, EPI_ISL_7634785, EPI_ISL_7634794, EPI_ISL_7634813, EPI_ISL_7634821, EPI_ISL_7634824, EPI_ISL_7634832, EPI_ISL_7634833, EPI_ISL_7634846, EPI_ISL_7634855, EPI_ISL_7634865, EPI_ISL_7634867, EPI_ISL_7634871, EPI_ISL_7634878, EPI_ISL_7635532, EPI_ISL_7635602, EPI_ISL_7635633, EPI_ISL_7635708, EPI_ISL_7635758, EPI_ISL_7635793, EPI_ISL_7635832, EPI_ISL_7653895, EPI_ISL_7653923, EPI_ISL_7654605, EPI_ISL_7654642, EPI_ISL_7654648, EPI_ISL_7654670, EPI_ISL_7654771, EPI_ISL_7654781, EPI_ISL_7654792, EPI_ISL_7654807, EPI_ISL_7654886, EPI_ISL_7654898, EPI_ISL_7654955, EPI_ISL_7654967, EPI_ISL_7654971, EPI_ISL_7654983, EPI_ISL_7654986, EPI_ISL_7654996, EPI_ISL_7655030, EPI_ISL_7655042, EPI_ISL_7655057, EPI_ISL_7655112, EPI_ISL_7655138, EPI_ISL_7655162, EPI_ISL_7655186, EPI_ISL_7655188, EPI_ISL_7655199, EPI_ISL_7655209, EPI_ISL_7655216, EPI_ISL_7655232, EPI_ISL_7655235, EPI_ISL_7655240, EPI_ISL_7655246, EPI_ISL_7655312, EPI_ISL_7655313, EPI_ISL_7655316, EPI_ISL_7655318, EPI_ISL_7655343, EPI_ISL_7655353, EPI_ISL_7655382, EPI_ISL_7655384, EPI_ISL_7655401, EPI_ISL_7655409, EPI_ISL_7655427, EPI_ISL_7655458, EPI_ISL_7655472, EPI_ISL_7655480, EPI_ISL_7655483, EPI_ISL_7655510, EPI_ISL_7655515, EPI_ISL_7655520, EPI_ISL_7655550, EPI_ISL_7655561, EPI_ISL_7655567, EPI_ISL_7655572, EPI_ISL_7655575, EPI_ISL_7655576, EPI_ISL_7655578, EPI_ISL_7655583, EPI_ISL_7655585, EPI_ISL_7655596, EPI_ISL_7655597, EPI_ISL_7655603, EPI_ISL_7655621, EPI_ISL_7655628, EPI_ISL_7655633, EPI_ISL_7655646, EPI_ISL_7655660, EPI_ISL_7655661, EPI_ISL_7655708, EPI_ISL_7655709, EPI_ISL_7655720, EPI_ISL_7655728, EPI_ISL_7655742, EPI_ISL_7655745, EPI_ISL_7655749, EPI_ISL_7655774, EPI_ISL_7655778, EPI_ISL_7655779, EPI_ISL_7655780, EPI_ISL_7655801, EPI_ISL_7655823, EPI_ISL_7655824, EPI_ISL_7655829, EPI_ISL_7655830, EPI_ISL_7655838, EPI_ISL_7655845, EPI_ISL_7655848, EPI_ISL_7655856, EPI_ISL_7655857, EPI_ISL_7655859, EPI_ISL_7655861, EPI_ISL_7655882, EPI_ISL_7655898, EPI_ISL_7655906, EPI_ISL_7655910, EPI_ISL_7655914, EPI_ISL_7655921, EPI_ISL_7655922, EPI_ISL_7655924, EPI_ISL_7655931, EPI_ISL_7656361, EPI_ISL_7656366, EPI_ISL_7656368, EPI_ISL_7656400, EPI_ISL_7656409, EPI_ISL_7656440, EPI_ISL_7656447, EPI_ISL_7656453, EPI_ISL_7656488, EPI_ISL_7656493, EPI_ISL_7656508, EPI_ISL_7656529, EPI_ISL_7656537, EPI_ISL_7656547, EPI_ISL_7656550, EPI_ISL_7656552, EPI_ISL_7656557, EPI_ISL_7656558, EPI_ISL_7656561, EPI_ISL_7656567, EPI_ISL_7656587, EPI_ISL_7656589, EPI_ISL_7656594, EPI_ISL_7656596, EPI_ISL_7656601, EPI_ISL_7656628, EPI_ISL_7656629, EPI_ISL_7656632, EPI_ISL_7656675, EPI_ISL_7656681, EPI_ISL_7656688, EPI_ISL_7656717, EPI_ISL_7656724, EPI_ISL_7656730, EPI_ISL_7656731, EPI_ISL_7656733, EPI_ISL_7656742, EPI_ISL_7656747, EPI_ISL_7656760, EPI_ISL_7656770, EPI_ISL_7656781, EPI_ISL_7656815, EPI_ISL_7656839, EPI_ISL_7656854, EPI_ISL_7656865, EPI_ISL_7656871, EPI_ISL_7656876, EPI_ISL_7656890, EPI_ISL_7656905, EPI_ISL_7656923, EPI_ISL_7656942, EPI_ISL_7656979, EPI_ISL_7657009, EPI_ISL_7657010, EPI_ISL_7657021, EPI_ISL_7657025, EPI_ISL_7657028, EPI_ISL_7657038, EPI_ISL_7657045, EPI_ISL_7657046, EPI_ISL_7657061, EPI_ISL_7657064, EPI_ISL_7657065, EPI_ISL_7657066, EPI_ISL_7657067, EPI_ISL_7657068, EPI_ISL_7657069, EPI_ISL_7657077, EPI_ISL_7657082, EPI_ISL_7657087			
see above	Lighthouse Lab in Milton Keynes	Wellcome Sanger Institute for the COVID-19 Genomics UK (COG-UK) Consortium	Cordelia Langford; David K. Jackson; Dominic Kwiatkowski; Ewan Harrison; Ian Johnston; Jeffrey Barrett; John Sillitoe on behalf of the Wellcome Sanger Institute COVID-19 Surveillance Team; Roberto Amato; Sonia Goncalves; The Lighthouse Lab in Milton Keynes and Alex Alderton
EPI_ISL_7201997, EPI_ISL_7302326, EPI_ISL_7302955, EPI_ISL_7303330, EPI_ISL_7625911, EPI_ISL_7625953, EPI_ISL_7626237, EPI_ISL_7626242, EPI_ISL_7626494, EPI_ISL_7628520, EPI_ISL_7628997, EPI_ISL_7629036, EPI_ISL_7630124, EPI_ISL_7630316, EPI_ISL_7630810			
see above	Lighthouse Laboratory Plymouth	Wellcome Sanger Institute for the COVID-19 Genomics UK (COG-UK) Consortium	Cordelia Langford; David K. Jackson; Dominic Kwiatkowski; Ewan Harrison; Ian Johnston; Jeffrey Barrett; John Sillitoe on behalf of the Wellcome Sanger Institute COVID-19 Surveillance Team; Lighthouse Laboratory Plymouth and Alex Alderton; Roberto Amato; Sonia Goncalves
EPI_ISL_7662859, EPI_ISL_7662885	Louisiana Office of Public Heath Laboratory	LSUHS Emerging Viral Threat Laboratory	Adrian Almodovar; Alexander Mijalis; Andrew D. Yurochko; Christopher G. Kevil; Gregory L. Ware; Jennifer L. Carroll; Jeremy P. Kamil; John A. Vanchiere; Krista Queen; Maarten Van Diest; Rona S. Scott
EPI_ISL_7413422, EPI_ISL_7413427, EPI_ISL_7413439, EPI_ISL_7413450, EPI_ISL_7597006	MB-Cadham Provincial laboratory	National Microbiology Laboratory (NML)	Adam Hedley; Anna Majer; Anneliese Landgraff; Ayo Bolaji; CanCOGeN's metadata curation team; Darian Hole; David Alexander; Elsie Grudeski; Gary Van Domselaar; Grace Seo; Janna Holowick; Jared Bullard; Jennifer Tanner; Joanne Sanders; Kerry Dust; Kirsten Biggar; Lori Johnson; Madison Chapel; Morag Graham; Natalie Knox; Nathalie Bastien; Paul Van Caeseele; Philip Mabon; Public Health Agency of Canada CanCOGeN team; Rhiannon Huzarewich; Russell Mandes; Shari Tyson; Timothy Booth; Yan Li
EPI_ISL_7405692	MCL Medizinische Laboratorien, Hauptstandort Niederwangen	Institute for Infectious Diseases, University of Bern	Alban Ramette; Christian Baumann; Cora Sägesser; Franziska Suter-Riniker; Loïc Borcard; Miguel A Terrazos Miani; Nicole Liechti; Pascal Bittel; Peter Keller; Sonja Gempeler; Stefan Neuenschwander; Stephen L Leib
EPI_ISL_7608613, EPI_ISL_7609854	MIRIALIS CLUSES BECHET	CNR Virus des Infections Respiratoires - France SUD	Antonin Bal; Bruno Lina; Bruno Simon; Gregory Destras; Gwendolyne Burfin; Hadrien Regue; Laurence Josset; Martine Valette; Quentin Semanas
EPI_ISL_7615139, EPI_ISL_7661024	MS PHL	MS PHL	Beata Karowlewicz; Daphne Ware
EPI_ISL_7444012, EPI_ISL_7444038, EPI_ISL_7444057	MVZ Labor Dr. Fenner und Kollegen (Standort Hamburg)	Robert Koch Institute	
EPI_ISL_7418337	MVZ Labor Dr. Limbach & Kollegen GbR	Robert Koch Institute	
EPI_ISL_7542261	MVZ Rheumatologie und Autoimmunmedizin Hamburg GmbH	Heinrich Pette Institute, Leibniz Institute for Experimental Virology	Adam Grundhoff; Alexis Robitaille; Johannes Knobloch; Martin Aepfelbacher; Nicole Fischer; Thomas Günther
EPI_ISL_7620047	Makeni Molecular Laboratory	Central Public Health Reference Laboratory	Allan K.O. Campbell; Anu J. Williams; Choe Miller; Doris Harding; Dr. Raoul E.G. Wadoum; Prof. Foday Sahr; Prof. Isatta Wurie; Prof. Sahr Gevao; Samuel Sorie; Warren C. Yiaba; Zein Souma

EPI_ISL_7154400, EPI_ISL_7154401, EPI_ISL_7154402, EPI_ISL_7500444	Mako Medical	Centers for Disease Control and Prevention Division of Viral Diseases, Pathogen Discovery	Benjamin Rambo-Martin; Christopher Gulvick; Clinton Paden; Dakota Howard; Dhwani Batra; Duncan MacCannell; Erisa Sula; Jason Caravas; Kristine Lacek; Lauren Moon; Matthew Schmerer; Matthew Tugwell; Peter Cook; Scott Sammons; Shatavia Morrison; Tymeckia Kendall; Victoria Caban Figueroa; Yvette Unoarumhi
EPI_ISL_7464406, EPI_ISL_7464407, EPI_ISL_7464408	Malawi Liverpool Wellcome Trust Clinical Research Program	Malawi Liverpool Wellcome Trust Clinical Research Program	Belson Kutambe; Ben Morton; Catherine Anscombe; Kondwani Jambo; Mavis Menyere; Philip Ashton; Sam Lissauer
EPI_ISL_7404794	Maryland Genomics, Institute for Genome Sciences, University of Maryland School of Medicine	Maryland Genomics, Institute for Genome Sciences, University of Maryland School of Medicine	Claire M; Fraser; George; Hazen; Holly; Humphrys; Jacques; Jain; Kevin; Kranthi; Lisa D; Luke J; Mike; Ott; Ravel; Regan; Roussey; Sadzewicz; Sandra; Tallon; Tracy; Vavikolanu
EPI_ISL_7469697	Mass General Brigham	Mass General Brigham	A.E.; Adams, G.; Anahtar, M.; B.L.; B.W.; Bauer, M.; Birren; Branda, J.; Carter, A.; Cerrato, F.; Chaluvadi, S.; Chapman; Cusick, C.; D.J.; DeRuff, K.; E. and Sabeti; Flowers, K.; Gallagher, G.; Gladden-Young, A.; Gnirke, A.; Harris, J.; J.E.; K.J.; LaRocque, R.; Lagerborg, K.; Lemieux; Lin; Loreth, C.; MacInnis; Neumann, A.; Normandin, E.; P.C.; Park; Pierce, V.; Reilly, S.; Rosenberg; Rudy, M.; Ryan, E.; S.B.; Shaw, B.; Siddle; Slater, D.; Smole, S.; Tomkins-Tinch, C.; Turbett, S.; Uddin, R.
EPI_ISL_6886593, EPI_ISL_6886594, EPI_ISL_6886595, EPI_ISL_6886596, EPI_ISL_7590775, EPI_ISL_7590919, EPI_ISL_7590929, EPI_ISL_7590955, EPI_ISL_7590991, EPI_ISL_7591005, EPI_ISL_7591006, EPI_ISL_7591007, EPI_ISL_7591008, EPI_ISL_7591009, EPI_ISL_7591010, EPI_ISL_7591011, EPI_ISL_7591012, EPI_ISL_7591013, EPI_ISL_7591014, EPI_ISL_7591015, EPI_ISL_7591016, EPI_ISL_7591018, EPI_ISL_7591019			
see above	Max von Pettenkofer Institute, Virology, National Reference Center for Retroviruses, LMU Munich	Laboratory for Functional Genome Analysis; Dept. Genomics; Gene Center of the LMU Munich	Alexander Graf; Helmut Blum; Max Muenchhoff; Oliver Keppler; Stefan Krebs
EPI_ISL_7267259, EPI_ISL_7470216, EPI_ISL_7470264, EPI_ISL_7470331	Medical Microbiology Unit, Department for Laboratory Medicine, Drammen Hospital, Vestre Viken Health Trust	Norwegian Institute of Public Health, Department of Virology	Atiya R Ali; Debech Nadia; Engebretsen Serina Beate; Garcia Llorente Ignacio; Hilde Elshaug; Hilde Vollan; Jon Bråte; Kamilla Heddeland Instefjord; Karoline Bragstad; Kathrine Stene-Johansen; Line Victoria Moen; Marie Paulsen Madsen; Olav Hungnes; Pedersen Benedikte Nevjen; Rasmus Riis Kopperud
EPI_ISL_7443804, EPI_ISL_7443805, EPI_ISL_7443809, EPI_ISL_7443815	Medizinische Laboratorien Düsseldorf	Robert Koch Institute	
EPI_ISL_7416680	Medizinisches Versorgungszentrum für Labormedizin und Mikrobiologie Ruhr GmbH - mvzlm RUHR GmbH	Robert Koch Institute	
EPI_ISL_6854346, EPI_ISL_6854347, EPI_ISL_6854348	Microbiologia e Virologia Cotugno	Microbiologia e Virologia Cotugno	Antonio Canonico; Antonio Fascione; Claudia Tiberio; Enza Mallardo; Francesco Nappo; Giovanni D'Auria; Giuseppe di Gennaro; Ilaria Cavallaro; Luigi Atripaldi
EPI_ISL_7462324	Microbiology Department, University Hospital Araba	Microbiology Department, University Hospital Donostia	Cilla G.; Gomez M; Hernaez S; Marimon JM; Martin-Peñaranda T; Montes M; Piñeiro L; Sorarrain A
EPI_ISL_7502103, EPI_ISL_7502107	Microbiology Department. Complexo Hospitalario Universitario de Vigo	Microbiology Department. Complexo Hospitalario Universitario de Vigo	Alvarez M; Cabrera JJ; Carballo R; Cortizo S; Davina C; Martinez L; Mediero G; Pena I; Perez S; Potel C; Regueiro B; Rey S; Vasallo FJ; del-Campo V
EPI_ISL_7566142	Ministry of Health Turkey	Ministry of Health Turkey	Fatma Bayrakdar; Gulay Korukluoglu; Suleyman Yalcin; Yasemin Cosgun
EPI_ISL_7496678	Minnesota Department of Health, Public Health Laboratory	Minnesota Department of Health, Public Health Laboratory	Alyssa Mondelli; Elizabeth Horn; Jacob Garfin; Kelly Pung; Matt Plumb; Sarah Namugenyi; and Xiong Wang
EPI_ISL_6963002	Mirialis	CNR Virus des Infections Respiratoires - France SUD	Antoine Oblette; Antonin Bal; Bruno Lina; Bruno Simon; Camille Delcloitre; Eva Oddoux; Florence Morfin; Gregory Destras; Gwendolyne Burfin; Hadrien Regue; Hervé Crehalet; Jean François Bore; Jeremy Cordier; Laurence Josset; Martine Valette; Noémie Fessy; Quentin Semanas; Richard Chalvignac; Thibault Corsin; Thibault Gouiran
EPI_ISL_7661074	Mirimus	Biotia	Christopher Mason; David Danko; Dorottya Nagy-Szakal; Mara Couto-Rodriguez; Marilyne Debieu; Niamh O'Hara; Xavier Jirau Serrano
EPI_ISL_7193991, EPI_ISL_7286479, EPI_ISL_7516233, EPI_ISL_7519931, EPI_ISL_7527590, EPI_ISL_7527959	Molekylær Medicinsk Afdeling, Aarhus University Hospital, Aarhus, Denmark	Statens Serum Institut Bioinformatics and Microbial Genomics	Danish Covid-19 Genome Consortium
EPI_ISL_7501186	Mount Auburn Hospital via Lahey Hospital	New England Biolabs	Abel, G.; B.W.; C.J.; Colgrove, R.; Duncan, R.; Elfahal, M.; Flynn; Heim, K.; Karolides, M.; L. and Langhorst; Michaels, L.; Pinet, K.; Skelton, T.; Sun
EPI_ISL_7545652, EPI_ISL_7545653, EPI_ISL_7545654, EPI_ISL_7545658, EPI_ISL_7545660, EPI_ISL_7545661, EPI_ISL_7545662, EPI_ISL_7545663, EPI_ISL_7545664, EPI_ISL_7545665, EPI_ISL_7545666, EPI_ISL_7545667, EPI_ISL_7545668, EPI_ISL_7545669, EPI_ISL_7545670, EPI_ISL_7545671, EPI_ISL_7545673, EPI_ISL_7545674, EPI_ISL_7545675			
see above	NHLS Livingstone Laboratory	CERI, Centre for Epidemic Response and Innvoation, Stellenbosch University and KRISP, KZN Research Innovation and Sequencing Platform, UKZN.	Arisha Maharaj; Giandhari J; Naidoo Y; Oluwakemi Laguda-Akingba and Nokukhanya Mdlalose; Pillay S; Ramphal U; Ramphal Y; San JE; Tegally H; Tshiabuila D; Wilkinson E; de Oliveira T
EPI_ISL_7381208, EPI_ISL_7381209, EPI_ISL_7381210, EPI_ISL_7381211, EPI_ISL_7381212, EPI_ISL_7381213, EPI_ISL_7381214, EPI_ISL_7381215, EPI_ISL_7381216, EPI_ISL_7381217, EPI_ISL_7381218, EPI_ISL_7381219, EPI_ISL_7381220, EPI_ISL_7381221, EPI_ISL_7381222			
see above	NHLS Port Elizabeth Laboratory	CERI, Centre for Epidemic Response and Innvoation, Stellenbosch University and KRISP, KZN Research Innovation and Sequencing Platform, UKZN.	Arisha Maharaj; Giandhari J; Moir M; Naidoo Y; Oluwakemi Laguda-Akingba and Nokukhanya Mdlalose; Pillay S; Ramphal U; Ramphal Y; San JE; Tegally H; Tshiabuila D; Wilkinson E; de Oliveira T; van Wyk S
EPI_ISL_7462390, EPI_ISL_7462391, EPI_ISL_7462392, EPI_ISL_7462393, EPI_ISL_7462397, EPI_ISL_7462398, EPI_ISL_7462399, EPI_ISL_7462400, EPI_ISL_7462401, EPI_ISL_7462402, EPI_ISL_7462403, EPI_ISL_7462404			
see above	NHLS Universitas Academic	Division of Medical Virology, National Health Laboratory Service (NHLS), Tygerberg Hospital / Stellenbosch University	D Goedhals; Emmanuel Ogunbayo; MM Nyaga; MT Mogotsi; P Nthiga; PA Bester; Shannon Wilson; Susan Engelbrecht; T de Oliveira; Tongai Maponga; Wolfgang Preiser
EPI_ISL_7605460, EPI_ISL_7605516	NJDOH, Public Health and Environmental Laboratories	NJ_PHEL	Allison Roder; Byeong Jeong; Chelsea San Filippo; Dana Woell; Jacquelyn Deverell; Lindsey Bodnar; Maria-Magdalene Pugliese; Mohammad M. Ali; Ryan Pachucki; Shiv K. Verma
EPI_ISL_7568605, EPI_ISL_7568608	NYU Langone Health	Departments of Pathology and Medicine, New York University School of Medicine	Adriana Heguy; Christian Marier; Dacia Dimartino; Emily Guzman; Gael Westby; Guiqing Wang; Paul Zappile; Peter Meyn; Sitharam Ramaswami; Yutong Zhang
EPI_ISL_7285023	National Centre for Disease Control (NCDC) Biotechnology Division, Delhi	NCDC Delhi, Biotechnology Division INSACOG	Hema Gogia; Hemlata Lall; Kalaiarasan Ponnusamy; Mahesh S Dhar; Manoj K Singh; Meena Datta; Partha Rakshit; Preeti Madan; Priyanka Singh; Radhakrishnan V. S; Robin Marwal; Sandhya Kabra; Sujeet K Singh; Uma Sharma
EPI_ISL_7548907, EPI_ISL_7548908, EPI_ISL_7548911, EPI_ISL_7548912, EPI_ISL_7548913, EPI_ISL_7548915, EPI_ISL_7548916, EPI_ISL_7548918, EPI_ISL_7548919, EPI_ISL_7548920, EPI_ISL_7548921, EPI_ISL_7548922, EPI_ISL_7548924, EPI_ISL_7548925, EPI_ISL_7548926, EPI_ISL_7548927, EPI_ISL_7548928, EPI_ISL_7548929, EPI_ISL_7548930, EPI_ISL_7548932, EPI_ISL_7548933, EPI_ISL_7548934, EPI_ISL_7548935, EPI_ISL_7548936, EPI_ISL_7548937, EPI_ISL_7548938, EPI_ISL_7548939, EPI_ISL_7552700, EPI_ISL_7552701, EPI_ISL_7552702, EPI_ISL_7552703, EPI_ISL_7552704, EPI_ISL_7552705, EPI_ISL_7552706, EPI_ISL_7552707, EPI_ISL_7552708, EPI_ISL_7552709, EPI_ISL_7552710			
see above	National Health Laboratory	Botswana Harvard AIDS Institute Partnership, Plot 1836 North Ring Road, Princess Marina Hospital, Gaborone	Boitumelo Zuze; Botshelo Radibe; Dorcas Maruapula; Doreen Ditshwanelo; Joseph Makhema; Keoratile Ntshambiwa; Kgomotso Moruisi; Legodile Kooepile; Mosepele Mosepele; Mphaphi B. Mbulawa; Ontlametse T. Bareng; Pamela Smith-Lawrence; Roger Shapiro; Sefetogi Ramaologa; Shahin Lockman; Sikhulile Moyo; Simani Gaseitsiwe; Thongbotho Mphoyakgosi; Wonderful T. Choga
EPI_ISL_6795195, EPI_ISL_6795199, EPI_ISL_6795202, EPI_ISL_6795203, EPI_ISL_6795406, EPI_ISL_7015210, EPI_ISL_7015212, EPI_ISL_7015216, EPI_ISL_7015223, EPI_ISL_7015224, EPI_ISL_7015226, EPI_ISL_7015229, EPI_ISL_7015230, EPI_ISL_7310589, EPI_ISL_7310595, EPI_ISL_7310605, EPI_ISL_7310613, EPI_ISL_7310622, EPI_ISL_7310630, EPI_ISL_7310631, EPI_ISL_7310636, EPI_ISL_7310643, EPI_ISL_7310648, EPI_ISL_7310658, EPI_ISL_7310666, EPI_ISL_7310675, EPI_ISL_7310679, EPI_ISL_7310685, EPI_ISL_7310703, EPI_ISL_7310710, EPI_ISL_7310719, EPI_ISL_7310725, EPI_ISL_7310733, EPI_ISL_7310742, EPI_ISL_7310747, EPI_ISL_7358050, EPI_ISL_7358051, EPI_ISL_7358052, EPI_ISL_7358053, EPI_ISL_7358054, EPI_ISL_7358055, EPI_ISL_7358056, EPI_ISL_7358057, EPI_ISL_7358058, EPI_ISL_7358059, EPI_ISL_7358064, EPI_ISL_7358065, EPI_ISL_7358066, EPI_ISL_7358067, EPI_ISL_7358068, EPI_ISL_7358069, EPI_ISL_7358070, EPI_ISL_7358071, EPI_ISL_7358072, EPI_ISL_7358073, EPI_ISL_7358074, EPI_ISL_7358075, EPI_ISL_7358076, EPI_ISL_7358077, EPI_ISL_7358078, EPI_ISL_7358079, EPI_ISL_7358080, EPI_ISL_7358081, EPI_ISL_7358082, EPI_ISL_7358083, EPI_ISL_7358084, EPI_ISL_7358085, EPI_ISL_7358089, EPI_ISL_7358090, EPI_ISL_7358091, EPI_ISL_7358092, EPI_ISL_7358093, EPI_ISL_7381192, EPI_ISL_7381193, EPI_ISL_7381194, EPI_ISL_7381195, EPI_ISL_7381196, EPI_ISL_7381197, EPI_ISL_7381198, EPI_ISL_7381199, EPI_ISL_7381200, EPI_ISL_7381201, EPI_ISL_7381202, EPI_ISL_7381203, EPI_ISL_7381204, EPI_ISL_7381205, EPI_ISL_7381206, EPI_ISL_7381207, EPI_ISL_7545676, EPI_ISL_7545677, EPI_ISL_7545678, EPI_ISL_7545679, EPI_ISL_7545680, EPI_ISL_7545681, EPI_ISL_7545682, EPI_ISL_7545683, EPI_ISL_7545684, EPI_ISL_7545685, EPI_ISL_7545686, EPI_ISL_7545687, EPI_ISL_7545688, EPI_ISL_7545689, EPI_ISL_7545690, EPI_ISL_7545691, EPI_ISL_7545692, EPI_ISL_7545693, EPI_ISL_7545694, EPI_ISL_7545695, EPI_ISL_7545696, EPI_ISL_7545698, EPI_ISL_7545699, EPI_ISL_7545700, EPI_ISL_7545701, EPI_ISL_7545702, EPI_ISL_7545703, EPI_ISL_7545704, EPI_ISL_7545705, EPI_ISL_7545706, EPI_ISL_7545707, EPI_ISL_7545708, EPI_ISL_7545709, EPI_ISL_7545710, EPI_ISL_7545711, EPI_ISL_7545712, EPI_ISL_7545713, EPI_ISL_7545714, EPI_ISL_7545715, EPI_ISL_7545716, EPI_ISL_7545717, EPI_ISL_7545718, EPI_ISL_7545719, EPI_ISL_7545720, EPI_ISL_7545721, EPI_ISL_7545722, EPI_ISL_7545723, EPI_ISL_7545724, EPI_ISL_7545725, EPI_ISL_7545726, EPI_ISL_7545727, EPI_ISL_7545728, EPI_ISL_7545729, EPI_ISL_7545730, EPI_ISL_7545731, EPI_ISL_7545732, EPI_ISL_7545733, EPI_ISL_7545734, EPI_ISL_7545735, EPI_ISL_7545736, EPI_ISL_7545737, EPI_ISL_7545738, EPI_ISL_7545739, EPI_ISL_7545742, EPI_ISL_7545743, EPI_ISL_7545744, EPI_ISL_7545745, EPI_ISL_7545746, EPI_ISL_7545747, EPI_ISL_7545748, EPI_ISL_7545749, EPI_ISL_7545750, EPI_ISL_7545751, EPI_ISL_7545752, EPI_ISL_7545753, EPI_ISL_7545754, EPI_ISL_7545755, EPI_ISL_7545756, EPI_ISL_7545757, EPI_ISL_7545758, EPI_ISL_7545759, EPI_ISL_7545760, EPI_ISL_7545761, EPI_ISL_7545762, EPI_ISL_7545764, EPI_ISL_7545765, EPI_ISL_7545766, EPI_ISL_7545767, EPI_ISL_7545768, EPI_ISL_7545769, EPI_ISL_7545770, EPI_ISL_7545771, EPI_ISL_7545772, EPI_ISL_7545773, EPI_ISL_7545774, EPI_ISL_7545775, EPI_ISL_7545776, EPI_ISL_7545777, EPI_ISL_7545778, EPI_ISL_7545779, EPI_ISL_7545780, EPI_ISL_7545781, EPI_ISL_7545782, EPI_ISL_7545783, EPI_ISL_7545784, EPI_ISL_7545785, EPI_ISL_7545787, EPI_ISL_7545788, EPI_ISL_7545789, EPI_ISL_7545790, EPI_ISL_7545791, EPI_ISL_7545792, EPI_ISL_7545793, EPI_ISL_7545794			
see above	National Health Laboratory Service, Kwazulu-Natal, South Africa	CERI, Centre for Epidemic Response and Innvoation, Stellenbosch University and KRISP, KZN Research Innovation and Sequencing Platform, UKZN.	Arisha Maharaj; Giandhari J; Moir M; Naidoo Y; Nokukhanya Mdlalose; Pillay S; Ramphal U; Ramphal Y; San JE; Tegally H; Tshiabuila D; Wilkinson E; de Oliveira T; van Wyk S
EPI_ISL_6829557	National Health Laboratory Service, Kwazulu-Natal, South Africa	KRISP, KZn Research Innovation and Sequencing Platform	Arisha Maharaj; Giandhari J; Lessells R; Moir M; Naidoo Y; Nokukhanya M; Pillay S; Ramphal U; Ramphal Y; San JE; Tegally H; Tshiabuila D; Wilkinson E; de Oliveira T
EPI_ISL_6795188,	National Health Laboratory Services,	CERI, Centre for Epidemic Response	Arisha Maharaj; Florette Treurnicht; Giandhari J; Kathleen Subramoney; Naidoo Y; Pillay S; Ramphal U; Ramphal Y; San JE; Tegally H; Tshiabuila D; Wilkinson E; de Oliveira T

EPI_ISL_6795189, EPI_ISL_6795190, EPI_ISL_6795191, EPI_ISL_6795192, EPI_ISL_6795193	Virology	and Innvoation, Stellenbosch University and KRISP, KZN Research Innovation and Sequencing Platform, UKZN.	
EPI_ISL_6699728, EPI_ISL_6699729, EPI_ISL_6699730, EPI_ISL_6699731, EPI_ISL_6699732, EPI_ISL_6699733, EPI_ISL_6699734, EPI_ISL_6699735, EPI_ISL_6699736, EPI_ISL_6699737, EPI_ISL_6699738, EPI_ISL_6699739, EPI_ISL_6699740, EPI_ISL_6699741, EPI_ISL_6699742, EPI_ISL_6699743, EPI_ISL_6699744, EPI_ISL_6699745, EPI_ISL_6699746, EPI_ISL_6699747, EPI_ISL_6699748, EPI_ISL_6699749, EPI_ISL_6699750, EPI_ISL_6699751, EPI_ISL_6699752, EPI_ISL_6699753, EPI_ISL_6699754, EPI_ISL_6699755, EPI_ISL_6699756, EPI_ISL_6699757, EPI_ISL_6699758, EPI_ISL_6699759, EPI_ISL_6699760, EPI_ISL_6699761, EPI_ISL_6699762, EPI_ISL_6699763, EPI_ISL_6699764, EPI_ISL_6699765, EPI_ISL_6699766, EPI_ISL_6699767, EPI_ISL_6699768, EPI_ISL_6699769, EPI_ISL_6699770, EPI_ISL_6699771, EPI_ISL_6782043, EPI_ISL_6782055, EPI_ISL_6782056, EPI_ISL_6782071, EPI_ISL_6782079, EPI_ISL_6782080, EPI_ISL_6782084, EPI_ISL_6782090, EPI_ISL_6782092, EPI_ISL_6810482, EPI_ISL_6810483, EPI_ISL_6810484, EPI_ISL_6810485, EPI_ISL_6810486, EPI_ISL_6810487			
see above	National Health Laboratory Services, Virology, Charlotte Maxeke Johannesburg hospital, Parktown, Johannesburg, Gauteng	CERI, Centre for Epidemic Response and Innvoation, Stellenbosch University and KRISP, KZN Research Innovation and Sequencing Platform, UKZN.	Amoaka D; Arisha Maharaj; Avani Bharuthram; Bester P; Bhiman J; Engelbrecht S; Everatt J; Florette Treurnicht; Goedhals D; Hardie D; Hsiao M; Iranzadeh A; Kathleen Subramoney; Lessells R; Makatini Z; Maponga T; Mdlalose N; Mlisana K; Moir M; NGS-SA (Scheepers C; Naidoo Y; Nkhensani Mtileni; Nyaga M) Giandhari J; Oluwakemi M; Pillay S; Preiser W; Ramphal U; Ramphal Y; San JE; Tegally H; Tshiabuila D; Venter M; Wilkinson E; Williamson C; de Oliveira T; von Gottberg A
EPI_ISL_6939033, EPI_ISL_6939034, EPI_ISL_6939035, EPI_ISL_6939036, EPI_ISL_6939038, EPI_ISL_6939039, EPI_ISL_6939041, EPI_ISL_6939042, EPI_ISL_6939043, EPI_ISL_6939044, EPI_ISL_6939045, EPI_ISL_6939046, EPI_ISL_6939047, EPI_ISL_6939048, EPI_ISL_6939049, EPI_ISL_6939050, EPI_ISL_6939051, EPI_ISL_6939052, EPI_ISL_6939053, EPI_ISL_6939054, EPI_ISL_6939056, EPI_ISL_6939057, EPI_ISL_6939058, EPI_ISL_6939059, EPI_ISL_6939060, EPI_ISL_6939061, EPI_ISL_6939062, EPI_ISL_6939063, EPI_ISL_6939064, EPI_ISL_6939065, EPI_ISL_6939066, EPI_ISL_6939067, EPI_ISL_6939068, EPI_ISL_7661055, EPI_ISL_7661094, EPI_ISL_7661095, EPI_ISL_7661096, EPI_ISL_7661098			
see above	National Influenza Centre	National Influenza Centre	; Benjamiin B. Lindsey; Benjamin H. Foulkes; Bless Seyram Agbenyo; Bright Adu; Ernest Asiedu; Franklin Asiedu-Bekoe; Hilda Opoku Frempong; Ivy A. Asante; Joseph Oliver-Commey; Joyce Appiah-Kubi; Keren Okyerebea Attiku; Linda Boatemaa; Lorreta Kwasah; Mathew D. Parker; Michael Marks; Mildred Adusei-Poku; Quaneeta Mohktar; Sharon Hsu; Thushan I de Silva; William K. Ampofo
EPI_ISL_7456448, EPI_ISL_7456450, EPI_ISL_7456451, EPI_ISL_7456452, EPI_ISL_7456453, EPI_ISL_7456454, EPI_ISL_7456455, EPI_ISL_7456456, EPI_ISL_7456457			
see above	National Institute For Communicable Diseases Of The National Health Laboratory Service	National Institute for Communicable Diseases of the National Health Laboratory Service	Amoako DG; Bhiman JN; Everatt J; Ismail A; Mahlangu B; Mnguni A; Mohale T; Ntuli N; Scheepers C; Wolter N
EPI_ISL_7605585, EPI_ISL_7605586, EPI_ISL_7605593, EPI_ISL_7605594, EPI_ISL_7605629, EPI_ISL_7605630, EPI_ISL_7605631, EPI_ISL_7605632, EPI_ISL_7605637, EPI_ISL_7605638, EPI_ISL_7605666, EPI_ISL_7605720, EPI_ISL_7605759, EPI_ISL_7605762, EPI_ISL_7605765, EPI_ISL_7605767, EPI_ISL_7605768, EPI_ISL_7605770, EPI_ISL_7605771			
see above	National Institute for Communicable Diseases of the National Health Laboratory Service	National Institute for Communicable Diseases of the National Health Laboratory Service	Amoako DG; Bhiman JN; Everatt J; Ismail A; Mahlangu B; Mnguni A; Mohale T; Ntuli N; Scheepers C; Wolter N
EPI_ISL_7418017, EPI_ISL_7571617, EPI_ISL_7571618	National Institute of Infectious Diseases	National Institute of Infectious Diseases	Harutaka Katano; Ken Maeda; Kentaro Itokawa; Makoto Kuroda; Shuetsu Fukushi; Shun Iida; Tadaki Suzuki; Tsuyoshi Sekizuka; Yudai Kuroda
EPI_ISL_7021517, EPI_ISL_7074135	National Platform bis COVID ULB-IBC	National Platform bis COVID ULB-IBC	Arnaud Marchant; Benoit Haerlingen; Coralie Henin; Marie-Luce Delforge; Ricardo De Mendonça
EPI_ISL_7063764, EPI_ISL_7288348, EPI_ISL_7288357, EPI_ISL_7288375, EPI_ISL_7544936, EPI_ISL_7544937, EPI_ISL_7544938, EPI_ISL_7544939, EPI_ISL_7544940, EPI_ISL_7544941, EPI_ISL_7544942, EPI_ISL_7544947, EPI_ISL_7544948, EPI_ISL_7544949, EPI_ISL_7544950, EPI_ISL_7544953, EPI_ISL_7544954, EPI_ISL_7544956, EPI_ISL_7544960, EPI_ISL_7544963, EPI_ISL_7544964, EPI_ISL_7544965, EPI_ISL_7544969, EPI_ISL_7544971, EPI_ISL_7544972, EPI_ISL_7544974, EPI_ISL_7544975, EPI_ISL_7544980, EPI_ISL_7651290, EPI_ISL_7651294, EPI_ISL_7651298, EPI_ISL_7651301, EPI_ISL_7651303, EPI_ISL_7651310, EPI_ISL_7651313, EPI_ISL_7651315			
see above	National Platform bis UMONS/Jolimont	National Platform bis UMONS/Jolimont	Eric Tarantino; Florian Juszczak; Gautier Detry; Guillaume Bayon-Vicente; Laetitia Gheysen; Ruddy Wattiez
EPI_ISL_7137310, EPI_ISL_7137311, EPI_ISL_7195620, EPI_ISL_7195621, EPI_ISL_7195622, EPI_ISL_7195623, EPI_ISL_7460338, EPI_ISL_7604552, EPI_ISL_7604594, EPI_ISL_7604595, EPI_ISL_7604613, EPI_ISL_7604615, EPI_ISL_7604627			
see above	National Public Health Laboratory, National Centre for Infectious Diseases	National Public Health Laboratory, National Centre for Infectious Diseases	Benny Yeo Ken Yee; Constance Chen; Dennis Loy Song Qi; Dimitar Kenanov; Grace Ngan Jie Yin; Katherine Ching; Katherine Ching Zi Yan; Kwan Ki Ko; Lin Cui; Mak Tze Minn; Niranjan Nagarajan; Raymond Tzer Pin Lin; Royce Ang; Samuel Loo; Sebastian Maurer Stroh; Suphavilai Chayaporn; Zhenyang Zhou
EPI_ISL_7154394, EPI_ISL_7154403, EPI_ISL_7273097, EPI_ISL_7547734, EPI_ISL_7547735	National Reference Laboratory, NCDC	National Reference Laboratory, Nigeria Centre for Disease Control	Catherine Okoi; Chimaobi Chukwu; Dr Ifedayo Adetifa; Dr Ndodo Nnaemeka; Dr Omoare Adesuyi; Nwando Mba; Olajumoke Babatunde; Olusola Anuoluwapo Akanbi; Oyeronke Ayansola
EPI_ISL_6939819, EPI_ISL_7354124, EPI_ISL_7406048, EPI_ISL_7406049, EPI_ISL_7406082, EPI_ISL_7406083	National Virus Reference Laboratory	National Virus Reference Laboratory	Charlene Bennett; Cillian F De Gascun; Gabriel Gonzalez; Jonathan Dean; Michael Carr; Zoe Yandle
EPI_ISL_7616432, EPI_ISL_7616434, EPI_ISL_7616435, EPI_ISL_7616437, EPI_ISL_7616439, EPI_ISL_7616441, EPI_ISL_7616442			
see above	Naval Health Clinic Annapolis	Naval Medical Research Center Biological Defense Research Directorate	Catherine E. Arnold; Francisco Malagon; Kimberly A. Bishop-Lilly; Kyle A. Long; Logan J. Voegtly; Megan A. Schilling; Regina Z. Cer
EPI_ISL_7605624, EPI_ISL_7605625, EPI_ISL_7605626, EPI_ISL_7605627, EPI_ISL_7605628	Ndlovu Reaserch Centre	National Institute for Communicable Diseases of the National Health Laboratory Service	Amoako DG; Bhiman JN; Everatt J; Ismail A; Mahlangu B; Mnguni A; Mohale T; Ntuli N; Scheepers C; Wolter N
EPI_ISL_7116413, EPI_ISL_7116439, EPI_ISL_7116461, EPI_ISL_7116681, EPI_ISL_7116718, EPI_ISL_7116738	Nebraska Public Health Laboratory	NPHL COVID-19 Response Team	NPHL COVID-19 Response Team
EPI_ISL_7544379, EPI_ISL_7544739	New Somerset Hospital wc NSH	NHLS/UCT	Arash Iranzadeh; Bruna Galvao; Carolyn Williamson; Deelan Doolabh; Diana Hardie; Gert Marais; Innocent Mudau; Luicer Olubayo; Lynn Tyers; Marvin Hsiao; Nokuzola Mbhele; Rageema Joseph; Stephen Korsman
EPI_ISL_6814922, EPI_ISL_6814923, EPI_ISL_6829575, EPI_ISL_6829577, EPI_ISL_6958955, EPI_ISL_7162071, EPI_ISL_7162072, EPI_ISL_7162073, EPI_ISL_7265843, EPI_ISL_7379517, EPI_ISL_7379527, EPI_ISL_7457536, EPI_ISL_7457537, EPI_ISL_7457538, EPI_ISL_7503742, EPI_ISL_7503743, EPI_ISL_7503744, EPI_ISL_7503745, EPI_ISL_7503746, EPI_ISL_7551964, EPI_ISL_7551971, EPI_ISL_7569451, EPI_ISL_7569452, EPI_ISL_7569453, EPI_ISL_7622408, EPI_ISL_7622409, EPI_ISL_7622410, EPI_ISL_7622411, EPI_ISL_7622412			
see above	New South Wales Health Pathology Royal Prince Alfred Hospital	Microbiology RPAH	Au, J.; Bull, R.; Deveson, I.; Foster, C.; Rawlinson, W.; Ruiz Silva, M.; Van Hal, S.
EPI_ISL_7418387, EPI_ISL_7431953	Niedersächsisches Landesgesundheitsamt (NLGA)	Robert Koch Institute	
EPI_ISL_6958280, EPI_ISL_7571541, EPI_ISL_7571542	North Lantau Hospital	Hong Kong Department of Health	Alan K.L. Tsang; Edman T.K. Lam; Ken H.L. Ng; Patricia K. L. Leung; Peter C.W. Yip; Rickjason C.W. Chan
EPI_ISL_7601238, EPI_ISL_7601282, EPI_ISL_7601310, EPI_ISL_7614981	OKMI	University Hospital Brno, CMBG	Bezdicek Matej; Dolejska Monika; Kristyna Dufkova; Lengerova Martina; Svaton Jan
EPI_ISL_7334887, EPI_ISL_7334888	ONEIDA COUNTY HEALTH DEPT.	Wadsworth Center, New York State Department of Health	Alexis Russell; Catharine Prussing; Daryl M. Lamson; Erasmus Schneider; Erica Lasek-Nesselquist; John Kelly; Jonathan Plitnick; Kirsten St. George; Matthew Shudt; Melissa A Leisner; Navjot Singh
EPI_ISL_7552440, EPI_ISL_7552446	Ochsner Health	BioInfoExperts	Amy Feehan; Ben Lain; Chris Huston; David J. Nolan; Judy Crabtree; Julia-Garcia-Diaz; Lucio Miele; Rebecca Rose; Samual Moot; Susanna L. Lamers; Tessa LaFleur
EPI_ISL_7495763	Omega Diagnostics at Mounes	Omega Diagnostics at Mounes	Cherish Jackson; Cynthia Corley; Latira Haynes-Jacob; MD; Vivek Khare
EPI_ISL_7040235	Oslo University Hospital, Department of Medical Microbiology	Norwegian Institute of Public Health, Department of Virology	Atiya R Ali; Debech Nadia; Engebretsen Serina Beate; Garcia Llorente Ignacio; Hilde Elshaug; Hilde Vollan; Jon Bråte; Kamilla Heddeland Instefjord; Karoline Bragstad; Kathrine Stene-Johansen; Line Victoria Moen; Marie Paulsen Madsen; Olav Hungnes; Pedersen Benedikte Nevjen; Rasmus Riis Kopperud
EPI_ISL_7248730, EPI_ISL_7248745, EPI_ISL_7248753, EPI_ISL_7248763, EPI_ISL_7470724, EPI_ISL_7470728	Oslo University Hospital, Department of Microbiology	Norwegian Institute of Public Health, Department of Virology	Arvind Yegambaram Meenakshi Sundaram; Atiya R Ali; Cathrine Fladeby; Debech Nadia; Engebretsen Serina Beate; Garcia Llorente Ignacio; Gregor D. Gilfillan; Hilde Elshaug; Hilde Vollan; Jon Bråte; Kamilla Heddeland Instefjord; Karoline Bragstad; Kathrine Stene-Johansen; Line Victoria Moen; Lise Andresen; Mariann Nilsen; Marie Paulsen Madsen; Mona Holberg-Petersen; Olav Hungnes; Pedersen Benedikte Nevjen; Pål Marius Bjørnstad; Rasmus Riis Kopperud; Teodora Plamenova Ribarska
EPI_ISL_7470189	Ostfold Hospital Trust - Kalnes, Centre for Laboratory Medicine, Section for gene technology and infection serology	Norwegian Institute of Public Health, Department of Virology	Atiya R Ali; Debech Nadia; Engebretsen Serina Beate; Garcia Llorente Ignacio; Hilde Elshaug; Hilde Vollan; Jon Bråte; Kamilla Heddeland Instefjord; Karoline Bragstad; Kathrine Stene-Johansen; Line Victoria Moen; Marie Paulsen Madsen; Olav Hungnes; Pedersen Benedikte Nevjen; Rasmus Riis Kopperud

EPI_ISL_7467356	PHV-FSS	PHV-FSS	Chenwei Wang on behalf of Q-PHIRE Genomics
EPI_ISL_7605742, EPI_ISL_7605764	PORT ELIZABETH LABORATORY	National Institute for Communicable Diseases of the National Health Laboratory Service	Amoako DG; Bhiman JN; Everatt J; Ismail A; Mahlangu B; Mnguni A; Mohale T; Ntuli N; Scheepers C; Wolter N
EPI_ISL_7605607	POTCHEFSTROOM LABORATORY	National Institute for Communicable Diseases of the National Health Laboratory Service	Amoako DG; Bhiman JN; Everatt J; Ismail A; Mahlangu B; Mnguni A; Mohale T; Ntuli N; Scheepers C; Wolter N
EPI_ISL_6774082, EPI_ISL_6774086, EPI_ISL_6774092	Palapye Primary Hospital Laboratory	Botswana Harvard HIV Reference Laboratory	Boitumelo Zuze; Botshelo Radibe; Dorcas Maruapula; Joseph Makhema; Keoratile Ntshambiwa; Kgomotso Moruisi; Legodile Kooepile; Mosepele Mosepele; Mphaphi B. Mbulawa; Ontlametse T. Bareng; Pamela Smith-Lawrence; Roger Shapiro; Sefetogi Ramaologa; Shahin Lockman; Sikhulile Moyo; Simani Gaseitsiwe; Thongbotho Mphoyakgosi; Wonderful T. Choga
EPI_ISL_7129868, EPI_ISL_7129869, EPI_ISL_7602801, EPI_ISL_7604813, EPI_ISL_7604824, EPI_ISL_7614032, EPI_ISL_7614035, EPI_ISL_7614040			
see above	Pandemic Response Lab - NYC	Pandemic Response Lab, R&D	Alex Carpio; Cybill del Castillo; Dylan Law; Haiping Hao; Henry Lee; Isabel Fernandez Escapa; Jon Laurent; Melissa Hopkins; Michael Hammerling; Pradeep Bugga; Shinyoung Clair Kang; Sol Rey; William Ward
EPI_ISL_6842152, EPI_ISL_6842154, EPI_ISL_6842155, EPI_ISL_6842157, EPI_ISL_6842158, EPI_ISL_6842160, EPI_ISL_6842161, EPI_ISL_6842164, EPI_ISL_6842166, EPI_ISL_6842167, EPI_ISL_7452739, EPI_ISL_7452740, EPI_ISL_7452743, EPI_ISL_7452747, EPI_ISL_7452752, EPI_ISL_7452753, EPI_ISL_7452754, EPI_ISL_7452755, EPI_ISL_7452756, EPI_ISL_7452757, EPI_ISL_7452759, EPI_ISL_7452760, EPI_ISL_7452779, EPI_ISL_7452784, EPI_ISL_7452786, EPI_ISL_7452787, EPI_ISL_7452788, EPI_ISL_7452789, EPI_ISL_7452790, EPI_ISL_7452791, EPI_ISL_7452801, EPI_ISL_7452802, EPI_ISL_7452803, EPI_ISL_7452804, EPI_ISL_7456466, EPI_ISL_7456467, EPI_ISL_7456468, EPI_ISL_7456469, EPI_ISL_7456470, EPI_ISL_7456471, EPI_ISL_7456472, EPI_ISL_7456473, EPI_ISL_7456474, EPI_ISL_7456475, EPI_ISL_7456476, EPI_ISL_7456477, EPI_ISL_7456478, EPI_ISL_7456479, EPI_ISL_7456480, EPI_ISL_7456481, EPI_ISL_7456482, EPI_ISL_7456483, EPI_ISL_7456484, EPI_ISL_7456485, EPI_ISL_7456489, EPI_ISL_7456490, EPI_ISL_7456491, EPI_ISL_7456492, EPI_ISL_7456493, EPI_ISL_7456494, EPI_ISL_7456495, EPI_ISL_7456496, EPI_ISL_7456497, EPI_ISL_7456498, EPI_ISL_7456499, EPI_ISL_7456500, EPI_ISL_7456501, EPI_ISL_7456502, EPI_ISL_7456503, EPI_ISL_7456504, EPI_ISL_7456505, EPI_ISL_7456506, EPI_ISL_7456507, EPI_ISL_7456508, EPI_ISL_7456509, EPI_ISL_7456510, EPI_ISL_7456511, EPI_ISL_7456512, EPI_ISL_7456513, EPI_ISL_7456514, EPI_ISL_7456515, EPI_ISL_7456516, EPI_ISL_7456517, EPI_ISL_7456518, EPI_ISL_7456519, EPI_ISL_7456520, EPI_ISL_7456521, EPI_ISL_7456522, EPI_ISL_7456523, EPI_ISL_7456524, EPI_ISL_7544906, EPI_ISL_7544907, EPI_ISL_7544908, EPI_ISL_7544909, EPI_ISL_7544910, EPI_ISL_7544911, EPI_ISL_7544912, EPI_ISL_7544913, EPI_ISL_7544914, EPI_ISL_7544915, EPI_ISL_7544916, EPI_ISL_7544917, EPI_ISL_7544918, EPI_ISL_7544919, EPI_ISL_7544920, EPI_ISL_7544921, EPI_ISL_7544922, EPI_ISL_7544923, EPI_ISL_7544924, EPI_ISL_7544925, EPI_ISL_7544926, EPI_ISL_7544927, EPI_ISL_7544928			
see above	PathCare, Cape Town	Division of Medical Virology, National Health Laboratory Service (NHLS), Tygerberg Hospital / Stellenbosch University	Gert van Zyl; Jean Maritz; Kamela Mahlakwane; Nadine Cronje; Petra Raimond; Shannon Wilson; Tania Stander; Tongai Maponga; Wolfgang Preiser
EPI_ISL_7263932, EPI_ISL_7263933	Pathogenic Microorganisms Variability Laboratory	Pathogenic Microorganisms Variability Laboratory	Alexander Gintsburg; Alexander Voskoboinikov; Andrei Siniavin; Andrey Pochtovyy; Artem Tkachuk; Denis Logunov; Elena Shidlovskaya; Elizaveta Divisenko; Inna Dolzhikova; Lyudmila Vasilchenko; Nadezhda Kuznetsova; Odintsova Alina; Vladimir Gushchin
EPI_ISL_7621339	Pathology North - Gosford Hospital - NSW Health Pathology	NSW Health Pathology - Institute of Clinical Pathology and Medical Research; Westmead Hospital; University of Sydney	Arnott A.; Draper J.; Gall M.; Martinez E.; Rockett R.; Sintchenko V.; on behalf of ICPMR
EPI_ISL_7265083, EPI_ISL_7265084	Pathology North - Royal North Shore Hospital - NSW Health Pathology	NSW Health Pathology - Institute of Clinical Pathology and Medical Research; Westmead Hospital; University of Sydney	Arnott A.; Draper J.; Gall M.; Martinez E.; Rockett R.; Sintchenko V.; on behalf of ICPMR
EPI_ISL_6864915, EPI_ISL_6956011, EPI_ISL_6956014, EPI_ISL_7620963, EPI_ISL_7621211, EPI_ISL_7621359, EPI_ISL_7621961			
see above	Pathology West - NSW Health Pathology	NSW Health Pathology - Institute of Clinical Pathology and Medical Research; Westmead Hospital; University of Sydney	Arnott A.; Draper J.; Gall M.; Martinez E.; Rockett R.; Sintchenko V.; on behalf of ICPMR
EPI_ISL_7565646	Pathology and Laboratory Medicine Institute, Cleveland Clinic, Ohio, USA	Pathology and Laboratory Medicine Institute, Cleveland Clinic, Ohio, USA	Concetta Peck; Daniel H. Farkas; Daniel Rhoads; David Bosler; David Plunkett; Jay Brock; Jennifer Starbuck; Jessica Spildener; Joy Nakitandwe; Kristen McDonnell; Nicole Hamon; Thomas Rose; Yu-Wei Cheng; Zheng Jin Tu
EPI_ISL_7406251, EPI_ISL_7472848, EPI_ISL_7472859, EPI_ISL_7472865	Plateforme de testing Namuroise	Plateforme de testing Namuroise	Degosserie Jonathan; Demars Aurore; Denis Olivier; Gilliard Nicolas; Maschietto Céline; Mullier François; Nobis Chloé; Otto Gaetan
EPI_ISL_7173899, EPI_ISL_7566194, EPI_ISL_7606105, EPI_ISL_7606106, EPI_ISL_7606107, EPI_ISL_7606108	Platform BIS UZA/UAntwerpen	Labo Klinische Biologie, UZA	Basil Britto Xavier; Christine Lammens; Herman Goossens; Ines Verbesselt; Jasmine Coppens; Kathleen Holemans; Marie Le Mercier; Veerle Matheeussen
EPI_ISL_6777160	Policlinico San Donato	Laboratory of Clinical Microbiology, Virology and Bioemergencies, ASST Fatebenefratelli Sacco - Sacco Hospital	Valeria Micheli
EPI_ISL_7416687, EPI_ISL_7416708	Procomcure Biotech Germany GmbH	Robert Koch Institute	
EPI_ISL_7507116, EPI_ISL_7507117, EPI_ISL_7507119	Public Health Authority of the Slovak Republic	Public Health Authority of the Slovak Republic	Anna Gičová; Barbora Kotvasová; Elena Tichá; Lucia Ševčíková; Miroslav Böhmer; Pavol Mišenko; Terézia Vrabľová; Tomáš Szemes
EPI_ISL_7135501, EPI_ISL_7135502, EPI_ISL_7135503, EPI_ISL_7135504, EPI_ISL_7263924, EPI_ISL_7263925, EPI_ISL_7263926, EPI_ISL_7263927, EPI_ISL_7263928, EPI_ISL_7263929, EPI_ISL_7263930, EPI_ISL_7334884, EPI_ISL_7334885, EPI_ISL_7334886, EPI_ISL_7438844, EPI_ISL_7438882, EPI_ISL_7438901, EPI_ISL_7438918, EPI_ISL_7438932, EPI_ISL_7438964, EPI_ISL_7438994, EPI_ISL_7459993, EPI_ISL_7459994, EPI_ISL_7459995, EPI_ISL_7459996, EPI_ISL_7459997, EPI_ISL_7459998, EPI_ISL_7495449, EPI_ISL_7495450, EPI_ISL_7495451, EPI_ISL_7495452, EPI_ISL_7495453, EPI_ISL_7495454, EPI_ISL_7495455, EPI_ISL_7620050, EPI_ISL_7620051, EPI_ISL_7620052, EPI_ISL_7620053, EPI_ISL_7620054, EPI_ISL_7620055, EPI_ISL_7620056			
see above	Public Health Laboratory, Public Health Service Amsterdam, The Netherlands	Department of Medical Microbiology & Infection prevention, Amsterdam University Medical Centers location AMC	Akke Cornelissen; Fokla Zorgdrager; Janke Schinkel; Jelle Koopsen; Judith den Uil; Marcel Jonges; Matthijs Welkers; Menno de Jong; Robin van Houdt; Sebastien Matamoros; Sjoerd Rebers; Sylvia Bruisten; Tjalling Leenstra and Mariken van der Lubben on behalf of the Amsterdam Regional Genomic epidemiology and Outbreak Surveillance (ARGOS) consortium
EPI_ISL_7135499, EPI_ISL_7259732, EPI_ISL_7259741, EPI_ISL_7259744, EPI_ISL_7334879, EPI_ISL_7334880, EPI_ISL_7464493, EPI_ISL_7464494, EPI_ISL_7613101, EPI_ISL_7613160, EPI_ISL_7613179, EPI_ISL_7613185, EPI_ISL_7613224, EPI_ISL_7613230, EPI_ISL_7613235, EPI_ISL_7613242			
see above	Public Health Ontario Laboratory	Public Health Ontario Laboratory	Aimin Li; Alireza Eshaghi; Andre Villegas; Ashleigh Sullivan; Christine Frantz; Dean Maxwell; Esha Joshi; Jared Simpson; Jennifer L Guthrie; Jonathan B Gubbay; Karthikeyan Sivaraman; Lawrence Heisler; Matthew Watson; Michael CY Li; Michael Laszloffy; Nahuel Fittipaldi; Philip Banh; Richard de Borja; Samir N Patel; Sandeep Nagra; Sandra Zittermann; Sarah Teatero; Vanessa G Allen; Yao Chen; Yogi Sundaravadanam
EPI_ISL_7592963	Puerto Rico Department of Health	Centers for Disease Control and Prevention, Dengue Branch	Betzabel Flores; Gabriela Paz-Bailey; Gilberto A. Santiago; Glenda Gonzalez; Jorge L. Munoz-Jordan; Keyla Charriez
EPI_ISL_7605651, EPI_ISL_7605652, EPI_ISL_7605653, EPI_ISL_7605654, EPI_ISL_7605655, EPI_ISL_7605656, EPI_ISL_7605658, EPI_ISL_7605659, EPI_ISL_7605660, EPI_ISL_7605661, EPI_ISL_7605662, EPI_ISL_7605663, EPI_ISL_7605664, EPI_ISL_7605667, EPI_ISL_7605668, EPI_ISL_7605671, EPI_ISL_7605672, EPI_ISL_7605673, EPI_ISL_7605674, EPI_ISL_7605675, EPI_ISL_7605679, EPI_ISL_7605682, EPI_ISL_7605685, EPI_ISL_7605704, EPI_ISL_7605738, EPI_ISL_7605751, EPI_ISL_7605773, EPI_ISL_7605776, EPI_ISL_7605777, EPI_ISL_7605778			
see above	ROB FERREIRA LABORATORY	National Institute for Communicable Diseases of the National Health Laboratory Service	Amoako DG; Bhiman JN; Everatt J; Ismail A; Mahlangu B; Mnguni A; Mohale T; Ntuli N; Scheepers C; Wolter N
EPI_ISL_7605676, EPI_ISL_7605740	RUSTENBURG LABORATORY	National Institute for Communicable Diseases of the National Health Laboratory Service	Amoako DG; Bhiman JN; Everatt J; Ismail A; Mahlangu B; Mnguni A; Mohale T; Ntuli N; Scheepers C; Wolter N
EPI_ISL_6862005	Regional Hospital Liberec	Regional Hospital Liberec	Iva Dolinova; Katerina Arientova; Katerina Stillerova; Martin Kracik; Tomas Zajic
EPI_ISL_7166216, EPI_ISL_7345201, EPI_ISL_7345221, EPI_ISL_7345322, EPI_ISL_7398995, EPI_ISL_7399058, EPI_ISL_7399073, EPI_ISL_7399078, EPI_ISL_7399512, EPI_ISL_7400550, EPI_ISL_7400551, EPI_ISL_7400555, EPI_ISL_7463952, EPI_ISL_7463953, EPI_ISL_7463956, EPI_ISL_7463961, EPI_ISL_7463968, EPI_ISL_7463969, EPI_ISL_7463972, EPI_ISL_7463975, EPI_ISL_7463979, EPI_ISL_7463997, EPI_ISL_7464020, EPI_ISL_7464030, EPI_ISL_7464048, EPI_ISL_7464049, EPI_ISL_7464059, EPI_ISL_7509826, EPI_ISL_7509828, EPI_ISL_7509830, EPI_ISL_7563604, EPI_ISL_7563965, EPI_ISL_7563969, EPI_ISL_7583283, EPI_ISL_7583286, EPI_ISL_7583295, EPI_ISL_7583354, EPI_ISL_7583375, EPI_ISL_7583411, EPI_ISL_7637839, EPI_ISL_7637844, EPI_ISL_7637845			
see above	Respiratory Virus Unit, Microbiology Services Colindale, Public Health England	COVID-19 Genomics UK (COG-UK) Consortium	PHE Covid Sequencing Team
EPI_ISL_7116918	Robert Koch-Institut ZBS1 (Zentrum für biologische Gefahren und spezielle Pathogene hochpathogene Viren)	Robert Koch Institute	
EPI_ISL_7142714, EPI_ISL_7303373, EPI_ISL_7485193, EPI_ISL_7519924, EPI_ISL_7536363, EPI_ISL_7656186	Rosalind Franklin Laboratory	Wellcome Sanger Institute for the COVID-19 Genomics UK (COG-UK) Consortium	Cordelia Langford; David K. Jackson; Dominic Kwiatkowski; Donald Fraser; Ewan Harrison; Ian Johnston; Jeffrey Barrett; John Sillitoe on behalf of the Wellcome Sanger Institute COVID-19 Surveillance Team; Rob Howes; Roberto Amato; Sonia Goncalves; Suki Lee; The Rosalind Franklin Laboratory and Alex Alderton
EPI_ISL_7366154	Rush University Medical Center	RIPHL at Rush University Medical Center	Alyse Kittner; Cecilia Chau; Diane Springer; Edith Perez; Felix Araujo Perez; Hannah Barbian; Joyce Houlihan; Kevin Kunstman; Laura Furtado; Marieta Hyde; Mary Hayden; Sofiya Bobrovska; Stefan Green
EPI_ISL_7545637, EPI_ISL_7545638, EPI_ISL_7545639, EPI_ISL_7545640, EPI_ISL_7545641, EPI_ISL_7545642, EPI_ISL_7545643, EPI_ISL_7545644, EPI_ISL_7545645, EPI_ISL_7545646, EPI_ISL_7545647, EPI_ISL_7545648, EPI_ISL_7545649, EPI_ISL_7545650			

see above	SAMRC	CERI, Centre for Epidemic Response and Innvoation, Stellenbosch University and KRISP, KZN Research Innovation and Sequencing Platform, UKZN.	Arisha Maharaj; Giandhari J; MRC; Naidoo Y; Pillay S; Ramphal U; Ramphal Y; San JE; Tegally H; Tshiabuila D; Wilkinson E; de Oliveira T
EPI_ISL_6913953, EPI_ISL_6914908, EPI_ISL_7194610	SARS-CoV-2 testing team, National Institute of Infectious Diseases	Pathogen Genomics Center, National Institute of Infectious Diseases	Hazuka Y Furihata; Kentaro Itokawa; Makoto Kuroda; Masanori Hashino; Masumichi Saito; Naomi Nojiri; Nozomu Hanaoka; Rina Tanaka; Tsuguto Fujimoto; Tsuyoshi Sekizuka
EPI_ISL_7458718, EPI_ISL_7458719, EPI_ISL_7458720, EPI_ISL_7458721, EPI_ISL_7659355	SK-Roy Romanow Provincial Laboratory	Saskatchewan - Roy Romanow Provincial Laboratory (RRPL)	Alanna Senecal; Amanda Lang; Jessica Minion; Kara Loos; Keith MacKenzie; Meredith Faires; Rachel DePaulo; Roy Romanow Provincial Laboratory - Molecular Diagnostics; Ryan McDonald
EPI_ISL_7334889, EPI_ISL_7334890	SUNRISE MEDICAL LABORATORIES	Wadsworth Center, New York State Department of Health	Alexis Russell; Catharine Prussing; Daryl M. Lamson; Erasmus Schneider; Erica Lasek-Nesselquist; John Kelly; Jonathan Plitnick; Kirsten St. George; Matthew Shudt; Melissa A Leisner; Navjot Singh
EPI_ISL_7195727	SYNLAB	GIGA Medical Genomics	Bouchra Boujemla; Claire Gourzonès; Cécile Meex; Keith Durkin; Laurent Gillet; Maria Artesi; Marie-Pierre Hayette; Nadine Cambisano; Nathalie Renotte; Olivier Ek; Sébastien Bontems; Vincent Bours
EPI_ISL_7440440, EPI_ISL_7442466	SYNLAB MVZ Weiden	Robert Koch Institute	
EPI_ISL_7565723	Salud Digna	Instituto Nacional de Medicina Genomica	Abraham Campos-Romero; Cedro-Tanda A; Cruz-Islas Jazmin; Escobar-Arrazola MA; Garnica-Lopez Dora; Herrera-Montalvo LA.; Hidalgo-Miranda A; Luna-Ruiz Marco; Mendoza-Vargas A; Moreno-Camacho José Luis; Ramirez-Vega O; Rangel-DeLeon D; Reyes-Grajeda JP; Rodriguez-Gallegos Jorge; Yair Alfaro-Mora
EPI_ISL_7200823	Salzkammergutklinikum Vöcklabruck, Institut für Pathologie	Salzkammergutklinikum Vöcklabruck, Institut für Pathologie	Franz Pühringer; Penka Lechner; Regina Stitz; René Silye; Senka Rohregger
EPI_ISL_7657583, EPI_ISL_7657589	San Diego County Public Health Laboratory	Andersen lab at Scripps Research	SEARCH Alliance San Diego
EPI_ISL_7015235	Selangor State Health Department	Institute for Medical Research, Infectious Disease Research Centre, National Institutes of Health, Ministry of Health Malaysia	Ahmad FA; Ahmad Fazilah NA; Anasir MI; Azizan MA; Kamel K; Mohamad Sukri MZ; Mohd Zawawi Z; Norhisham SN; Ramly N; Robert F; Rosli NR; Suppiah J; Thayan R
EPI_ISL_7467969	Servicio Virosis Respiratorias-Departamento Virología-INEI	Instituto Nacional Enfermedades Infecciosas C.G.Malbran	Avaro M.; Baumeister E.; Benedetti E.; Campos J.; Cisterna D.; Dattero ME; De Belder D.; Haim MS.; Lorenzo F.; Molina V.; Perandones C.; Poklepovich T.; Pontoriero A.; Russo M.; Sanchez Loria J.; Tuduri E.
EPI_ISL_7625809	Servicio de Microbiologia Hospital Ramon y Cajal	Servicio de Microbiologia Hospital Ramon y Cajal	Galan JC; Martinez L. Abreu M; Ponce M; y Gonzalez-Alba JM
EPI_ISL_6795212, EPI_ISL_6825365, EPI_ISL_7056045, EPI_ISL_7056614, EPI_ISL_7593654, EPI_ISL_7593690, EPI_ISL_7593776, EPI_ISL_7593892, EPI_ISL_7594549, EPI_ISL_7594591, EPI_ISL_7594719, EPI_ISL_7594860, EPI_ISL_7595155, EPI_ISL_7595730, EPI_ISL_7595974, EPI_ISL_7596005, EPI_ISL_7596893, EPI_ISL_7596897, EPI_ISL_7596904, EPI_ISL_7596918, EPI_ISL_7596923, EPI_ISL_7596932, EPI_ISL_7596941, EPI_ISL_7596950, EPI_ISL_7596956, EPI_ISL_7596957, EPI_ISL_7596962, EPI_ISL_7596969, EPI_ISL_7596978, EPI_ISL_7596990, EPI_ISL_7597002, EPI_ISL_7603138, EPI_ISL_7603139, EPI_ISL_7604317, EPI_ISL_7604355, EPI_ISL_7604363, EPI_ISL_7613573, EPI_ISL_7614184, EPI_ISL_7614185, EPI_ISL_7614286, EPI_ISL_7614287			
see above	Shamir Medical Center (Asaf Harofe)	Shamir Medical Center (Asaf Harofe)	Abu Hamad Ramzia; Adina Bar Chaim; Alona Frenkel; Anna Vishnevsky; Chen Weiner; Nir Rainy; Patricia Benveniste-Lekovitz; Reut Sorek Abramovich; Yevgeni Yegorov
EPI_ISL_7062525, EPI_ISL_7405371	Spital Riggisberg	Institute for Infectious Diseases, University of Bern	Alban Ramette; Christian Baumann; Cora Sägesser; Franziska Suter-Riniker; Loïc Borcard; Miguel A Terrazos Miani; Nicole Liechti; Pascal Bittel; Peter Keller; Sonja Gempeler; Stefan Neuenschwander; Stephen L Leib
EPI_ISL_6963509	Sri Jayadeva Institute of Cardiovascular Sciences and Research / Strand Life Sciences	National Centre for Biological Sciences, TIFR - Rockefeller Foundation	Aarati Karaba; Anson Kunjumon George; Aparnaa Ramanathan; Apurva Sarin; Chandrasekhar Vadlamudi; Chitra Pattabiraman; Darshan Sreenivas; Dasaradhi Palakodeti; Dimple Notani; Divya Priya A; Madhusudhan J; Manisha Bharadwaj; Manoj Kumar Jha; Mudasir Nazaar; Pradeep B P; Priyanka Ananta Mulay; Ramesh Hariharan; Rohan Pais; Satyajit Mayor; Saumitra Mardikar; Srivathsan Adimoolam; Uma Ramakrishnan; Vamsi Veeramachaneni; Vasanthapuram Ravi; Vijay Chandru; Vishal G Rao; Yasodha Kannan
EPI_ISL_7265233	St Vincent's Pathology (SydPath)	NSW Health Pathology - Institute of Clinical Pathology and Medical Research; Westmead Hospital; University of Sydney	Arnott A.; Draper J.; Gall M.; Martinez E.; Rockett R.; Sintchenko V.; on behalf of ICPMR
EPI_ISL_7170972	Stadtspital Triemli	Institute of Medical Virology	Alexandra Trkola; Annette Audigé; Cyril Shah; Gabriela Ziltener; Guido Bloemberg; Jon Huder; Jürg Böni; Kevin Steiner; Maria Grünberg; Maryam Zaheri; Michael Huber; Riccarda Capaul; Stefan Schmutz; Verena Kufner
EPI_ISL_6971472, EPI_ISL_7606033, EPI_ISL_7632035, EPI_ISL_7632045	Stadtspital Triemli	Institute of Medical Virology, University of Zurich	Alexandra Trkola; Annette Audigé; Catharine Aquino; Cyril Shah; Daniel Ehrsam; Gabriela Ziltener; Guido Bloemberg; Hubert Rehrauer; Isabel Stürmer; Joel Wirz; Jon Huder; Jürg Böni; Kevin Steiner; Maria Grünberg; Maryam Zaheri; Michael Huber; Riccarda Capaul; Stefan Schmutz; Verena Kufner; Weihong Qi
EPI_ISL_7650507	StarMed Healthcare	UNC Charlotte Environmental Monitoring Laboratory	Cynthia Gibas; Jannatul Ferdous; Jessica Schlueter; Juan Bolanos; Kevin Lambirth; Samuel Kunkleman; Torri Weathers
EPI_ISL_7445855	State Hygienic Laboratory at the University of Iowa	State Hygienic Laboratory at the University of Iowa	Alankar Kampoowale; Anna Yakos; Cindy Toll; Davis Rieckenberg; Erik Twait; Jeff Benfer; Kris Eveland; Krishnaveni Sompallae; Kristen Zanon; Mariah Knutson; Mohammed Allam; Valerie Reeb; Wes Hottel
EPI_ISL_7013425, EPI_ISL_7264087, EPI_ISL_7264088, EPI_ISL_7456351, EPI_ISL_7456393, EPI_ISL_7456394, EPI_ISL_7456395, EPI_ISL_7456396, EPI_ISL_7456397, EPI_ISL_7456398, EPI_ISL_7456399, EPI_ISL_7456400, EPI_ISL_7620798, EPI_ISL_7620819			
see above	State Laboratories Division, Hawaii State Department of Health	State Laboratories Division, Hawaii State Department of Health	Ayana Garnet; Daniel Strange; Drew Kuwazaki; Edward Desmond; Pamela O'Brien; Razvan Sultana
EPI_ISL_7544013, EPI_ISL_7544494, EPI_ISL_7544584, EPI_ISL_7544730	Study: Rapid Diag POC Covid	NHLS/UCT	Arash Iranzadeh; Bruna Galvao; Carolyn Williamson; Deelan Doolabh; Diana Hardie; Gert Marais; Innocent Mudau; Luicer Olubayo; Lynn Tyers; Marvin Hsiao; Nokuzola Mbhele; Rageema Joseph; Stephen Korsman
EPI_ISL_7364700, EPI_ISL_7364701, EPI_ISL_7364702	Summit Clinical Laboratories	City of Milwaukee Health Department Laboratory	Amy Bauer; Manjeet Khubbar; Samantha Scott; Sanjib Bhattacharyya
EPI_ISL_6883250, EPI_ISL_7452246, EPI_ISL_7452247	Swedish national genomic surveillance program of SARS-CoV-2	The Public Health Agency of Sweden	Alma Brolund; Maria Lind Karlberg; Maximilian Riess; Swedish national genomic surveillance program of SARS-CoV-2
EPI_ISL_7457427, EPI_ISL_7457428, EPI_ISL_7457429, EPI_ISL_7457430, EPI_ISL_7457431	Synlab Medilab	Karolinska University Hospital Huddinge	Annika Tiveljung Lindell; Henning Onsbring; Jan Albert; Karina Hentrich; Lynda Eneh; Martin Ekman; Natalija Gerasimcik; Robert Dyrdak; Sandra Broddesson; Shambhu Ganeshappa Aralaguppe; Tanja Normark; Tobias Allander; Valtteri Wirta; Zhibing Yun
EPI_ISL_7605688	TINTSWALO LABORATORY	National Institute for Communicable Diseases of the National Health Laboratory Service	Amoako DG; Bhiman JN; Everatt J; Ismail A; Mahlangu B; Mnguni A; Mohale T; Ntuli N; Scheepers C; Wolter N
EPI_ISL_7605602, EPI_ISL_7605603, EPI_ISL_7605604, EPI_ISL_7605605, EPI_ISL_7605606, EPI_ISL_7605608, EPI_ISL_7605609, EPI_ISL_7605613, EPI_ISL_7605614, EPI_ISL_7605615, EPI_ISL_7605616, EPI_ISL_7605620, EPI_ISL_7605621, EPI_ISL_7605623, EPI_ISL_7605665, EPI_ISL_7605684, EPI_ISL_7605746, EPI_ISL_7605747, EPI_ISL_7605766			
see above	TSHEPONG LABORATORY	National Institute for Communicable Diseases of the National Health Laboratory Service	Amoako DG; Bhiman JN; Everatt J; Ismail A; Mahlangu B; Mnguni A; Mohale T; Ntuli N; Scheepers C; Wolter N
EPI_ISL_7605805	TXDSHS	TXDSHS	Anita Pokharel; Bonnie Oh; Chun Wang; Grace Kubin; Karen Bobier; Maliha Rahman; Mayela Pedrueza; Rachel Lee; Rashmi Tuladhar
EPI_ISL_7456440	Tambo Memorial Laboratory	National Institute for Communicable Diseases of the National Health Laboratory Service	Amoako DG; Bhiman JN; Everatt J; Ismail A; Mahlangu B; Mnguni A; Mohale T; Ntuli N; Scheepers C; Wolter N
EPI_ISL_7571540	Temporary Specimen Collection Centre at the AsiaWorld-Expo	Hong Kong Department of Health	Alan K.L. Tsang; Edman T.K. Lam; Ken H.L. Ng; Patricia K. L. Leung; Peter C.W. Yip; Rickjason C.W. Chan
EPI_ISL_6825551	Territory Pathology	Territory Pathology	Dimitrios Menouhos; Ella Meumann; Robert Baird
EPI_ISL_7171744	The Hope Clinic of Emory Vaccine Center, Emory University	Piantadosi Lab, Emory Department of Pathology	Anne Piantadosi; Azmain Taz; Dara Khosravi; Ethan Wang; Jesse Waggoner; Ludy Carmola; Marybeth Sexton; Nadine Rouphael
EPI_ISL_7446810	Thüringer Landesamtes für Verbraucherschutz	Robert Koch Institute	
EPI_ISL_7647061	Trinidad Public Health Laboratory	Carrington Lab, Department of Preclinical Sciences, Faculty of Medical Sciences, The University of the West Indies, St Augustine Campus	Anushka Ramjag; Arianne Brown-Jordan; Avery Hinds; Christine V. F. Carrington; Christopher Oura; Gabriel Escobar; Nikita S. D. Sahadeo; Nuno Faria; Oliver Pybus; Risha Singh; Roshan Parasram; Sarah Hill; Soren Nicholls; SueMin Nathaniel; Vernie Ramkissoon
EPI_ISL_7235629, EPI_ISL_7660974, EPI_ISL_7660975, EPI_ISL_7660976, EPI_ISL_7660977, EPI_ISL_7660978, EPI_ISL_7660979, EPI_ISL_7660980, EPI_ISL_7660981, EPI_ISL_7660982, EPI_ISL_7660983, EPI_ISL_7660984, EPI_ISL_7660985, EPI_ISL_7660986, EPI_ISL_7660987, EPI_ISL_7660989, EPI_ISL_7660991, EPI_ISL_7660993, EPI_ISL_7660994, EPI_ISL_7660995, EPI_ISL_7660996			

see above	Tulane University School of Medicine	Tulane University School of Medicine	Di Tian
EPI_ISL_7505962	UC Davis Genome Center	UC Davis Genome Center	Healthy Davis Together; UC Davis
EPI_ISL_7649984, EPI_ISL_7649985, EPI_ISL_7649986, EPI_ISL_7649987, EPI_ISL_7649988, EPI_ISL_7649989, EPI_ISL_7649990, EPI_ISL_7649991, EPI_ISL_7649992, EPI_ISL_7649993, EPI_ISL_7649994, EPI_ISL_7649995, EPI_ISL_7649996, EPI_ISL_7649997, EPI_ISL_7649998, EPI_ISL_7649999, EPI_ISL_7650000			
see above	UNAM Molecular Diagnostic Laboratory	Forschungszentrum Borstel	Azaria Diergaardt; Christian Utpatel; Emmanuel Nepolo; Ivan Barilar; Jasmin Scharnberg; Loide Shipingana; Lusia Mhuulu; Stefan Niemann; Tanja Niemann; Vanessa Mohr
EPI_ISL_7451261	UNILABS	Instituto Nacional de Saude (INSA)	Borges et al
EPI_ISL_7160037, EPI_ISL_7160038, EPI_ISL_7160039, EPI_ISL_7545421, EPI_ISL_7545422, EPI_ISL_7545423, EPI_ISL_7545424			
see above	UW Virology Lab	UW Virology Lab	Alexander Greninger; Hong Xie; Isabel Arnould; Keith R Jerome; Meei-Li Huang; Nathan Breit; Patrick Mathias; Pavitra Roychoudhury; Pooneh Hajian; Ricardo Perez; Robert J. Livingston; Saraswathi Sathees; Sean Ellis; Seffir T. Wendm; Shah Mohamed Bakhash; Tien V. Nguyen
EPI_ISL_7544861, EPI_ISL_7631788	Unilabs	Institute of Medical Virology, University of Zurich	Alexandra Trkola; Annette Audigé; Catharine Aquino; Cyril Shah; Daniel Ehrsam; Gabriela Ziltener; Guido Bloemberg; Hubert Rehrauer; Isabel Stürmer; Joel Wirz; Jon Huder; Jürg Böni; Kevin Steiner; Maria Grünberg; Maryam Zaheri; Michael Huber; Riccarda Capaul; Stefan Schmutz; Verena Kufner; Weihong Qi
EPI_ISL_7470330, EPI_ISL_7470341	Unilabs Laboratory Medicine	Norwegian Institute of Public Health, Department of Virology	Atiya R Ali; Debech Nadia; Engebretsen Serina Beate; Garcia Llorente Ignacio; Hilde Elshaug; Hilde Vollan; Jon Bråte; Kamilla Heddeland Instefjord; Karoline Bragstad; Kathrine Stene-Johansen; Line Victoria Moen; Marie Paulsen Madsen; Olav Hungnes; Pedersen Benedikte Nevjen; Rasmus Riis Kopperud
EPI_ISL_7141056	Unipath Speciality Laboratory Limited, Ahmedabad	Gujarat Biotechnology Research Centre	Apurvasinh Puvar; Bhadreshsinh Gohil; Chaitanya Joshi; Dinesh Kumar; Janvi Raval; Jwalant Shah; Madhvi Joshi; Nimesh Patel; Nitin Savaliya; Nitin Shukla; Priyank Chavda; Ramesh Pandit; Sonal Sharma; Zarna Patel
EPI_ISL_7613414	University Hospital, San Antonio	STRL UT Health San Antonio, Greehey Children's Cancer Research Institute	Bethany Landry; Dawn Garcia; Guillermo Nunez; Hongxin Fan; Josefina Stoever; Korri Weldon; Kumari Vadlamudi; Marjorie Parker David; San Antonio Metropolitan Health District; Texas Department of State Health Services; Weijing He; Yidong Chen; Zhao Lai; Zhenqing Ye
EPI_ISL_7605542, EPI_ISL_7605543, EPI_ISL_7605546, EPI_ISL_7605547	University Hospitals of Geneva, Laboratory of Virology	HUG, Laboratory of Virology and the Health2030 Genome Center	Aline Mamin; Ana Rita Goncalves; Cedric Howald; Deborah Penet; Francisco Perez; Henri Pegeot; Ioannis Xenarios; Keith Harshman; Laurent Kaiser; Lorenzo Cerutti; Melyssa Elies; Samuel Cordey
EPI_ISL_7542255	University Medical Center Hamburg Eppendorf	Heinrich Pette Institute, Leibniz Institute for Experimental Virology	Adam Grundhoff; Alexis Robitaille; Johannes Knobloch; Martin Aepfelbacher; Nicole Fischer; Thomas Günther
EPI_ISL_6929785, EPI_ISL_7195724, EPI_ISL_7195725, EPI_ISL_7195726, EPI_ISL_7405404, EPI_ISL_7405405, EPI_ISL_7405406, EPI_ISL_7405407, EPI_ISL_7405408, EPI_ISL_7405409, EPI_ISL_7405412, EPI_ISL_7462253, EPI_ISL_7462258, EPI_ISL_7462265, EPI_ISL_7462271, EPI_ISL_7601672			
see above	University of Liège COVID-19 testing center	GIGA Medical Genomics	Bouchra Boujemla; Claire Gourzonès; Cécile Meex; Keith Durkin; Laurent Gillet; Maria Artesi; Marie-Pierre Hayette; Nadine Cambisano; Nathalie Renotte; Olivier Ek; Sébastien Bontems; Vincent Bours
EPI_ISL_7651793	University of New Mexico Hospital	Center for Global Health, University of New Mexico Health Sciences Center	Darrell Dinwiddie; Daryl Domman; Jesse Young; Jon Femling; Kurt Schwalm; Valery Morley
EPI_ISL_7379462	University of Wisconsin-Madison AIDS Vaccine Research Laboratories	University of Wisconsin-Madison AIDS Vaccine Research Laboratories	Gage Moreno; Katarina Braun; et al. AIDS Vaccine Research Laboratories
EPI_ISL_7605921, EPI_ISL_7605979, EPI_ISL_7606032	Universität Zürich	Institute of Medical Virology, University of Zurich	Alexandra Trkola; Annette Audigé; Catharine Aquino; Cyril Shah; Daniel Ehrsam; Gabriela Ziltener; Guido Bloemberg; Hubert Rehrauer; Isabel Stürmer; Joel Wirz; Jon Huder; Jürg Böni; Kevin Steiner; Maria Grünberg; Maryam Zaheri; Michael Huber; Riccarda Capaul; Stefan Schmutz; Verena Kufner; Weihong Qi
EPI_ISL_7544862, EPI_ISL_7544872, EPI_ISL_7631828	UniversitätsSpital Zürich	Institute of Medical Virology, University of Zurich	Alexandra Trkola; Annette Audigé; Catharine Aquino; Cyril Shah; Daniel Ehrsam; Gabriela Ziltener; Guido Bloemberg; Hubert Rehrauer; Isabel Stürmer; Joel Wirz; Jon Huder; Jürg Böni; Kevin Steiner; Maria Grünberg; Maryam Zaheri; Michael Huber; Riccarda Capaul; Stefan Schmutz; Verena Kufner; Weihong Qi
EPI_ISL_7418452, EPI_ISL_7418453, EPI_ISL_7418454, EPI_ISL_7418455, EPI_ISL_7418456, EPI_ISL_7418463, EPI_ISL_7418464, EPI_ISL_7418465			
see above	Universitätsklinikum Frankfurt - Institut für Medizinische Virologie	Robert Koch Institute	
EPI_ISL_7336152	Utah Public Health Laboratory	Utah Public Health Laboratory	Erin L. Young; John Arnn; Kelly F. Oakeson; Olinto Linares-Perdomo; Pooja Gupta; Tom Iverson
EPI_ISL_7261603	VA Tampa Healthcare System	VHA Public Health Reference Laboratory	Mark Holodniy on behalf of VA SEQFORCE; US Department of Veterans Affairs
EPI_ISL_6989662	Vault Health	Minnesota Department of Health, Public Health Laboratory	Alyssa Mondelli; Elizabeth Horn; Jacob Garfin; Kelly Pung; Matt Plumb; Sarah Namugenyi; and Xiong Wang
EPI_ISL_7398681, EPI_ISL_7398758	Vichaivej International Hospital Nongkhaem	National Institute of Health, Department of Medical Sciences, Ministry of Public Health, Thailand	Archawin Rojanawiwat; Ballang Uppapong; Beth Skaggs; Donlaya Maunplueng; Kazuhisa Okada; Natchaya Khiadsang; Nuttida Thongpramul; Pakorn Piromtong; Pilailuk Akkapaiboon Okada; Piroon Jenjaroenpun; Pongpun Sawatwong; Prapat Suriyaphol; Sirikanda Wimol; Siripaporn Phuygun; Sittiporn Parnmen; Supakit Sirilak; Suratchana Mitrat; Thanutsapa Thanadachakul; Thidathip Wongsurawat
EPI_ISL_7224567, EPI_ISL_7224577	Viollier AG	Clinical Bacteriology, University Hospital Basel	Adrian Egli; Alfredo Mari; Christiane Beckmann; Fanny Wegner; Hans Hirsch; Helena MB Seth-Smith; Julia Bielicki; Karoline Leuzinger; Manuel Battegay; Tim Roloff
EPI_ISL_7371749, EPI_ISL_7371751, EPI_ISL_7372207, EPI_ISL_7566519, EPI_ISL_7566755, EPI_ISL_7566802, EPI_ISL_7566826, EPI_ISL_7566875, EPI_ISL_7566947, EPI_ISL_7620490, EPI_ISL_7620551, EPI_ISL_7652802, EPI_ISL_7652817, EPI_ISL_7652873, EPI_ISL_7652883, EPI_ISL_7652959, EPI_ISL_7652962, EPI_ISL_7652983, EPI_ISL_7653300, EPI_ISL_7653392, EPI_ISL_7653449, EPI_ISL_7653461			
see above	Viollier AG	Department of Biosystems Science and Engineering, ETH Zürich	Andrea Patrignani; Andrea Patrizia Salzmann; Andreia Cabral de Gouvea; Catharine Aquino; Catharine Aquino Fournier; Chaoran Chen; Christian Beisel; Christian Urban; Christiane Beckmann; Christoph Noppen; Daniel Ehrsam; David Dreifuss; Doris Popovic; Elodie Burcklen; Franziska Singer; Griffin White; Hai Bui; Henriette Kurth; Ina Nissen; Isabel Stürmer; Ivan Topolsky; Jay Tracy; Kim Philipp Jablonski; Lara Fuhrmann; Laura Neff; Lennart Opitz; Louis du Plessis; Maria Domenica Moccia; Matteo Carrara; Maurice Redondo; Mirjam Feldkamp; Natascha Santacroce; Niko Beerenwinkel; Olivier Kobel; Pelin Icer; Ralph Schlapbach; Rebecca Denes; Sarah Nadeau; Shuqing Yu; Simon Grüter; Tanja Stadler; Timothy Sykes; Tobias Schär
EPI_ISL_7509012, EPI_ISL_7509019, EPI_ISL_7509025, EPI_ISL_7509030, EPI_ISL_7561327, EPI_ISL_7561395, EPI_ISL_7561411, EPI_ISL_7561414, EPI_ISL_7561423, EPI_ISL_7561425, EPI_ISL_7561434, EPI_ISL_7561441, EPI_ISL_7561446, EPI_ISL_7561454, EPI_ISL_7561464, EPI_ISL_7561472, EPI_ISL_7561477, EPI_ISL_7561482			
see above	Virology Department, Royal Infirmary of Edinburgh, NHS Lothian / School of Biological Sciences, University of Edinburgh	COVID-19 Genomics UK (COG-UK) Consortium	Colquhoun R; Cotton S; Dewar R; Fernandez G; Gallagher A; Hill V; Jackson B; Maloney D; McCrone JT; McHugh M; O'Toole Á; Rambaut A; Scher E; Templeton K; Yu X
EPI_ISL_7285844, EPI_ISL_7285845, EPI_ISL_7285846, EPI_ISL_7285847, EPI_ISL_7285848	Virology Laboratory, Scientific Department, Army Medical Center	Virology Laboratory, Scientific Department, Army Medical Center	Anella Monte; Anna Anselmo; Antonella Fortunato; Filippo Molinari; Florigio Lista; Francesco Giordani; Giancarlo Petralito; Giandomenico Cerreto; Giulia Campoli; Lucia Nicosia; Marzia Cavalli; Pietro Marco D'Angelo; Riccardo De Sanctis; Rossella Brandi; Silvia Fillo; Vanessa Vera Fain
EPI_ISL_7503508, EPI_ISL_7503509	Washington State Department of Health Public Health Laboratories	Washington State Department of Health Public Health Laboratories	Alex Lathum; Ardizon Valdez; Avi Singh; Claire Howell; Denny Russell; Drew MacKellar; Holly Halstead; JohnAric Peterson; Kathryn Sickles; Kristin Roche; Lisa Jones; Philip Dykema; Rebecca Cao
EPI_ISL_7548944, EPI_ISL_7548945	Wexner Medical Center	OSU College of Medicine	Corcoran, S.; Koenig, S.
EPI_ISL_7662792, EPI_ISL_7662814, EPI_ISL_7662820	Willis-Knighton Medical Center Hospital Laboratory	LSUHS Emerging Viral Threat Laboratory	Adrian Almodovar; Alexander Mijalis; Andrew D. Yurochko; April N. Johnson; Christopher G. Kevil; Gregory L. Ware; Jennifer L. Carroll; Jeremy P. Kamil; John A. Vanchiere; Joseph A. Bocchini; Krista Queen; Lorie M. Atkins; Maarten Van Diest; Rona S. Scott
EPI_ISL_7501188	Winchester Hospital via Lahey Hospital	New England Biolabs	Abel, G.; B.W.; C.J.; Colgrove, R.; Duncan, R.; Elfahal, M.; Flynn; Heim, K.; Karolides, M.; L. and Langhorst; Michaels, L.; Pinet, K.; Skelton, T.; Sun
EPI_ISL_7263803	Wisconsin State Laboratory of Hygiene Communicable Disease Division	Wisconsin State Laboratory of Hygiene Communicable Disease Division	Abigail C. Shockey; Alicia J. Mooney; Erika M. Hanson; Kelsey R. Florek; Richard Griesser; Sara Wagner; Tonya Danz
EPI_ISL_7478524, EPI_ISL_7478525, EPI_ISL_7478526, EPI_ISL_7478527, EPI_ISL_7478529, EPI_ISL_7478530, EPI_ISL_7502154, EPI_ISL_7502155			
see above	Yale Clinical Virology Lab	Grubaugh Lab - Yale School of Public Health	Anderson Brito; Chaney Kalinich; Chantal Vogels; Isabel Ott; Joseph Fauver; Kendall Billig; Mallery Breban; Marie L. Landry; Mary Petrone; Nathan Grubaugh; Tobias Koch
EPI_ISL_7472293, EPI_ISL_7661000, EPI_ISL_7661001, EPI_ISL_7661002	Yale Pathology Labs	Yale Pathology Labs	Angelique Levi; Brian Daley; Chen Liu; Guangxiao Yang; Heidi Herrick; Jianhui Wang; Jinglan Wang; John Sinard; Katherine Fajardo; Kevin Schofield; Laura Brady; Michael Stankewich; Minghao Zhong; Monica Talmor; Pei Hui; Peter Gershkovich; Richard Bouffard; Stephanie Weirsman; Susan Bell; Sylvia White
EPI_ISL_6795833, EPI_ISL_6795835, EPI_ISL_6795836, EPI_ISL_6795837, EPI_ISL_6795838, EPI_ISL_6795839, EPI_ISL_6795840, EPI_ISL_6795841, EPI_ISL_6795842, EPI_ISL_6795844, EPI_ISL_6795845, EPI_ISL_6795846, EPI_ISL_6795847, EPI_ISL_6795848, EPI_ISL_6795849, EPI_ISL_6795850, EPI_ISL_6825389, EPI_ISL_6825390, EPI_ISL_6825391, EPI_ISL_6825392, EPI_ISL_6825393, EPI_ISL_6825394, EPI_ISL_6825395, EPI_ISL_6825396, EPI_ISL_6825397, EPI_ISL_6825398, EPI_ISL_7015172, EPI_ISL_7015173, EPI_ISL_7015174, EPI_ISL_7015175, EPI_ISL_7015176, EPI_ISL_7015177, EPI_ISL_7015178, EPI_ISL_7015179, EPI_ISL_7015180, EPI_ISL_7015181, EPI_ISL_7015182, EPI_ISL_7015183, EPI_ISL_7015184, EPI_ISL_7015185, EPI_ISL_7015186, EPI_ISL_7015187, EPI_ISL_7015188, EPI_ISL_7015189, EPI_ISL_7015190, EPI_ISL_7015191, EPI_ISL_7015192, EPI_ISL_7015193, EPI_ISL_7015194, EPI_ISL_7015195, EPI_ISL_7015196, EPI_ISL_7015197, EPI_ISL_7015198, EPI_ISL_7015199, EPI_ISL_7015200, EPI_ISL_7015201, EPI_ISL_7015202, EPI_ISL_7015203, EPI_ISL_7015204, EPI_ISL_7015205, EPI_ISL_7015206, EPI_ISL_7015207, EPI_ISL_7015208			
see above	ZARV/NHLS, Department Medical Virology, University of Pretoria	CERI, Centre for Epidemic Response and Innvoation, Stellenbosch University and KRISP, KZN Research Innovation and Sequencing Platform, UKZN.	Adriano Mendes; Amoaka D; Amy Strydom; Arisha Maharaj; Bester P; Bhiman J; Engelbrecht S; Everatt J; Giandhari J; Goedhals D; Hardie D; Hsiao M; Iranzadeh A; Lessells R; Makatini Z; Maponga T; Mdlalose N; Micheala Davids; Mlisana K; Moir M; NGS-SA (Scheepers C; Naidoo Y; Nyaga M) Giandhari J; Oluwakemi M; Pillay S; Preiser W; Ramphal U; Ramphal Y; San JE; Sim Mayaphi and Marietjie Venter; Tegally H; Tshiabuila D; Venter M; Wilkinson E; Williamson C; de Oliveira T; von Gottberg A
EPI_ISL_7548959, EPI_ISL_7548966, EPI_ISL_7549083	ZOTZ KLIMAS MVZ Düsseldorf-Centrum GbR ÜBAG für Labormedizin, Genetik, Zytologie,	Center of Medical Microbiology, Virology, and Hospital Hygiene, University of Duesseldorf	Alexander Dilthey; Andreas Walker; Daniel Strelow; Jessica Nicolai; Jörg Timm; Katrin Hoffmann; Klaus Pfeffer; Lisanna Hülse; Malte Kohns Vasconcelos; Maximilian Damagnez; Nadine Lübke; Patrick Finzer; Rainer Zotz; Tobias Wienemann; Torsten Houwaart

	Pathologie		
EPI_ISL_7544865, EPI_ISL_7606038	Zentrallabor Zürich	Institute of Medical Virology, University of Zurich	Alexandra Trkola; Annette Audigé; Catharine Aquino; Cyril Shah; Daniel Ehrsam; Gabriela Ziltener; Guido Bloemberg; Hubert Rehrauer; Isabel Stürmer; Joel Wirz; Jon Huder; Jürg Böni; Kevin Steiner; Maria Grünberg; Maryam Zaheri; Michael Huber; Riccarda Capaul; Stefan Schmutz; Verena Kufner; Weihong Qi
EPI_ISL_7423603	amedes MVZ für Laboratoriumsdiagnostik Raubling GmbH	Robert Koch Institute	
EPI_ISL_7368223, EPI_ISL_7594806	labor team w AG	Department of Biosystems Science and Engineering, ETH Zürich	Andrea Patrignani; Andreas Lindauer; Andreia Cabral de Gouvea; Catharine Aquino; Catharine Aquino Fournier; Chaoran Chen; Christian Urban; Daniel Ehrsam; David Dreifuss; Doris Popovic; Franziska Singer; Griffin White; Hai Bui; Isabel Stürmer; Ivan Topolsky; Jay Tracy; Kim Philipp Jablonski; Lara Fuhrmann; Laura Neff; Lennart Opitz; Louis du Plessis; Maria Domenica Moccia; Matteo Carrara; Monika Bucher; Niko Beerenwinkel; Pelin Icer; Ralph Schlapbach; Rebekka Pohl; Sarah Nadeau; Shuqing Yu; Simon Grüter; Tanja Stadler; Timothy Sykes
