# Supplementary material for: An expanded RT-PCR melting temperature coding assay to rapidly identify all known SARS-CoV-2 variants and sub-variants of concern
Source: Sci Rep. 2023 Dec 11;13:21927. doi: 10.1038/s41598-023-48647-8 (PMC10713575; doi:10.1038/s41598-023-48647-8)
Supplement: Supplementary file 3 — Supplementary Information 3. [file 41598_2023_48647_MOESM3_ESM.docx]

***Supplementary materials***

An expanded RT-PCR melting temperature coding assay to rapidly identify all known SARS-CoV-2 variants and sub-variants of concern.

Padmapriya P Banada^a^, Raquel Green^a^, Deanna Streck^b^, Robert Reiss^d^, Sukalyani Banik^a^, Ibsen Montalvan^c^, Robert Jones^e^, Salvatore A. E. Marras^a^, Soumitesh Chakravorty^a,f^ and David Alland^a^

^a^Public Health Research Institute, Rutgers New Jersey Medical School, Newark, NJ; ^b^Institute of Genomic Medicine, Rutgers New Jersey Medical School, Newark, NJ; ^c^University Hospital, Newark, NJ;
^d^Division of Infectious Diseases, Department of Medicine, Rutgers New Jersey Medical School, Newark, NJ;
 ^e^Craic Computing LLC, Snohomish, WA; ^f^Cepheid, Sunnyvale, CA.

Supplementary Table 1S. Primers, probes, and PCR conditions.

| Assay | Primer/ Probe | Sequence 5’ -> 3’ | Amplicon size | Final conc. per reaction | Thermal cycling and post PCR melt conditions |
| --- | --- | --- | --- | --- | --- |
| **SMB-501** | 501-F | ggttttaattgttactttcctttacaa | 89 bp | 0.2 µM | Uracil DNA glucosylase incubation: 37°C-2 min;  Reverse transcription (RT):  50°C-15 min;  Asymmetric PCR:  Denaturation: 95 °C- 3s;  Annealing/extension: 58°C-30s.  Cycles: 45  Post-PCR melt:  Denaturation: 95°C- 30s;  Melt: 45°C to 85°C;  Acquisitions:   Continuous at 2/°C |
|  | 501-R | gaaagtactactactctgtatggttgg |  | 2.0 µM |  |
|  | 501-WT | Q570-CCGCgtt[pdU]ccatcccactaatgctg[pdU]tggttaccaacGCGG-BHQ2 |  | 0.4 µM |  |
|  | 501- MT | Q670-CGCGgtt[pdu]ccatcccacttatgctg[pdu]tggttaccaacCGCG-BHQ2 |  | 0.4 µM |  |
| **SMB-484** | 484-Fw* | ctatcaggccggtaRcaMac | 76 bp | 0.2 µM |  |
|  | 484-Rw* | gaaacYatatgatYgtaaaggaaag |  | 5.0 µM |  |
|  | 484-WT | Q570-CCGCGccttgtaatggtgttgaaggttttaattgttacGCGCGG-BHQ2 |  | 0.4 µM |  |
|  | 484-MT | Q670-CCGCGccttgtaatggtgttaaaggttttaattgttacGCGCGG-BHQ2 |  | 0.4 µM |  |
| **SMB-452** | 452-F | aggctgcgttatagcttgga | 122 bp | 0.1 µM |  |
|  | 452-R | cagttgaaatatctctctcaaaaggt |  | 2.5 µM |  |
|  | 452-WT | Q570-CCGCGtaattataattacctgtatagattgtttaggaagtcCGCGG-BHQ2 |  | 0.4 µM |  |
|  | 452-MT | Q670-CGCCGAggtggtaattataattaccggtatagattgtttaggTCGGCG-BHQ2 |  | 0.4 µM |  |

The lowercase letters indicate the SMB probe region; the uppercase letters indicate the SMB stem region; pdU, C5 Propynyl-deoxyuridine; Q570, Quasar 570;
Q670, Quasar 670; and BHQ indicates “Black Hole Quencher”. *Wobble primers: R=A+G; M=A+C; Y=C+T.

Supplementary Table 2S. Patient specimen validation in four different RT-PCR instruments with melt curve analysis using 3 SMB assays for detection and differentiation of SARS-Cov-2 variants of concern.

| **Ref. /Pt. samples** | **DOC** | **Source** | **IC Ct** | **LC480** | | | | | | | **CFX96** | | | | | | | | | | **RGQ** | | | | | | | | | **ABI 7500** | | | | | | | | | | |
| --- | --- | --- | --- | --- | --- | --- | --- | --- | --- | --- | --- | --- | --- | --- | --- | --- | --- | --- | --- | --- | --- | --- | --- | --- | --- | --- | --- | --- | --- | --- | --- | --- | --- | --- | --- | --- | --- | --- | --- | --- |
|  |  |  |  | **SMB-501 Assay** | | **SMB-484 Assay** | | **SMB-452 Assay** | | **Final id** | **SMB-501 Assay** | | **SMB-484 Assay** | | **SMB-452 Assay** | | | | **Final id** | | **SMB-501 Assay** | | **SMB-484 Assay** | | | **SMB-452 Assay** | | **Final id** | | **SMB-501 Assay** | | | **SMB-484 Assay** | | | **SMB-452 Assay** | | | **Final id** | |
|  |  |  |  | **WT (Cy3)** | **MT (Cy5)** | **WT (Cy3)** | **MT (Cy5)** | **WT (Cy3)** | **MT (Cy5)** |  | **WT (Cy3)** | **MT (Cy5)** | **WT (Cy3)** | **MT (Cy5)** | **WT (Cy3)** | **MT (Cy5)** | |  | | **WT (Cy3)** | | **MT (Cy5)** | **WT (Cy3)** | **MT (Cy5)** | | **WT (Cy3)** | **MT (Cy5)** |  |  | **WT (Cy3)** | | **MT (Cy5)** | **WT (Cy3)** | | **MT (Cy5)** | **WT (Cy3)** | | **MT (Cy5)** |  | |
| WT-Reference | | BEI NR52285 |  | 59.7 | 58.9 | 65.7 | 60.6 | 61.9 | 59.9 |  | 59.0 | 58.0 | 65.0 | 59.8 | 62.0 | | 60.0 |  | | 61.1 | | 59.3 | 66.6 | | 60.7 | 64.1 | 59.5 |  | 59.4 | | 57.9 | 64.9 | | 59.8 | 62.1 | | 58.9 |  | |  |
| MT-B.1.1.7 (Alpha) | | BEI NR54000 |  | 55.7 | 62.6 | 65.7 | 60.6 | 61.9 | 59.9 |  | 54.5 | 62.0 | 65.0 | 59.8 | 62.0 | | 60.0 |  | | 55.6 | | 63.2 | 66.6 | | 60.7 | 64.1 | 59.5 |  | 53.3 | | 62.1 | 64.9 | | 59.8 | 62.1 | | 58.9 |  | |  |
| MT-B.1.351 (Beta) | | BEI NR-55282 |  | 55.7 | 62.6 | 62.2 | 64.7 | 61.9 | 59.9 |  | 54.5 | 62.0 | 61.0 | 64.0 | 62.0 | | 60.0 |  | | 55.6 | | 63.2 | 62.9 | | 65.2 | 64.1 | 59.5 |  | 53.3 | | 62.1 | 61.3 | | 64.2 | 62.1 | | 58.9 |  | |  |
| MT-B.1.617.2 (Delta) | | BEI NR-55611 |  | 59.7 | 58.9 | 65.7 | 60.6 | 58.3 | 63.3 |  | 59.0 | 58.0 | 65.0 | 59.8 | 58.0 | | 64.0 |  | | 61.1 | | 59.3 | 66.6 | | 60.7 | 0 | 64.5 |  | 59.4 | | 57.9 | 64.9 | | 59.8 | 57.5 | | 62.9 |  | |  |
| MT-B.1.1.529 (Omicron) | | BEI NR-56461 |  | 49.0 | 56.6 | 63.4 | 60.6 | 61.9 | 59.9 |  |  |  |  |  |  |  | |  | |  | |  |  |  | |  |  |  |  | |  |  | |  |  | |  |  | |  |
| VSAP1 | Apr 2021 | NP | 27.7 | 59.6 | 58.7 | 65.5 | 60.4 | 62.7 | 60.4 | ***WT*** | 59.0 | 58.0 | 65.0 | 60.0 | 62.0 | 59.0 | | ***WT*** | | 61.5 | | 0 | 66.7 | 61.0 | | 63.8 | 60.5 | ***WT*** | 59.7 | | 58.2 | 65.2 | | 59.9 | 62.4 | | 59.6 | ***WT*** | |  |
| VSAP2 | Apr 2021 | NP | 29.3 | 55.1 | 62.2 | 65.3 | 60.1 | 62.4 | 60.1 | ***Alpha*** | 54.0 | 62.0 | 65.0 | 59.0 | 62.0 | 0.0 | | ***P-Alpha*** | | 0 | | 0 | 66.8 | 61.0 | | 64.0 | 60.8 | ***Ind*** | 53.2 | | 61.9 | 64.8 | | 59.5 | 62.1 | | 59.1 | ***Alpha*** | |  |
| VSAP3 | May 2021 | NP | 29.2 | 60.4 | 59.1 | 65.9 | 60.6 | 59.2 | 64.2 | ***Delta*** | 59.0 | 57.0 | 65.0 | 59.0 | 58.0 | 64.0 | | ***Delta*** | | 0 | | 0 | 67.0 | 61.0 | | 0 | 0 | ***Ind*** | 59.5 | | 57.1 | 65.4 | | 59.7 | 57.7 | | 63.6 | ***Delta*** | |  |
| VSAP4 | May 2021 | NP | 25.6 | 55.1 | 62.5 | 65.6 | 60.3 | 62.6 | 59.9 | ***Alpha*** | 54.0 | 61.0 | 65.0 | 59.0 | 62.0 | 0.0 | | ***P-Alpha*** | | 0 | | 0 | 66.8 | 61.0 | | 64.3 | 0 | ***Ind*** | 53.6 | | 62.1 | 65.2 | | 59.7 | 62.3 | | 59.1 | ***Alpha*** | |  |
| VSAP5 | May 2021 | Saliva | 23.7 | 60.4 | 58.5 | 65.3 | 60.2 | 58.5 | 63.5 | ***Delta*** | 59.0 | 58.0 | 65.0 | 60.0 | 58.0 | 64.0 | | ***Delta*** | | 61.3 | | 59.3 | 66.7 | 61.0 | | 59.8 | 64.7 | ***Delta*** | 59.5 | | 58.0 | 65.2 | | 59.7 | 58.0 | | 63.5 | ***Delta*** | |  |
| VSAP6 | May 2021 | Saliva | 26.9 | 56.1 | 63.5 | 66.9 | 61.5 | 62.8 | 60.6 | ***Alpha*** | 55.0 | 62.0 | 65.0 | 60.0 | 63.0 | 59.0 | | ***Alpha*** | | 56.0 | | 63.3 | 67.0 | 61.2 | | 64.5 | 0 | ***Alpha*** | 53.7 | | 62.3 | 65.4 | | 59.9 | 62.6 | | 0 | ***P-Alpha*** | |  |
| VSAP7 | June 2021 | NP | 29.6 | 60.6 | 59.8 | 66.6 | 61.3 | 60.1 | 64.8 | ***Delta*** | 60.0 | 59.0 | 65.0 | 61.0 | 59.0 | 65.0 | | ***Delta*** | | 0 | | 0 | 66.8 | 61.0 | | 59.7 | 64.7 | ***Ind*** | 59.1 | | 58.6 | 65.0 | | 59.6 | 58.0 | | 63.4 | ***Delta*** | |  |
| VSAP8 | July 2021 | NP | 29.7 | 60.0 | 59.1 | 65.8 | 60.5 | 59.2 | 64.0 | ***Delta*** | 59.0 | 58.0 | 65.0 | 59.0 | 0 | 64.0 | | ***P-Delta*** | | 61.5 | | 60.0 | 67.5 | 61.5 | | 0 | 65.5 | ***Delta*** | 59.5 | | 57.6 | 65.1 | | 59.5 | 57.8 | | 63.6 | ***Delta*** | |  |
| VSAP9 | July 2021 | NP | 30.3 | 60.1 | 59.1 | 65.9 | 60.7 | 59.3 | 64.2 | ***Delta*** | 59.0 | 59.0 | 65.0 | 60.0 | 58.0 | 64.0 | | ***Delta*** | | 60.7 | | 59.5 | 67.0 | 61.2 | | 60.0 | 64.9 | ***Delta*** | 59.5 | | 58.0 | 65.0 | | 59.6 | 57.8 | | 63.4 | ***Delta*** | |  |
| VSAP10 | July 2021 | NP | 34.7 | 60.2 | 59.3 | 66.3 | 61.0 | 59.5 | 64.5 | ***Delta*** | 60.0 | 59.0 | 65.0 | 60.0 | 58.0 | 64.0 | | ***Delta*** | | 0 | | 0 | 66.9 | 60.9 | | 0 | 64.8 | ***Ind*** | 59.9 | | 58.4 | 65.7 | | 60.1 | 58.2 | | 64.2 | ***Delta*** | |  |
| VSAP11 | July 2021 | NP | 30.1 | 59.6 | 58.9 | 65.6 | 60.4 | 58.9 | 63.8 | ***Delta*** | 59.0 | 58.0 | 65.0 | 59.0 | 58.0 | 63.0 | | ***Delta*** | | 61.2 | | 59.7 | 67.2 | 61.4 | | 59.8 | 65.0 | ***Delta*** | 59.2 | | 57.9 | 65.3 | | 59.9 | 58.3 | | 63.5 | ***Delta*** | |  |
| VSAP12 | July 2021 | NP | 26.5 | 58.9 | 58.8 | 65.6 | 60.3 | 58.9 | 63.7 | ***Delta*** | 59.0 | 58.0 | 65.0 | 59.0 | 58.0 | 64.0 | | ***Delta*** | | 0 | | 0 | 66.9 | 61.1 | | 59.4 | 64.7 | ***Ind*** | 59.5 | | 57.7 | 64.9 | | 59.6 | 57.9 | | 63.3 | ***Delta*** | |  |
| VSAP13 | July 2021 | NP | 30.2 | 59.9 | 59.0 | 65.9 | 60.7 | 59.3 | 64.0 | ***Delta*** | 59.0 | 58.0 | 65.0 | 60.0 | 0 | 64.0 | | ***P-Delta*** | | 0 | | 0 | 66.8 | 61.0 | | 59.3 | 64.8 | ***Delta*** | 59.1 | | 57.8 | 64.9 | | 59.5 | 57.8 | | 63.4 | ***Delta*** | |  |
| VSAP14 | July 2021 | NP | 26.1 | 60.1 | 59.2 | 66.1 | 60.8 | 59.2 | 64.2 | ***Delta*** | 59.0 | 58.5 | 65.0 | 60.0 | 58.0 | 64.0 | | ***Delta*** | | 61.0 | | 59.5 | 66.8 | 61.2 | | 59.8 | 64.7 | ***Delta*** | 59.5 | | 58.0 | 65.3 | | 59.7 | 58.0 | | 63.6 | ***Delta*** | |  |
| VSAP15 | July 2021 | NP | 30.3 | 60.2 | 58.9 | 65.8 | 60.6 | 59.0 | 64.0 | ***Delta*** | 59.0 | 58.0 | 65.0 | 60.0 | 58.0 | 64.0 | | ***Delta*** | | 61.0 | | 59.3 | 66.8 | 61.0 | | 59.5 | 64.7 | ***Delta*** | 59.5 | | 57.8 | 65.1 | | 59.7 | 58.0 | | 63.6 | ***Delta*** | |  |
| VSAP16 | July 2021 | NP | 30.4 | 59.8 | 59.0 | 65.8 | 60.6 | 59.2 | 63.9 | ***Delta*** | 59.0 | 58.0 | 65.0 | 60.0 | 58.0 | 64.0 | | ***Delta*** | | 0 | | 0 | 66.8 | 61.2 | | 59.7 | 64.8 | ***Ind*** | 59.3 | | 58.6 | 65.1 | | 59.7 | 58.2 | | 63.4 | ***Delta*** | |  |
| VSAP17 | July 2021 | NP | 33.9 | 0 | 0 | 65.9 | 60.3 | 0 | 0 | ***Neg*** | 0 | 0 | 0 | 0 | 0 | 0 | | ***Neg*** | | 0 | | 0 | 0 | 0 | | 0 | 0 | ***Neg*** | 0 | | 0 | 0 | | 0 | 0 | | 0 | ***Neg*** | |  |
| VSAP18 | July 2021 | NP | 28.6 | 59.7 | 59.0 | 65.7 | 60.5 | 59.1 | 63.9 | ***Delta*** | 59.0 | 58.0 | 65.0 | 60.0 | 58.0 | 64.0 | | ***Delta*** | | 61.4 | | 59.7 | 67.0 | 61.3 | | 59.8 | 65.0 | ***Delta*** | 59.5 | | 58.4 | 65.2 | | 59.8 | 58.2 | | 63.6 | ***Delta*** | |  |
| VSAP19 | July 2021 | Saliva | ND | 59.3 | 58.6 | 65.2 | 60.1 | 58.5 | 63.4 | ***Delta*** | 59.0 | 58.0 | 65.0 | 59.0 | 58.0 | 64.0 | | ***Delta*** | | 0 | | 0 | 66.7 | 61.0 | | 59.2 | 64.7 | ***Ind*** | 59.5 | | 57.8 | 65.2 | | 59.5 | 57.9 | | 63.3 | ***Delta*** | |  |
| VSAP20 | Aug 2021 | Saliva | ND | 59.4 | 58.5 | 65.3 | 60.1 | 58.5 | 63.3 | ***Delta*** | 59.0 | 58.0 | 65.0 | 60.0 | 58.0 | 64.0 | | ***Delta*** | | 60.8 | | 59.3 | 66.5 | 61.0 | | 59.7 | 64.5 | ***Delta*** | 59.1 | | 58.4 | 65.0 | | 59.7 | 57.8 | | 63.1 | ***Delta*** | |  |
| VSAP21 | Aug 2021 | Saliva | ND | 58.0 | 57.2 | 64.0 | 58.8 | 57.1 | 62.0 | ***Delta*** | 59.0 | 59.0 | 65.0 | 60.0 | 58.0 | 64.0 | | ***Delta*** | | 58.7 | | 57.3 | 64.5 | 58.8 | | 57.5 | 62.5 | ***Delta*** | 59.7 | | 58.4 | 65.5 | | 60.0 | 58.2 | | 63.9 | ***Delta*** | |  |
| VSAP22 | Aug 2021 | Saliva | ND | 59.4 | 58.5 | 65.4 | 60.1 | 58.6 | 63.3 | ***Delta*** | 59.0 | 58.0 | 65.0 | 59.0 | 58.0 | 64.0 | | ***Delta*** | | 0 | | 0 | 66.7 | 60.5 | | 59.5 | 64.5 | ***Ind*** | 59.3 | | 57.8 | 65.0 | | 59.5 | 57.8 | | 63.3 | ***Delta*** | |  |
| VSAP23 | Aug 2021 | Saliva | ND | 58.8 | 58.0 | 64.8 | 59.7 | 58.1 | 62.9 | ***Delta*** | 60.0 | 59.0 | 66.0 | 60.0 | 59.0 | 64.0 | | ***Delta*** | | 62.0 | | 60.2 | 67.5 | 61.7 | | 60.0 | 65.5 | ***Delta*** | 59.7 | | 58.6 | 65.5 | | 60.0 | 58.2 | | 63.9 | ***Delta*** | |  |
| VSAP24 | Aug 2021 | Saliva | 27.2 | 60.8 | 59.4 | 66.3 | 60.9 | 59.0 | 64.4 | ***Delta*** | 0 | 0 | 65.0 | 0 | 0 | 0 | | ***Neg*** | | 0 | | 0 | 66.5 | 0 | | 0 | 0 | ***Ind*** | 0 | | 0 | 65.1 | | 0 | 0 | | 63.9 | ***Ind*** | |  |
| VSAP25 | Aug 2021 | NP | 30.9 | 59.8 | 58.9 | 65.8 | 60.6 | 59.1 | 63.9 | ***Delta*** | 59.0 | 58.0 | 65.0 | 60.0 | 58.0 | 64.0 | | ***Delta*** | | 61.3 | | 59.7 | 67.0 | 61.3 | | 60.0 | 64.9 | ***Delta*** | 59.3 | | 58.0 | 65.2 | | 59.5 | 58.0 | | 63.5 | ***Delta*** | |  |
| VSAP26 | Aug 2021 | Nasal | 31.9 | 60.6 | 59.4 | 66.1 | 61.0 | 59.4 | 64.3 | ***Delta*** | 59.0 | 57.0 | 65.0 | 0 | 0 | 64.0 | | ***P-Delta*** | | 62.0 | | 59.7 | 0 | 0 | | 59.7 | 64.5 | ***P-Delta*** | 60.0 | | 57.6 | 65.8 | | 59.9 | 0 | | 64.4 | ***P-Delta*** | |  |
| VSAP27 | Sep 2021 | NP | 28.7 | 60.0 | 59.2 | 66.0 | 60.7 | 59.3 | 64.1 | ***Delta*** | 59.0 | 58.0 | 65.0 | 60.0 | 58.0 | 64.0 | | ***Delta*** | | 61.0 | | 59.7 | 67.0 | 61.5 | | 60.0 | 65.0 | ***Delta*** | 59.3 | | 58.2 | 65.2 | | 59.9 | 58.2 | | 63.7 | ***Delta*** | |  |
| VSAP28 | Sep 2021 | Nasal | 30.1 | 59.7 | 58.8 | 65.7 | 60.6 | 58.9 | 63.9 | ***Delta*** | 59.0 | 58.0 | 65.0 | 60.0 | 58.0 | 65.0 | | ***Delta*** | | 61.0 | | 59.5 | 66.7 | 61.0 | | 59.7 | 64.5 | ***Delta*** | 59.3 | | 58.0 | 65.2 | | 59.9 | 58.0 | | 63.5 | ***Delta*** | |  |
| VSAP29 | Sep 2021 | Saliva | 30.8 | 58.8 | 58.0 | 64.8 | 59.6 | 58.0 | 63.0 | ***Delta*** | 58.0 | 57.0 | 64.0 | 59.0 | 57.0 | 63.0 | | ***Delta*** | | 59.7 | | 58.5 | 65.7 | 60.0 | | 58.8 | 63.5 | ***Delta*** | 58.3 | | 57.7 | 64.0 | | 58.8 | 57.1 | | 62.3 | ***Delta*** | |  |
| VSAP30 | Sep 2021 | NP | 27.1 | 59.8 | 58.9 | 66.0 | 60.7 | 59.2 | 64.0 | ***Delta*** | 59.0 | 58.0 | 65.0 | 60.0 | 58.0 | 64.0 | | ***Delta*** | | 61.2 | | 59.8 | 67.2 | 61.5 | | 60.4 | 65.1 | ***Delta*** | 59.7 | | 58.4 | 65.5 | | 60.0 | 58.2 | | 63.9 | ***Delta*** | |  |
| VSAP31 | Sep 2021 | NP | 29.0 | 59.9 | 59.1 | 65.8 | 60.6 | 59.0 | 63.8 | ***Delta*** | 59.0 | 58.0 | 65.0 | 60.0 | 58.0 | 64.0 | | ***Delta*** | | 61.0 | | 59.5 | 67.0 | 61.4 | | 60.1 | 65.0 | ***Delta*** | 59.3 | | 58.2 | 65.2 | | 59.9 | 58.2 | | 63.5 | ***Delta*** | |  |
| VSAP32 | Sep 2021 | NP | 29.7 | 59.6 | 58.9 | 65.9 | 60.7 | 59.1 | 63.9 | ***Delta*** | 59.0 | 58.0 | 65.0 | 59.5 | 58.0 | 64.0 | | ***Delta*** | | 61.3 | | 59.9 | 67.3 | 61.5 | | 60.4 | 65.2 | ***Delta*** | 59.5 | | 58.2 | 65.3 | | 60.0 | 58.2 | | 63.7 | ***Delta*** | |  |
| VSAP33 | Sep 2021 | NP | 24.9 | 60.2 | 59.2 | 65.8 | 60.8 | 59.2 | 64.1 | ***Delta*** | 0 | 0 | 65.0 | 59.0 | 57.0 | 64.0 | | ***P-Delta*** | | 0 | | 0 | 67.0 | 61.0 | | 59.7 | 0 | ***Ind*** | 59.3 | | 57.7 | 65.0 | | 59.5 | 57.7 | | 63.3 | ***Delta*** | |  |
| VSAP34 | Oct 2021 | NP | 26.9 | 60.6 | 58.9 | 65.7 | 60.6 | 59.1 | 63.9 | ***Delta*** | 59.0 | 58.0 | 65.0 | 59.0 | 58.0 | 64.0 | | ***Delta*** | | 61.3 | | 59.5 | 66.7 | 61.0 | | 59.5 | 64.5 | ***Delta*** | 59.5 | | 58.0 | 65.0 | | 59.7 | 57.8 | | 63.4 | ***Delta*** | |  |

The Tm values from patient specimens were identified using the VOC identification macro. Samples until VSAP34 were tested in all 4 RT-PCR instruments and Ref-Reference strains; IC-Internal Control; Ind- indeterminate (>2 probe failures); Neg-Negative; Id-Identification; WT-Wild type; MT-Mutant; P- Presumptive.

Supplementary Table 3S. Validation of the SMB-VOC assay using patient specimens in Roche LC480.

| **Reference strains/Patient samples** | **Source** | **Date of collection** | **Initial sample PCR Ct** | **Internal control Ct** | **SMB-501 Assay** | | **SMB-484AKw Assay** | | **SMB-452 Assay** | |  |
| --- | --- | --- | --- | --- | --- | --- | --- | --- | --- | --- | --- |
|  |  |  |  |  | **WT (Cy3)** | **MT  (Cy5)** | **WT (Cy3)** | **MT (Cy5)** | **WT (Cy3)** | **MT (Cy5)** | **Final id by SMB VOC assay** |
| USA-WA1/2020 (WT) | BEI NR52285 | | | | 59.7±0.10 | 58.9±0.07 | 65.5±0.04 | 60.4±0.05 | 61.9±0.08 | 59.9±0.07 | Wild type (Ancestral) |
| B.1.1.7 (Alpha) | BEI NR54000 | | | | 55.7±0.04 | 62.6±0.08 | 65.5±0.12 | 60.4±0.05 | 62.5±0.04 | 60.4±0.03 | B.1.1.7 (Alpha) |
| B.1.351 (Beta) | BEI NR55282 | | | | 55.3±0.06 | 62.5±0.01 | 62.0±0.03 | 64.4±0.04 | 62.5±0.04 | 60.3±0.04 | B.1.351 (Beta) |
| B.1.617.2 (Delta) | BEI NR55611 | | | | 59.6±0.05 | 58.7±0.05 | 65.3±0.04 | 59.9±0.08 | 58.3±0.12 | 63.25±0.04 | B.1.617.2 (Delta) |
| B.1.1.529/BA.1 (Omicron) | BEI NR56461 | | | | 49.0±0.06 | 56.7±0.04 | 63.1±0.04 | 60.3±0.04 | 62.4±0.03 | 59.2±0.04 | B.1.1.529 (Omicron BA.1) |
| B.1.1.529/BA.2 (Omicron) | BEI NR56520 | | | | 50.6±0.10 | 58.5±0.06 | 63.1±0.03 | 60.3±0.03 | 62.5±0.02 | 60.3±0.05 | B.1.1.529 (Omicron BA.2) |
| B.1.1.529/BA.2.12.1 (Omicron) | BEI NR56781 | | | | 50.7 | 58.5 | 62.9 | 60.1 | 60.1 | 61.0 | B.1.1.529 (Omicron BA.2.12.1) |
| B.1.1.529/BA.2 (Omicron) | BEI NR56520 | | | | 50.6 | 58.6 | 62.9 | 60.1 | 62.6 | 60.4 | B.1.1.529 (Omicron BA.2) |
| B.1.1.529/BA.4 (Omicron) | BEI NR56803 | | | | 51.0 | 58.4 | 58.0 | 55.1 | 58.9 | 63.5 | B.1.1.529 (Omicron BA.4/5) |
| B.1.1.529/BA.5 (Omicron) | BEI NR58616 | | | | 51.0 | 58.6 | 57.8 | 54.9 | 58.8 | 63.8 | B.1.1.529 (Omicron BA.4/5) |
| B.1.1.529/BA.4.6 (Omicron) | BEI NR58715 | | | | 51.0 | 58.6 | 57.7 | 54.6 | 58.8 | 63.6 | B.1.1.529 (Omicron BA.4/5) |
| B.1.1.529/BA.4.6 (Omicron) | BEI NR58717 | | | | 50.5 | 58.8 | 58.1 | 55.4 | 58.8 | 63.8 | B.1.1.529 (Omicron BA.4/5) |
| B.1.1.529/BF.5 (Omicron) | BEI NR58716 | | | | 51.0 | 58.5 | 57.9 | 55.1 | 58.8 | 63.8 | B.1.1.529 (Omicron BA.4/5) |
| B.1.1.529/BF.7 (Omicron) | BEI NR58974 | | | | 50.5 | 58.6 | 57.8 | 56.1 | 58.9 | 63.9 | B.1.1.529 (Omicron BA.4/5) |
| B.1.1.529/BQ.1 (Omicron) | BEI NR58975 | | | | 50.5 | 58.6 | 57.9 | 56.2 | 58.7 | 63.9 | B.1.1.529 (Omicron BA.4/5) |
| B.1.1.529/BQ.1.1 (Omicron) | BEI NR58976 | | | | 51.1 | 58.8 | 58.2 | 55.5 | 58.7 | 63.8 | B.1.1.529 (Omicron BA.4/5) |
| B.1.1.529/XBB.1.5 (Omicron) | BEI NR59104 | | | | 50.62 | 58.57 | 57.87 | 55.61 | 62.63 | 60.76 | B.1.1.529 (Omicron XBB.1.5) |
| B.1.1.529/XBB1.9 (Omicron) | BEI NR 59441 | | | | 50.5 | 58.6 | 57.9 | 55.6 | 62.6 | 61.1 | B.1.1.529 (Omicron XBB.1.5) |
| VSAP1 | NP | Apr-21 | 27.7 | 27.7 | 59.6 | 58.7 | 65.6 | 60.4 | 62.7 | 60.4 | Wild type (Ancestral) |
| VSAP2 | NP |  | 30.1 | 29.3 | 55.1 | 62.2 | 65.6 | 60.4 | 62.4 | 60.1 | B.1.1.7 (Alpha) |
| VSAP3 | NP | May-21 | 30.3 | 29.2 | 60.4 | 59.1 | 65.3 | 59.8 | 59.2 | 64.2 | B.1.617.2 (Delta) |
| VSAP4 | NP |  | 32.0 | 25.6 | 55.1 | 62.5 | 65.9 | 60.4 | 62.6 | 59.9 | B.1.1.7 (Alpha) |
| VSAP5 | Saliva |  | 25.7 | 23.7 | 60.4 | 58.5 | 65.8 | 60.5 | 58.5 | 63.5 | B.1.617.2 (Delta) |
| VSAP6 | Saliva |  | 33.2 | 26.9 | 56.1 | 63.5 | 66.2 | 60.7 | 62.8 | 60.6 | B.1.1.7 (Alpha) |
| VSAP7 | NP | Jun-21 | 20.0 | 29.6 | 60.6 | 59.8 | 65.3 | 60.0 | 60.1 | 64.8 | B.1.617.2 (Delta) |
| VSAP8 | NP | Jul-21 | 27.2 | 29.7 | 60.0 | 59.1 | 65.2 | 59.3 | 59.2 | 64.0 | B.1.617.2 (Delta) |
| VSAP9 | NP |  | 28.2 | 30.3 | 60.1 | 59.1 | 65.2 | 59.4 | 59.3 | 64.2 | B.1.617.2 (Delta) |
| VSAP11 | NP |  | 19.5 | 30.1 | 59.6 | 58.9 | 65.6 | 60.0 | 58.9 | 63.8 | B.1.617.2 (Delta) |
| VSAP12 | NP |  | 31.5 | 26.5 | 58.9 | 58.8 | 65.2 | 59.3 | 58.9 | 63.7 | B.1.617.2 (Delta) |
| VSAP13 | NP |  | 23.3 | 30.2 | 59.9 | 59.0 | 65.2 | 59.8 | 59.3 | 64.0 | B.1.617.2 (Delta) |
| VSAP14 | NP |  | 27.4 | 26.1 | 60.1 | 59.2 | 65.2 | 59.6 | 59.2 | 64.2 | B.1.617.2 (Delta) |
| VSAP15 | NP |  | 28.0 | 30.3 | 60.2 | 58.9 | 65.0 | 59.2 | 59.0 | 64.0 | B.1.617.2 (Delta) |
| VSAP16 | NP |  | 21.9 | 30.4 | 59.8 | 59.0 | 65.1 | 59.8 | 59.2 | 63.9 | B.1.617.2 (Delta) |
| VSAP17 | NP |  | 37.6 | 33.9 | No Tm | | | | | | ND |
| VSAP18 | NP |  | 23.3 | 28.6 | 59.7 | 59.0 | 65.3 | 59.8 | 59.1 | 63.9 | B.1.617.2 (Delta) |
| VSAP19 | Saliva |  | 22.7 | 24.4 | 59.3 | 58.6 | 65.0 | 59.2 | 58.5 | 63.4 | B.1.617.2 (Delta) |
| VSAP20 | Saliva | Aug-21 | 26.3 | 24.1 | 59.4 | 58.5 | 65.1 | 59.9 | 58.5 | 63.3 | B.1.617.2 (Delta) |
| VSAP21 | Saliva |  | 15.1 | 25.5 | 58.0 | 57.2 | 65.5 | 59.8 | 57.1 | 62.0 | B.1.617.2 (Delta) |
| VSAP22 | Saliva |  | 26.1 | 28.8 | 59.4 | 58.5 | 64.9 | 59.0 | 58.6 | 63.3 | B.1.617.2 (Delta) |
| VSAP23 | Saliva |  | 18.5 | 23.5 | 58.8 | 58.0 | 65.1 | 59.7 | 58.1 | 62.9 | B.1.617.2 (Delta) |
| VSAP24 | Saliva |  | 36.7 | 27.2 | 60.8 | 59.4 | 65.6 | No Tm | 59.0 | 64.4 | Pres B.1.617.2 (Delta) |
| VSAP25 | NP |  | 18.0 | 30.9 | 59.8 | 58.9 | 65.4 | 59.7 | 59.1 | 63.9 | B.1.617.2 (Delta) |
| VSAP26 | NS |  | 23.2 | 31.9 | 60.6 | 59.4 | 65.0 | 59.3 | 59.4 | 64.3 | B.1.617.2 (Delta) |
| VSAP27 | NP | Sep-21 | 18.1 | 28.7 | 60.0 | 59.2 | 65.4 | 60.0 | 59.3 | 64.1 | B.1.617.2 (Delta) |
| VSAP28 | NS |  | 21.2 | 30.1 | 59.7 | 58.8 | 65.2 | 60.0 | 58.9 | 63.9 | B.1.617.2 (Delta) |
| VSAP29 | Saliva |  | 18.1 | 30.8 | 58.8 | 58.0 | 65.2 | 59.3 | 58.0 | 63.0 | B.1.617.2 (Delta) |
| VSAP30 | NP |  | 17.3 | 27.1 | 59.8 | 58.9 | 65.6 | 59.9 | 59.2 | 64.0 | B.1.617.2 (Delta) |
| VSAP31 | NP |  | 16.5 | 29.0 | 59.9 | 59.1 | 65.3 | 60.0 | 59.0 | 63.8 | B.1.617.2 (Delta) |
| VSAP32 | NP |  | 21.2 | 29.7 | 59.6 | 58.9 | 65.6 | 60.1 | 59.1 | 63.9 | B.1.617.2 (Delta) |
| VSAP33 | NP |  | 30.4 | 24.9 | 60.2 | 59.2 | 65.0 | 59.4 | 59.2 | 64.1 | B.1.617.2 (Delta) |
| VSAP34 | NP | Oct-21 | 23.6 | 26.9 | 60.6 | 58.9 | 65.2 | 59.9 | 59.1 | 63.9 | B.1.617.2 (Delta) |
| VSAP35 | Saliva | Nov-21 | 12.4 | 22.3 | 59.2 | 58.4 | 64.9 | 59.7 | 58.8 | 63.5 | B.1.617.2 (Delta) |
| VSAP36 | NP |  | 28.2 | 28.9 | 59.7 | 59.0 | 65.3 | 59.8 | No Tm | No Tm | VAR IND |
| VSAP37 | Saliva |  | 22.7 | 27.2 | 59.4 | 58.5 | 64.9 | 59.3 | 59.0 | 63.8 | B.1.617.2 (Delta) |
| VSAP38 | NS |  | 30.0 | 34.4 | 60.0 | 58.8 | 65.0 | No Tm | No Tm | No Tm | VAR. IND |
| VSAP39 | NP |  | 29.9 | 32.0 | 59.8 | 58.3 | 65.1 | No Tm | No Tm | No Tm | VAR IND |
| VSAP40 | Saliva |  | 25.2 | 24.7 | 59.6 | 58.4 | 64.8 | 58.9 | 58.9 | 63.9 | B.1.617.2 (Delta) |
| VSAP41 | NP |  | 22.7 | 31.2 | 59.9 | 58.7 | 65.2 | 59.4 | 59.1 | 63.9 | B.1.617.2 (Delta) |
| VSAP42 | Saliva |  | 18.2 | 23.2 | 59.1 | 58.3 | 64.8 | 59.3 | 58.9 | 63.8 | B.1.617.2 (Delta) |
| VSAP43 | NP |  | 29.8 | 34.4 | 59.7 | 58.8 | 65.0 | 59.2 | No Tm | No Tm | VAR IND |
| VSAP44 | NP |  | 29.2 | 32.2 | 59.6 | 58.8 | 65.1 | 59.2 | 59.0 | 64.0 | B.1.617.2 (Delta) |
| VSAP45 | NP | Dec-21 | 28.4 | 32.0 | 60.0 | 58.7 | 64.8 | NP | 58.8 | 64.0 | Pres. B.1.617.2 (Delta) |
| VSAP46 | NP |  | 17.0 | 29.9 | 59.8 | 58.8 | 65.1 | 59.9 | 59.3 | 63.8 | B.1.617.2 (Delta) |
| VSAP47 | NP |  | 22.5 | 28.9 | 60.5 | 58.8 | 65.1 | 59.5 | 58.7 | 63.5 | B.1.617.2 (Delta) |
| VSAP48 | Saliva |  | 26.0 | 25.0 | 59.3 | 58.2 | 65.0 | NP | 58.8 | 63.5 | Pres.B.1.617.2 (Delta) |
| VSAP49 | NP |  | 27.2 | 28.6 | 59.7 | 58.9 | 65.0 | 59.1 | 59.1 | 63.9 | B.1.617.2 (Delta) |
| VSAP50 | NP |  | 20.1 | 30.6 | 59.6 | 58.8 | 65.1 | 59.7 | 59.0 | 63.8 | B.1.617.2 (Delta) |
| VSAP51 | NP |  | 24.1 | 29.9 | 59.2 | 58.4 | 64.8 | 59.3 | 58.5 | 63.3 | B.1.617.2 (Delta) |
| VSAP52 | NP |  | 15.7 | 23.5 | 59.2 | 58.5 | 65.0 | 59.5 | 58.5 | 63.4 | B.1.617.2 (Delta) |
| VSAP53 | NP |  | 27.1 | 30.1 | 59.3 | 58.5 | 64.9 | 59.3 | 58.2 | 63.5 | B.1.617.2 (Delta) |
| VSAP54 | NP |  | 27.3 | 30.0 | 48.9 | 56.5 | 62.7 | 59.8 | 62.3 | 58.9 | B.1.1.529 (Omicron BA.1) |
| VSAP55 | NP |  | 28.4 | 30.8 | 59.1 | 58.4 | 64.8 | 59.6 | 58.5 | 63.4 | B.1.617.2 (Delta) |
| VSAP56 | NP |  | 25.5 | 31.0 | 49.0 | 56.7 | 62.7 | 60.0 | 62.3 | 59.3 | B.1.1.529 (Omicron BA.1) |
| VSAP57 | NP |  | 23.8 | 31.3 | 58.9 | 58.1 | 64.3 | 58.9 | 58.1 | 62.9 | B.1.617.2 (Delta) |
| VSAP58 | NP |  | 22.9 | 30.9 | 59.1 | 58.4 | 64.6 | 59.3 | 58.3 | 63.0 | B.1.617.2 (Delta) |
| VSAP59 | NP |  | 24.2 | 29.9 | 48.9 | 56.5 | 62.8 | 59.9 | 62.3 | 59.0 | B.1.1.529 (Omicron BA.1) |
| VSAP60 | NP |  | 20.4 | 27.6 | 59.3 | 58.6 | 64.8 | 59.7 | 58.6 | 63.4 | B.1.617.2 (Delta) |
| VSAP61 | NP |  | 26.4 | 28.5 | 48.8 | 56.5 | 62.6 | 60.0 | 62.2 | 58.9 | B.1.1.529 (Omicron BA.1) |
| VSAP62 | NP |  | 24.7 | 29.4 | 48.5 | 56.1 | 62.6 | 59.8 | 62.1 | 58.7 | B.1.1.529 (Omicron BA.1) |
| VSAP63 | NP |  | 23.4 | 28.7 | 48.8 | 56.4 | 62.7 | 59.9 | 62.2 | 58.8 | B.1.1.529 (Omicron BA.1) |
| VSAP64 | NP |  | 21.4 | 29.1 | 59.1 | 58.4 | 64.8 | 59.5 | 58.5 | 63.4 | B.1.617.2 (Delta) |
| VSAP65 | NP |  | 24.0 | 28.0 | 48.4 | 56.0 | 62.2 | 59.3 | 61.6 | 58.5 | B.1.1.529 (Omicron BA.1) |
| VSAP66 | NP |  | 21.9 | 32.5 | 59.5 | 58.7 | 64.9 | 59.7 | 58.6 | 63.5 | B.1.617.2 (Delta) |
| VSAP67 | NP |  | 23.1 | 28.4 | 59.3 | 58.5 | 64.8 | 59.7 | 58.6 | 63.4 | B.1.617.2 (Delta) |
| VSAP68 | NP |  | 21.9 | 29.9 | 48.8 | 56.5 | 62.7 | 60.0 | 62.0 | 58.9 | B.1.1.529 (Omicron BA.1) |
| VSAP69 | NP |  | 26.2 | 31.9 | 48.8 | 56.3 | 62.6 | 59.8 | 62.0 | 58.9 | B.1.1.529 (Omicron BA.1) |
| VSAP70 | NP |  | 21.3 | 28.8 | 48.8 | 56.4 | 62.6 | 59.8 | 62.0 | 58.7 | B.1.1.529 (Omicron BA.1) |
| VSAP71 | NP |  | 26.8 | 32.8 | 48.5 | 56.2 | 62.5 | 59.6 | 61.8 | 58.7 | B.1.1.529 (Omicron BA.1) |
| VSAP72 | NP |  | 19.4 | 27.8 | 48.7 | 56.4 | 62.7 | 60.0 | 62.1 | 59.0 | B.1.1.529 (Omicron BA.1) |
| VSAP73 | NP |  | 18.0 | 30.3 | 58.4 | 57.7 | 64.0 | 58.6 | 57.7 | 62.4 | B.1.617.2 (Delta) |
| VSAP74 | NP |  | 18.2 | 29.9 | 48.8 | 56.3 | 62.9 | 60.0 | 62.1 | 58.9 | B.1.1.529 (Omicron BA.1) |
| VSAP75 | NP |  | NK | 34.9 | 50.2 | 58.7 | 58.5 | 55.5 | 58.9 | 63.8 | B.1.1.529 (Omicron BA.4/5) |
| VSAP76 | NP | Jan-22 | 21.5 | 27.0 | 49.0 | 56.8 | 63.1 | 60.4 | 62.5 | 59.1 | B.1.1.529 (Omicron BA.1) |
| VSAP77 | Saliva | Feb-22 | 25.9 | 28.5 | 49.1 | 56.7 | 62.9 | 60.1 | 62.6 | 59.0 | B.1.1.529 (Omicron BA.1) |
| VSAP78 | NP |  | 19.1 | 28.1 | 49.1 | 56.8 | 63.1 | 60.2 | 62.7 | 59.1 | B.1.1.529 (Omicron BA.1) |
| VSAP79 | NP | Mar-22 | 21.6 | 31.4 | 59.8 | 58.8 | 62.8 | 58.3,64.0 | 58.9 | 63.9 | VAR. IND |
| VSAP80 | NP | Apr-22 | 26.2 | 26.5 | 50.7 | 58.7 | 62.9 | 60.0 | 62.9 | 60.3 | B.1.1.529 (Omicron BA.2) |
| VSAP81 | NP |  | 20.4 | 28.1 | 50.8 | 58.7 | 62.7 | 59.8 | 62.7 | 60.4 | B.1.1.529 (Omicron BA.2) |
| VSAP82 | NP |  | 20.9 | 29.9 | 50.7 | 58.7 | 63.0 | 60.3 | 62.7 | 60.4 | B.1.1.529 (Omicron BA.2) |
| VSAP83 | Saliva |  | 29.6 | 27.0 | No Tm | 58.4 | 63.0 | 60.2 | 62.6 | 60.2 | Pres B.1.1.529 (Omicron BA.2) |
| VSAP84 | NP | May-22 | 25.2 | 28.2 | 50.6 | 58.6 | 58.2 | 55.1 | 58.9 | 63.8 | B.1.1.529 (Omicron BA.4/5) |
| VSAP85 | NP |  | 24.9 | 30.7 | 50.8 | 58.8 | 63.0 | 60.3 | 60.1 | 61.3 | B.1.1.529 (Omicron BA.2.12.1) |
| VSAP86 | NP |  | 25.8 | 30.3 | 50.7 | 58.8 | 63.0 | 60.4 | 60.1 | 61.3 | B.1.1.529 (Omicron BA.2.12.1) |
| VSAP87 | NP |  | 21.8 | 30.9 | 50.8 | 58.9 | 63.1 | 60.5 | 60.2 | 61.2 | B.1.1.529 (Omicron BA.2.12.1) |
| VSAP88 | NP | Jun-22 | 17.7 | 28.8 | 50.5 | 58.6 | 63.1 | 60.4 | 62.8 | 60.4 | B.1.1.529 (Omicron BA.2) |
| VSAP89 | NP |  | 20.8 | 26.5 | 50.6 | 58.6 | 63.1 | 60.5 | 62.9 | 60.4 | B.1.1.529 (Omicron BA.2) |
| VSAP90 | NP | July-22 | NK | 27.9 | 50.4 | 58.7 | 58.4 | 55.4 | 58.9 | 63.8 | B.1.1.529 (Omicron BA.4/5) |
| VSAP91 | NP | Sep-22 | NK | 27.2 | 50.4 | 58.4 | 58.0 | 55.2 | 58.7 | 63.8 | B.1.1.529 (Omicron BA.4/5) |
| **Cov-2 Negatives** | | | | | | | | | | | |
| N-VSAP1 | NP | Jan-21 | Neg | 28.9 | No Tm | | | | | | SARS-CoV-2 Not Detected |
| N-VSAP2 | NP | Feb-21 | Neg | 25.0 | No Tm | | | | | | SARS-CoV-2 Not Detected |
| N-VSAP3 | NP |  | Neg | 24.4 | No Tm | | | | | | SARS-CoV-2 Not Detected |
| N-VSAP4 | NP |  | Neg | 22.9 | No Tm | | | | | | SARS-CoV-2 Not Detected |
| N-VSAP5 | NP |  | Neg | 23.7 | No Tm | | | | | | SARS-CoV-2 Not Detected |
| N-VSAP6 | NP |  | Neg | 29.9 | No Tm | | | | | | SARS-CoV-2 Not Detected |
| N-VSAP7 | NP |  | Neg | 29.3 | No Tm | | | | | | SARS-CoV-2 Not Detected |
| N-VSAP8 | NP |  | Neg | 25.5 | No Tm | | | | | | SARS-CoV-2 Not Detected |
| N-VSAP9 | NP |  | Neg | 25.2 | No Tm | | | | | | SARS-CoV-2 Not Detected |

Ct-Cycle threshold; NP-Nasopharyngeal swab specimen; NS-Nasal Swab specimen; NK- not known; Neg-Negative;
VAR IND- Variant indeterminate

Pres.: Presumptive (presumptive call is made when one or more probes in the assay fails to give a Tm)

Supplementary Table 4S. Confirmation of mutations in patient samples by sequencing.

| **Reference strains/Patient samples** | **SMB genotyping assay Id** | **Confirmation by Sanger sequencing** | | | |
| --- | --- | --- | --- | --- | --- |
|  |  | **501 probe binding region (Amino acid and position)** | **484 probe binding region (Amino acid and position)** | **452 probe binding region (Amino acid and position)** | **Sequencing id** |
| USA-WA1/2020 (WT) | | AAT (N501) | GAA (E484) | CTG (L) | WT |
| B.1.1.7 (Alpha) | | TAT (Y501) | GAA (E484) | CTG (L) | B.1.1.7 (Alpha) |
| B.1.351 (Beta) | | TAT (Y501) | AAA (K484) | CTG (L) | B.1.351 (Beta) |
| B.1.617.2 (Delta) | | AAT (N501) | GAA (E484) | CGG (R) | B.1.617.2 (Delta) |
| B.1.1.529 (Omicron BA.1) | | AGT (S496); CGA (R498); TAT(Y501); CAC (H505) | GCA (A484) | CTG (L) | B.1.1.529 (Omicron BA.1) |
| B.1.1.529 (Omicron BA.2) | | CGA (R498); TAT (Y501); CAC (H505) | GCA (A484) | CTG (L) | B.1.1.529 (Omicron BA.2) |
| B.1.1.529 (Omicron BA.2.12.1) | | CGA (R498); TAT (Y501); CAC (H505) | GCA (A484) | CAG (Q) | B.1.1.529 (Omicron BA.2.12.1) |
| B.1.1.529 (Omicron BA.4/5) | | CGA (R498); TAT (Y501); CAC (H505) | GCA (A484); GTT (F486V) | CGG (R) | B.1.1.529 (Omicron BA.4/5) |
| VSAP1 | WT | AAT (N) | GAA (E) | CTG (L) | WT |
| VSAP2 | B.1.1.7 (Alpha) | TAT (Y) | GAA (E) | CTG (L) | B.1.1.7 (Alpha) |
| VSAP3 | B.1.617.2 (Delta) | AAT (N) | GAA (E) | CGG (R) | B.1.617.2 (Delta) |
| VSAP4 | B.1.1.7 (Alpha) | TAT (Y) | GAA (E) | CTG (L) | B.1.1.7 (Alpha) |
| VSAP5 | B.1.617.2 (Delta) | AAT (N) | GAA (E) | CGG (R) | B.1.617.2 (Delta) |
| VSAP6 | B.1.1.7 (Alpha) | TAT (Y) | GAA (E) | CTG (L) | B.1.1.7 (Alpha) |
| VSAP7 | B.1.617.2 (Delta) | AAT (N) | GAA (E) | CGG (R) | B.1.617.2 (Delta) |
| VSAP8 | B.1.617.2 (Delta) | AAT (N) | GAA (E) | CGG (R) | B.1.617.2 (Delta) |
| VSAP9 | B.1.617.2 (Delta) | AAT (N) | GAA (E) | CGG (R) | B.1.617.2 (Delta) |
| VSAP11 | B.1.617.2 (Delta) | AAT (N) | GAA (E) | CGG (R) | B.1.617.2 (Delta) |
| VSAP12 | B.1.617.2 (Delta) | AAT (N) | GAA (E) | CGG (R) | B.1.617.2 (Delta) |
| VSAP13 | B.1.617.2 (Delta) | AAT (N) | GAA (E) | CGG (R) | B.1.617.2 (Delta) |
| VSAP14 | B.1.617.2 (Delta) | AAT (N) | GAA (E) | CGG (R) | B.1.617.2 (Delta) |
| VSAP15 | B.1.617.2 (Delta) | AAT (N) | GAA (E) | CGG (R) | B.1.617.2 (Delta) |
| VSAP16 | B.1.617.2 (Delta) | AAT (N) | GAA (E) | CGG (R) | B.1.617.2 (Delta) |
| VSAP17 | Negative |  |  |  | ND |
| VSAP18 | B.1.617.2 (Delta) | AAT (N) | GAA (E) | CGG (R) | B.1.617.2 (Delta) |
| VSAP19^a^ | B.1.617.2 (Delta) | Poor quality sequence | | | |
| VSAP20 | B.1.617.2 (Delta) | AAT (N) | GAA (E) | CGG (R) | B.1.617.2 (Delta) |
| VSAP21 | B.1.617.2 (Delta) | AAT (N) | GAA (E) | CGG (R) | B.1.617.2 (Delta) |
| VSAP22 | B.1.617.2 (Delta) | AAT (N) | GAA (E) | CGG (R) | B.1.617.2 (Delta) |
| VSAP23^a^ | B.1.617.2 (Delta) | Poor quality sequence | | | |
| VSAP24^a^ | B.1.617.2 (Delta) |  |  |  |  |
| VSAP25 | B.1.617.2 (Delta) | AAT (N) | GAA (E) | CGG (R) | B.1.617.2 (Delta) |
| VSAP26 | B.1.617.2 (Delta) | AAT (N) | GAA (E) | CGG (R) | B.1.617.2 (Delta) |
| VSAP27 | B.1.617.2 (Delta) | AAT (N) | GAA (E) | CGG (R) | B.1.617.2 (Delta) |
| VSAP28 | B.1.617.2 (Delta) | AAT (N) | GAA (E) | CGG (R) | B.1.617.2 (Delta) |
| VSAP29^a^ | B.1.617.2 (Delta) | Sequencing failed | | | |
| VSAP30 | B.1.617.2 (Delta) | AAT (N) | GAA (E) | CGG (R) | B.1.617.2 (Delta) |
| VSAP31 | B.1.617.2 (Delta) | AAT (N) | GAA (E) | CGG (R) | B.1.617.2 (Delta) |
| VSAP32 | B.1.617.2 (Delta) | AAT (N) | GAA (E) | CGG (R) | B.1.617.2 (Delta) |
| VSAP33 | B.1.617.2 (Delta) | AAT (N) | GAA (E) | CGG (R) | B.1.617.2 (Delta) |
| VSAP34 | B.1.617.2 (Delta) | AAT (N) | GAA (E) | CGG (R) | B.1.617.2 (Delta) |
| VSAP35 | B.1.617.2 (Delta) | AAT (N) | GAA (E) | CGG (R) | B.1.617.2 (Delta) |
| VSAP36 | VARIANT INDETERMINATE | AAT (N) | GAA (E) | CGG (R) | B.1.617.2 (Delta) |
| VSAP37 | B.1.617.2 (Delta) | AAT (N) | GAA (E) | CGG (R) | B.1.617.2 (Delta) |
| VSAP38 | VARIANT INDETERMINATE | AAT (N) | GAA (E) | CGG (R) | B.1.617.2 (Delta) |
| VSAP39 | VARIANT INDETERMINATE | AAT (N) | GAA (E) | CGG (R) | B.1.617.2 (Delta) |
| VSAP40 | Pres B.1.617.2 (Delta) | AAT (N) | GAA (E) | CGG (R) | B.1.617.2 (Delta) |
| VSAP41 | Pres B.1.617.2 (Delta) | AAT (N) | GAA (E) | CGG (R) | B.1.617.2 (Delta) |
| VSAP42 | B.1.617.2 (Delta) | AAT (N) | GAA (E) | CGG (R) | B.1.617.2 (Delta) |
| VSAP43 | VARIANT INDETERMINATE | AAT (N) | GAA (E) | CGG (R) | B.1.617.2 (Delta) |
| VSAP44 | B.1.617.2 (Delta) | AAT (N) | GAA (E) | CGG (R) | B.1.617.2 (Delta) |
| VSAP45 | B.1.617.2 (Delta) | AAT (N) | GAA (E) | CGG (R) | B.1.617.2 (Delta) |
| VSAP46 | B.1.617.2 (Delta) | AAT (N) | GAA (E) | CGG (R) | B.1.617.2 (Delta) |
| VSAP47 | Pres B.1.617.2 (Delta) | AAT (N) | GAA (E) | CGG (R) | B.1.617.2 (Delta) |
| VSAP48 | B.1.617.2 (Delta) | AAT (N) | GAA (E) | CGG (R) | B.1.617.2 (Delta) |
| VSAP49 | B.1.617.2 (Delta) | AAT (N) | GAA (E) | CGG (R) | B.1.617.2 (Delta) |
| VSAP50 | B.1.617.2 (Delta) | AAT (N) | GAA (E) | CGG (R) | B.1.617.2 (Delta) |
| VSAP51 | B.1.617.2 (Delta) | AAT (N) | GAA (E) | CGG (R) | B.1.617.2 (Delta) |
| VSAP52 | B.1.617.2 (Delta) | AAT (N) | GAA (E) | CGG (R) | B.1.617.2 (Delta) |
| VSAP53 | B.1.617.2 (Delta) | AAT (N) | GAA (E) | CGG (R) | B.1.617.2 (Delta) |
| VSAP54 | B.1.1.529 (Omicron BA.1) | AGT (S); CGA (R); TAT (Y); CAC (H) | GCA (A) | CTG (L) | B.1.1.529 (Omicron BA.1) |
| VSAP55 | B.1.617.2 (Delta) | AAT (N) | GAA (E) | CGG (R) | B.1.617.2 (Delta) |
| VSAP56 | Pres B.1.1.529 (Omicron BA.1) | AGT (S); CGA (R); TAT (Y); CAC (H) | GCA (A) | CTG (L) | B.1.1.529 (Omicron BA.1) |
| VSAP57 | B.1.617.2 (Delta) | AAT (N) | GAA (E) | CGG (R) | B.1.617.2 (Delta) |
| VSAP58 | B.1.617.2 (Delta) | AAT (N) | GAA (E) | CGG (R) | B.1.617.2 (Delta) |
| VSAP59 | B.1.1.529 (Omicron BA.1) | AGT (S); CGA (R); TAT (Y); CAC (H) | GCA (A) | CTG (L) | B.1.1.529 (Omicron BA.1) |
| VSAP60 | B.1.617.2 (Delta) | AAT (N) | GAA (E) | CGG (R) | B.1.617.2 (Delta) |
| VSAP61 | B.1.1.529 (Omicron BA.1) | AGT (S); CGA (R); TAT (Y); CAC (H) | GCA (A) | CTG (L) | B.1.1.529 (Omicron BA.1) |
| VSAP62 | B.1.1.529 (Omicron BA.1) | AGT (S); CGA (R); TAT (Y); CAC (H) | GCA (A) | CTG (L) | B.1.1.529 (Omicron BA.1) |
| VSAP63 | B.1.1.529 (Omicron BA.1) | AGT (S); CGA (R); TAT (Y); CAC (H) | GCA (A) | CTG (L) | B.1.1.529 (Omicron BA.1) |
| VSAP64 | B.1.617.2 (Delta) | AAT (N) | GAA (E) | CGG (R) | B.1.617.2 (Delta) |
| VSAP65 | B.1.1.529 (Omicron BA.1) | AGT (S); CGA (R); TAT (Y); CAC (H) | GCA (A) | CTG (L) | B.1.1.529 (Omicron BA.1) |
| VSAP66 | B.1.617.2 (Delta) | AAT (N) | GAA (E) | CGG (R) | B.1.617.2 (Delta) |
| VSAP67 | B.1.617.2 (Delta) | AAT (N) | GAA (E) | CGG (R) | B.1.617.2 (Delta) |
| VSAP68 | B.1.1.529 (Omicron BA.1) | AGT (S); CGA (R); TAT (Y); CAC (H) | GCA (A) | CTG (L) | B.1.1.529 (Omicron BA.1) |
| VSAP69 | B.1.1.529 (Omicron BA.1) | AGT (S); CGA (R); TAT (Y); CAC (H) | GCA (A) | CTG (L) | B.1.1.529 (Omicron BA.1) |
| VSAP70 | B.1.1.529 (Omicron BA.1) | AGT (S); CGA (R); TAT (Y); CAC (H) | GCA (A) | CTG (L) | B.1.1.529 (Omicron BA.1) |
| VSAP71 | Pres B.1.1.529 (Omicron BA.1) | AGT (S); CGA (R); TAT (Y); CAC (H) | GCA (A) | CTG (L) | B.1.1.529 (Omicron BA.1) |
| VSAP72 | B.1.1.529 (Omicron BA.1) | AGT (S); CGA (R); TAT (Y); CAC (H) | GCA (A) | CTG (L) | B.1.1.529 (Omicron BA.1) |
| VSAP73 | B.1.617.2 (Delta) | AAT (N) | GAA (E) | CGG (R) | B.1.617.2 (Delta) |
| VSAP74 | B.1.1.529 (Omicron BA.1) | AGT (S); CGA (R); TAT (Y); CAC (H) | GCA (A) | CTG (L) | B.1.1.529 (Omicron BA.1) |
| VSAP75 | B.1.1.529 (Omicron BA.4/5) | CGA (R); TAT (Y); CAC (H) | GCA (A); GTT (V) | CGG (R) | B.1.1.529 (Omicron BA.5.2)^b^ |
| VSAP76 | B.1.1.529 (Omicron BA.1) | AGT (S); CGA (R); TAT (Y); CAC (H) | GCA (A) | CTG (L) | B.1.1.529 (Omicron BA.1.1)^b^ |
| VSAP77 | B.1.1.529 (Omicron BA.1) | AGT (S); CGA (R); TAT (Y); CAC (H) | GCA (A) | CTG (L) | B.1.1.529 (Omicron BA.1) |
| VSAP78 | B.1.1.529 (Omicron BA.1) | AGT (S); CGA (R); TAT (Y); CAC (H) | GCA (A) | CTG (L) | B.1.1.529 (Omicron BA.1) |
| VSAP79 | VARIANT INDETERMINATE | CAA (Q, WT); AAT (N, WT); TAC (Y, WT) | GYT (?), GAA (E) | CGG (R) | Inconclusive* ^b^ |
| VSAP80 | B.1.1.529 (Omicron BA.2) | CGA (R); TAT (Y); CAC (H) | GCA (A) | CTG (L) | B.1.1.529 (Omicron BA.2)^b^ |
| VSAP81 | B.1.1.529 (Omicron BA.2) | CGA (R); TAT (Y); CAC (H) | GCA (A) | CTG (L) | B.1.1.529 (Omicron BA.2) |
| VSAP82 | B.1.1.529 (Omicron BA.2) | CGA (R); TAT (Y); CAC (H) | GCA (A) | CTG (L) | B.1.1.529 (Omicron BA.2) |
| VSAP83 | Pres B.1.1.529 (Omicron BA.2) | CGA (R); TAT (Y); CAC (H) | GCA (A) | CTG (L) | B.1.1.529 (Omicron BA.2) |
| VSAP84 | B.1.1.529 (Omicron BA.4/5) | CGA (R); TAT (Y); CAC (H) | GCA (A); GTT (V) | CGG (R) | B.1.1.529 (Omicron BA.4.2)^b^ |
| VSAP85 | B.1.1.529 (Omicron BA.2.12.1) | CGA (R); TAT (Y); CAC (H) | GCA (A) | CAG (Q) | B.1.1.529 (Omicron BA.2.12.1)^b^ |
| VSAP86 | B.1.1.529 (Omicron BA.2.12.1) | CGA (R); TAT (Y); CAC (H) | GCA (A) | CAG (Q) | B.1.1.529 (Omicron BA.2.12.1) |
| VSAP87 | B.1.1.529 (Omicron BA.2.12.1) | CGA (R); TAT (Y); CAC (H) | GCA (A) | CAG (Q) | B.1.1.529 (Omicron BA.2.12.1) |
| VSAP88 | B.1.1.529 (Omicron BA.2) | CGA (R); TAT (Y); CAC (H) | GCA (A) | CTG (L) | B.1.1.529 (Omicron BA.2) |
| VSAP89 | B.1.1.529 (Omicron BA.2) | CGA (R); TAT (Y); CAC (H) | GCA (A) | CTG (L) | B.1.1.529 (Omicron BA.2) |
| VSAP90 | B.1.1.529 (Omicron BA.4/5) | CGA (R); TAT (Y); CAC (H) | GCA (A); GTT (V) | CGG (R) | B.1.1.529 (Omicron BA.5.5)^b^ |
| VSAP91 | B.1.1.529 (Omicron BA.4/5) | CGA (R); TAT (Y); CAC (H) | GCA (A); GTT (V) | CGG (R) | B.1.1.529 (Omicron BA.4/5) |

*Sanger sequencing showed inconclusive mutation (GAA-GYA) at the 484 codon. Y=C/T; if GAA-GTA it would be WT, if GAA-GCA it is an undefined mutation that does not match with the reference sequences. However, the strain was confirmed as Delta AY.25.1 by whole genome sequencing.

^a^Sanger sequencing returned bad quality or no sequences and not enough sample left to repeat.

^b^confirmed by whole genome sequencing

Pres.: Presumptive (presumptive call is made when one or more probes in the assay fails to give a Tm)

ND: not done


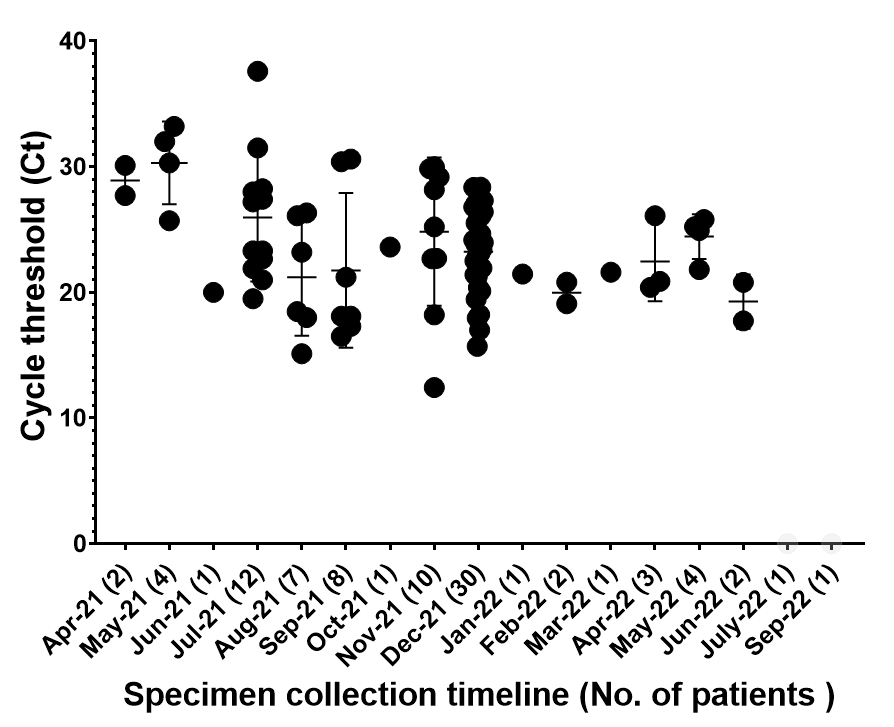


Supplementary Fig. 1S. Patient specimen timeline and Cycle threshold (Ct) values at collection


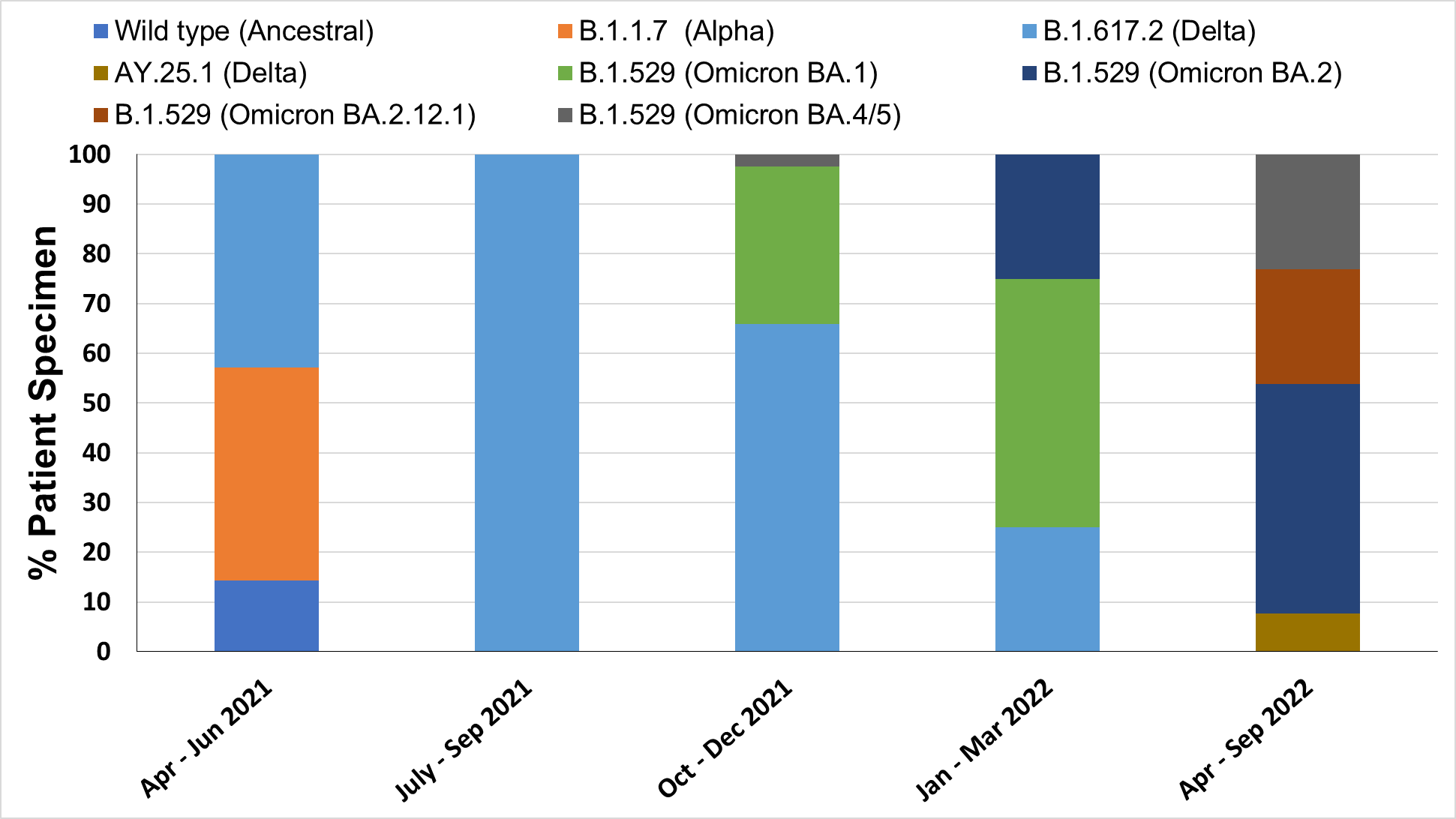


Supplementary Fig. 2S. Prevalence of SARS-CoV-2 variant strains among the tested sample set over the period of 16 months graphed for every 3-month period. The proportion of the variants is shown for specimens obtained during the periods of April-June 2001 (N=7); July-September 2021 (n=23); October-December 2021(n=41); January-March 2022 (n=4) and April-September 2022 (n=13) Since there was only one sample, the July and September sample was included in the last trimester.


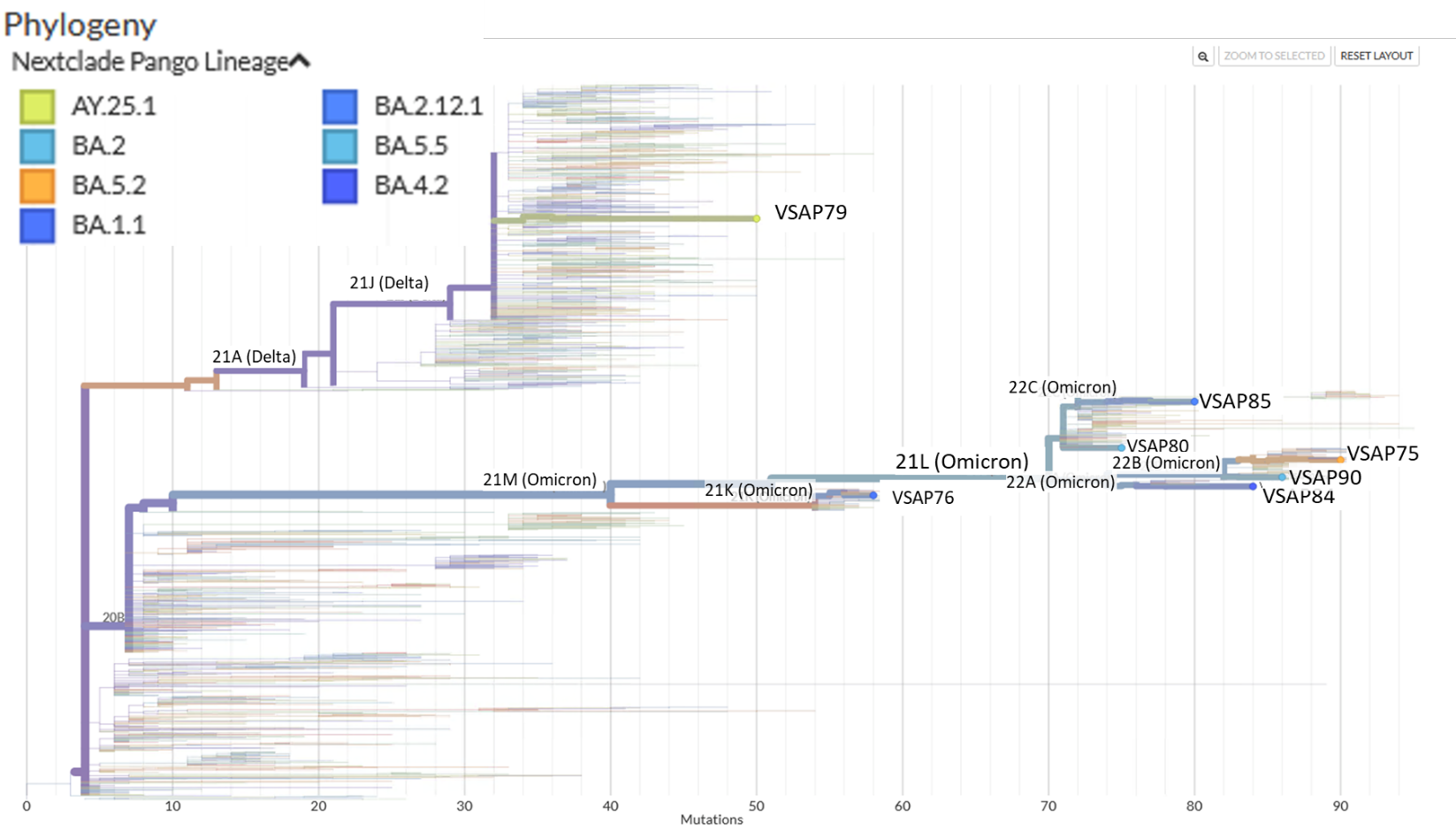


Supplementary Fig. 3S. Phylogenetic analysis of the whole genome sequencing data of the representative patient specimens to establish the accuracy of the variants identified by SMB-VOC assay.
