# Supplementary material for: An expanded RT-PCR melting temperature coding assay to rapidly identify all known SARS-CoV-2 variants and sub-variants of concern
Source: Sci Rep. 2023 Dec 11;13:21927. doi: 10.1038/s41598-023-48647-8 (PMC10713575; doi:10.1038/s41598-023-48647-8)
Supplement: Supplementary file 4 — Supplementary Information 4. [file 41598_2023_48647_MOESM4_ESM.pdf]

*isolate	GeneBank Accession	SRA
VSAP1	SAMN33948603	
VSAP2	SAMN33948604	
VSAP3	SAMN33948605	
VSAP4	SAMN33948606	
VSAP5	SAMN33948607	
VSAP6	SAMN33948608	
VSAP7	SAMN33948609	
VSAP8	SAMN33948610	
VSAP9	SAMN33948611	
VSAP11	SAMN33948612	
VSAP12	SAMN33948613	
VSAP13	SAMN33948614	
VSAP14	SAMN33948615	
VSAP15	SAMN33948616	
VSAP16	SAMN33948617	
VSAP18	SAMN33948618	
VSAP20	SAMN33948619	
VSAP21	SAMN33948620	
VSAP22	SAMN33948621	
VSAP25	SAMN33948622	
VSAP27	SAMN33948623	
VSAP28	SAMN33948624	
VSAP30	SAMN33948625	
VSAP31	SAMN33948626	
VSAP32	SAMN33948627	
VSAP33	SAMN33948628	
VSAP34	SAMN33948629	
VSAP35	SAMN33948630	
VSAP36	SAMN33948631	
VSAP37	SAMN33948632	
VSAP38	SAMN33948633	
VSAP39	SAMN33948634	
VSAP40	SAMN33948635	
VSAP41	SAMN33948636	
VSAP42	SAMN33948637	
VSAP43	SAMN33948638	
VSAP44	SAMN33948639	
VSAP45	SAMN33948640	
VSAP46	SAMN33948641	
VSAP47	SAMN33948642	
VSAP48	SAMN33948643	
VSAP49	SAMN33948644	
VSAP50	SAMN33948645	
VSAP51	SAMN33948646	
VSAP52	SAMN33948647	
VSAP53	SAMN33948648	

VSAP54	SAMN33948649	
VSAP55	SAMN33948650	
VSAP56	SAMN33948651	
VSAP57	SAMN33948652	
VSAP58	SAMN33948653	
VSAP59	SAMN33948654	
VSAP60	SAMN33948655	
VSAP61	SAMN33948656	
VSAP62	SAMN33948657	
VSAP63	SAMN33948658	
VSAP64	SAMN33948659	
VSAP65	SAMN33948660	
VSAP66	SAMN33948661	
VSAP67	SAMN33948662	
VSAP68	SAMN33948663	
VSAP69	SAMN33948664	
VSAP70	SAMN33948665	
VSAP71	SAMN33948666	
VSAP72	SAMN33948667	
VSAP73	SAMN33948668	
VSAP74	SAMN33948669	
VSAP75	SAMN33829847	SRR23929220
VSAP76	SAMN33829749	SRR23929225
VSAP77	SAMN33948670	
VSAP78	SAMN33948671	
VSAP79	SAMN33829747	SRR23929226
VSAP80	SAMN33830192	SRR23929224
VSAP81	SAMN33948672	
VSAP82	SAMN33948673	
VSAP83	SAMN33948674	
VSAP84	SAMN33829839	SRR23929222
VSAP85	SAMN33829829	SRR23929223
VSAP86	SAMN33948675	
VSAP87	SAMN33948676	
VSAP88	SAMN33948677	
VSAP89	SAMN33948678	
VSAP90	SAMN33829840	SRR23929221
VSAP91	SAMN33948679	
